# Xylosyltransferase engineering to manipulate proteoglycans in mammalian cells

**DOI:** 10.1038/s41589-025-02113-w

**Published:** 2026-01-20

**Authors:** Zhen Li, Himanshi Chawla, Lucia Di Vagno, Aisling Ní Cheallaigh, Meg Critcher, Douglas Sammon, Edgar Gonzalez-Rodriguez, David C. Briggs, Nara Chung, Vincent Chang, Keira E. Mahoney, Anna Cioce, Ganka Bineva-Todd, Pei-Ying Wang, Yi-Chang Liu, Lloyd D. Murphy, Yen-Hsi Chen, Yoshiki Narimatsu, Rebecca L. Miller, Lianne I. Willems, Stacy A. Malaker, Mia L. Huang, Gavin J. Miller, Erhard Hohenester, Benjamin Schumann

**Affiliations:** 1 Department of Chemistry, Imperial College London, London, UK; 2 Chemical Glycobiology Laboratory, The Francis Crick Institute, London, UK; 3 Proteomics Science Technology Platform, The Francis Crick Institute, London, UK; 4 Lennard-Jones Laboratory, School of Chemical and Physical Sciences and Centre for Glycoscience, Keele University, Keele, UK; 5 Manchester Institute of Biotechnology and Department of Chemistry, University of Manchester, Manchester, UK; 6 Skaggs Graduate School of Chemical and Biological Sciences, Scripps Research Institute, La Jolla, CA USA; 7 Department of Chemistry, Scripps Research Institute, La Jolla, CA USA; 8 Department of Life Sciences, Imperial College London, London, UK; 9 Signalling and Structural Biology Laboratory, The Francis Crick Institute, London, UK; 10 Department of Chemistry, Yale University, New Haven, CT USA; 11 Glycogenetics, Inc., Taipei, Taiwan; 12 York Structural Biology Laboratory and York Biomedical Research Institute, Department of Chemistry, University of York, York, UK; 13 Copenhagen Center for Glycomics, Department of Cellular and Molecular Medicine, Faculty of Health Sciences, University of Copenhagen, Copenhagen, Denmark; 14 Faculty of Chemistry and Food Chemistry, TUD Dresden University of Technology, Dresden, Germany; 15 Present Address: Avacta, London, UK; 16 Present Address: GlycoDisplay ApS, Copenhagen, Denmark

**Keywords:** Glycobiology, Post-translational modifications

## Abstract

Mammalian cells receive signaling instructions through interactions on their surfaces. Proteoglycans are critical to these interactions, carrying long glycosaminoglycans that recruit signaling molecules. Biosynthetic redundancy in the first glycosylation step by two xylosyltransferases XT1/2 complicates annotation of proteoglycans. Here we develop a chemical genetic strategy that manipulates the glycan attachment site of cellular proteoglycans. Through a bump-and-hole tactic, we engineer the two isoenzymes XT1 and XT2 to specifically transfer the chemically tagged xylose analog 6AzGlc to target proteins. The tag contains a bioorthogonal functionality, allowing to visualize and profile target proteins in mammalian cells. Unlike xylose analogs, 6AzGlc is amenable to cellular nucleotide-sugar biosynthesis, establishing the XT1/2 bump-and-hole tactic in cells. The approach allows pinpointing glycosylation sites by mass spectrometry and exploiting the chemical handle to manufacture proteoglycans with defined glycosaminoglycan chains for cellular applications. Engineered XT enzymes permit an orthogonal view into proteoglycan biology through conventional techniques in biochemistry.

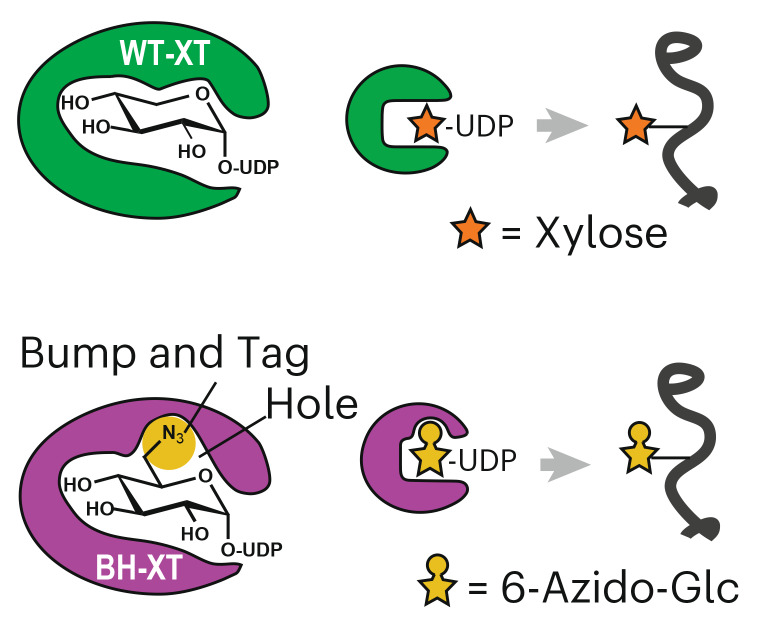

## Abstract (web summary)

The xylosyltransferase isoenzymes XT1 and XT2 catalyze the first glycosylation step in the biosynthesis of proteoglycans. Now, bump-and-hole engineering of XT1 and XT2 enables substrate profiling and modification of proteins as designer proteoglycans to modulate cellular behavior.

## Main

Proteoglycans are large biomolecules that consist of a core protein and one or more glycosaminoglycan (GAG) modifications on cell surfaces and within the extracellular matrix in metazoa^1,2^. Growth factors, neurotrophic factors and chemokines can be recruited to target cells through GAG-binding sites, rendering proteoglycans important determinants for development^3,4^. Consequently, dysfunctions in GAG biosynthesis cause severe phenotypes from embryonic lethality to skeletal and muscular deficiencies^5^. Binding events between proteoglycans and their receptors are impacted by the core protein and the identity of GAGs, which are classified into heparan sulfate (HS), chondroitin sulfate (CS), dermatan sulfate and keratan sulfate^6,7^. Biochemistry and genetic engineering have linked proteoglycan physiology to the GAG structures on particular cell types or even on distinct subcellular locations^8–13^. Despite their relevance in physiology, only a relatively small number of proteoglycans (<100) are known in humans^14,15^. Furthermore, it is challenging to dissect the role of the protein backbone in proteoglycan physiology from the role of the GAG chain, necessitating strategies in chemistry to manipulate and alter proteoglycans^16^.

An impediment for profiling proteoglycans is the large size of GAG modifications that renders analysis by mass spectrometry (MS) challenging. While GAG-carrying glycopeptides contain common amino acid signatures such as acidic patches and a central *O*-glycosylated Ser with often flanking Gly or Ala residues, there is no consensus sequence to predict GAG glycosylation in the Golgi^6,9,17,18^. Common strategies to identify proteoglycans feature enzymatic digestion of GAG chains either before or after isolation of glycopeptides^9,19–24^. While powerful, such procedures make use of complex digestion and purification protocols and focus solely on the GAG-carrying glycopeptide, without the advantages of shotgun (glyco)proteomics methods that use the full MS peptide coverage of individual proteins for detection.

**Fig. 1:**
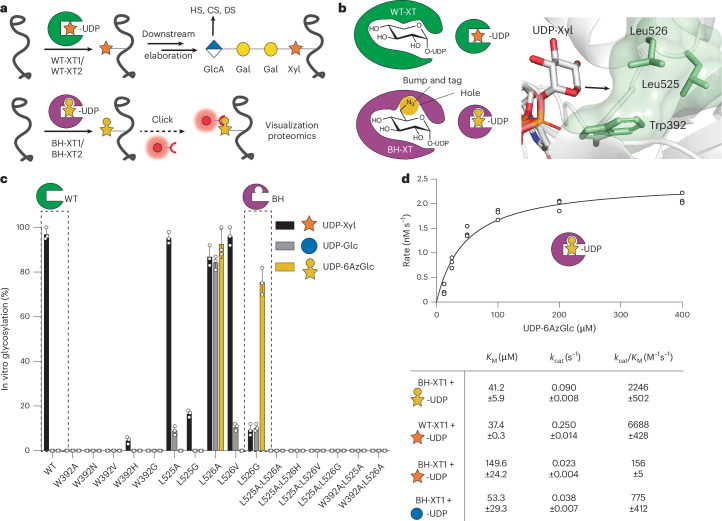
Design of a xylosyltransferase BH system. **a**, Principle of the BH approach. WT-XTs transfer Xyl to substrate proteins that can be extended to GAG chains. BH-XTs transfer a bioorthogonal Xyl analog for visualization and MS profiling. **b**, Structural considerations of XT1 engineering to accept UDP-6AzGlc instead of UDP-Xyl. Insert, gatekeeper residues in the XT1 crystal structure (PDB 6EJ7) and structural trajectory of the azidomethyl modification in UDP-6AzGlc. **c**, In vitro glycosylation of a fluorescently labeled bikunin substrate peptide by WT-XT1 or mutant XT1 and different UDP-sugars (UDP-Xyl in black, UDP-Glc in light gray and UDP-6AzGlc in yellow). Data are individual data points, representing the mean ± s.d. from *n* = 3 technical replicates from one of two independent experiments. **d**, Michaelis–Menten kinetics of in vitro peptide glycosylation by different XT1–UDP-sugar combinations. Data are one independent replicate with *n* = 3 technical replicates, representing the average ± range from two independent replicates each or mean ± s.e.m. of *n* = 3 independent replicates (BH-XT1–UDP-Glc). Source data

The biosynthesis of HS and CS commences through a common *O*-linked glycan ‘linker’ modification consisting of a glucuronic acid (GlcA), two galactoses (Gal) and a xylose (Xyl) in the GlcA(β-3)Gal(β-3)Gal(β-4)Xyl(β-)Ser sequence (Fig. 1a), with optional further modifications^7,25^. The first glycosylation step attaching Xyl to Ser is subject to redundancy by the xylosyltransferase isoenzymes XT1 and XT2 that use uridine diphosphate (UDP)-Xyl as a substrate. The isoenzymes share 60% amino acid identity but display tissue-specific expression patterns. Dysfunctions are associated with different genetic disorders: Desbuquois Dysplasia Type 2 and Spondylo-ocular syndrome for *XYLT1* and *XYLT2* mutations, respectively^26–29^. Differential roles in physiology have been attributed to XT1 and XT2 (refs. ^30,31^). Although XT2 appears to be the dominant isoenzyme in cell lines and serum^32,33^, mice with *Xylt1* and *Xylt2* knockout (KO) display differential defects in development^30,31^. It is currently not possible to directly profile the substrate proteins or even individual glycosylation sites of XT isoenzymes in cells or in vivo.

Here, we use a chemical biology tactic termed bump-and-hole (BH) engineering to probe the substrates of human xylosyltransferases in living cells. On the basis of the structural considerations, we replace a bulky amino acid in the active site of XT1 with a smaller residue to accept the chemically modified substrate UDP-6AzGlc that is not accepted by the wild-type (WT) enzyme. The chemical modification contains an azide group for bioorthogonal incorporation of fluorophores or biotin (Fig. 1a). Judicious choice of the analog as a derivative of UDP-glucose (Glc) enables cytosolic delivery, circumventing the lack of a cellular salvage pathway for ‘direct’ analogs of Xyl. After in-depth biochemical characterization, we install the BH-XT1 system in mammalian cells to directly visualize, profile and probe proteoglycans. We further show that BH engineering can be applied to the isoenzyme XT2, allowing differential substrate profiling of both isoenzymes in mammalian cells. Introduction of a chemical handle at the native glycosylation site enables attachment of a bioorthogonally tagged GAG chain, furnishing ‘designer’ proteoglycans to modulate cellular behavior. Through an XT BH system, we chemically manipulate proteoglycans in mammalian cells for functional evaluation.

## Results

### Design of a xylosyltransferase BH system

Our XT BH design was prompted by biosynthetic and structural considerations (Fig. 1b). In the absence of a functional group that allows facile chemical modification^34,35^, the most common approach to develop bioorthogonal reporters of monosaccharides is the replacement of hydroxyl groups with azido groups^36–38^. Previous studies developed a 4-azido-substituted Xyl analog that is incorporated into proteoglycans by XT1 (ref. ^39^) but the corresponding UDP-sugar cannot be biosynthesized in mammalian cells as there is no salvage pathway for Xyl^40^. The BH tactic uses substrate analogs that would normally not be accepted by glycosyltransferases (GTs)^41–43^. Thus, we sought to reprogram XTs to accept a UDP-sugar that is not accepted by WT-XT1 but can be biosynthesized in mammalian cells. WT-XT1 has been reported to use UDP-6-azido-6-deoxy-D-glucose (UDP-6AzGlc) with approximately 20-fold lower enzymatic efficiency than UDP-xylose (UDP-Xyl)^44^. We opted to develop a mutant with reversed selectivity to accept UDP-6AzGlc over UDP-Xyl. As an analog of Glc, 6AzGlc was projected to hijack parts of the UDP-Glc salvage pathway and, therefore, allow cellular biosynthesis unlike Xyl analogs^36^.

Our recent crystal structure of XT1 revealed a two-lobe architecture containing a catalytic glycosyltransferase (GT) domain^17^. As XT1 contains an unusually constricted UDP-Xylbinding site that prevents the use of larger UDP-sugars such as UDP-6AzGlc, we deemed it possible to generate additional space (a ‘hole’) in the active site by mutation. XT1 harbors several bulky ‘gatekeeper’ amino acids in close proximity to C5 of UDP-Xyl, namely Trp392, Leu525 and Leu526 (Fig. 1b). We designed, expressed and purified from Expi293 cells a total of 16 XT1 single-mutant and double-mutant variants in which these residues were replaced with smaller amino acids (Fig. 1c and Supplementary Fig. 1). In vitro glycosylation of a bikunin substrate peptide in a high-performance liquid chromatography (HPLC) assay served to assess glycosylation from the sugar donors UDP-Xyl, UDP-6AzGlc and, as a substrate of intermediate size of the ‘bump’, UDP-Glc^17^.

WT-XT1 displayed exclusive activity for UDP-Xyl in our hands, with no activity toward UDP-Glc or UDP-6AzGlc^44^. Most engineered XT1 variants displayed either no activity at all or were still selective for UDP-Xyl, with some displaying activity toward UDP-Glc. Strikingly, the variant Leu526Gly preferred UDP-6AzGlc as a substrate, with 7–8-fold higher turnover than using UDP-Xyl or UDP-Glc in an endpoint assay. Compared to the Leu526Gly mutant (henceforth termed ‘BH-XT1’), the construct Leu526Ala displayed no such selectivity, with equal activity on all three UDP-sugars (Fig. 1c). We determined optimal enzyme concentrations and kinetic constants for the native and BH enzyme–substrate pairs (Fig. 1d and Supplementary Figs. 2 and 3). We found that the *K*_M_ of the BH pair (41.2 µM) was similar to the WT pair (37.4 µM), while the *k*_cat_ was 2.8-fold reduced in the BH pair. In contrast, BH-XT1 uses UDP-Xyl with an approximately tenfold lower catalytic efficiency and UDP-Glc with an approximately threefold lower catalytic efficiency than UDP-6AzGlc, suggesting that the native UDP-sugar substrates should not be able to outcompete UDP-6AzGlc in cellular applications. Taken together, we established a sensitive structure–activity relationship in the development of a BH-XT1 variant.

### XT engineering retains the peptide specificity

**Fig. 2:**
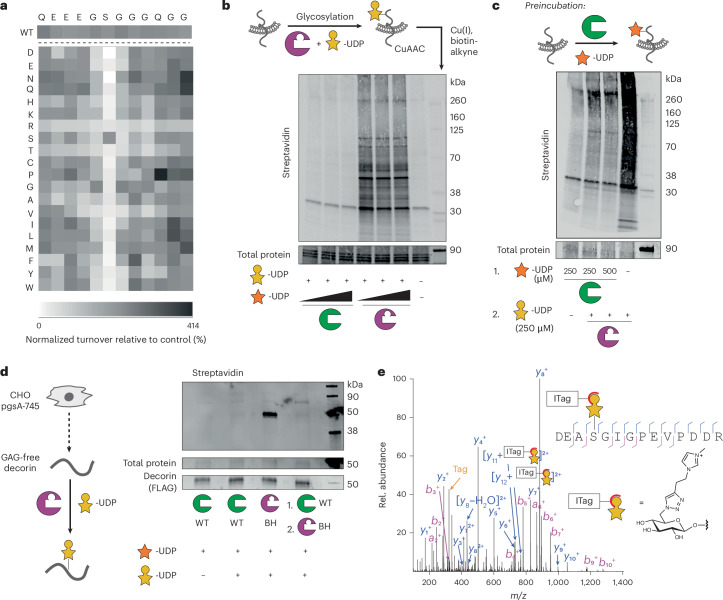
BH engineering preserves protein substrate specificity of XT1. **a**, In vitro glycosylation of a peptide substrate panel based on the bikunin peptide indicated at the top as assessed by a luminescence assay, with every position substituted for each of the 20 amino acids. The intensity of the grayscale indicates the percentage turnover normalized to WT bikunin peptide. The WT bikunin peptide was present 12 times in the panel (copied in the top row) and all other peptide reactions were normalized on the average of these 12 data points. Data are from one of *n* = 3 independent experiments. **b**, In vitro glycosylation of a membrane protein fraction of XT2-KO CHO^KO *Xyl2t*^ cells as assessed by streptavidin blot. Reactions contained 250 µM UDP-6AzGlc and 100, 200 or 300 µM UDP-Xyl and were reacted with biotin-alkyne before blotting. Data are from one of *n* = 2 independent experiments. **c**, Glycosylation by BH-XT1–UDP-6AzGlc can be prevented by preincubation with WT-XT1–UDP-Xyl. Reactions were processed as in **b**. Data are from one experiment. **d**, In vitro glycosylation of a GAG-free preparation of human decorin purified from pgsA-745 CHO cells as assessed by streptavidin blot. Reactions were run with 250 µM UDP-sugars and processed as in **b**,**c**. Data are from one of *n* = 2 independent experiments. **e**, Analysis of the glycosylation site on decorin introduced by BH-XT1 by MS and HCD fragmentation. Decorin was in vitro glycosylated as in **c**, subjected to CuAAC with ITag-alkyne^45^, digested and subjected to MS-glycoproteomics. Fragments are annotated on the tryptic peptide from mature decorin. ‘Tag’ denotes the signature ion 322.1508 *m*/*z* from the depicted chemically modified sugar. Data are from one experiment. Source data

To assess whether BH engineering retains substrate preference of WT-XT1 toward the proteoglycan backbone, we first tested the BH enzyme–substrate pair (BH-XT1 and UDP-6AzGlc) with a panel of 240 acceptor peptides in an in vitro glycosylation assay. The panel contained derivatives of the bikunin XT1 substrate peptide in which each amino acid was substituted with each of the other 19 proteinogenic amino acids. We previously used the same peptide panel to extract amino acid preferences of the native enzyme–substrate pair (WT-XT1 and UDP-Xyl) in a luminescence-based assay^17^. Using the same assay, the peptide substrate preferences were found to be conserved between WT-XT1 and BH-XT1, with positive Pearson correlation across all peptides and specifically to individual substitutions across all independent replicates (Fig. 2a and Supplementary Figs. 4 and 5; WT peptide preference discussed in a previous study^17^). Introducing basic Lys or Arg residues anywhere in the substrate peptide lowered enzyme activity, whereas acidic Glu and Asp tended to increase activity. An exception was the substitution of Glu at the −4 position with Asp that led to a decrease in turnover for both WT-XT1 and BH-XT1 (ref. ^17^). Substitutions of glycine residues at positions −1 and +1 of the central Ser were not well tolerated by either WT-XT1 or BH-XT1. An exception was substitution of Gly at +1 position to hydrophobic amino acids Leu, Met or Phe, which led to residual activity in BH-XT1 but not WT-XT1. As this Gly is in contact with Leu526 in WT-XT1, we reasoned that the Leu526Gly ‘hole’ left space for substitutions to larger hydrophobic amino acids in substrate peptides. As these were the only reproducible differences between WT-XT1 and BH-XT1 (other replicates in Supplementary Fig. 4) and low in number from a 240-member peptide library, we concluded that BH engineering exhibits conservation of peptide substrate preference in vitro.

### BH-XT1 glycosylates proteoglycans at GAG attachment sites in vitro

We next assessed whether BH-XT1 retains the activity of WT-XT1 to initiate GAG attachment sites on proteoglycan backbones. We prepared membrane fractions from Chinese hamster ovary (CHO) cells with or without KO for endogenous xylosyltransferase genes *Xylt1* and *Xylt2* (ref. ^10^). Membrane fractions were incubated with recombinant WT-XT1 or BH-XT1 and synthetic UDP-Xyl and UDP-6AzGlc, followed by reaction with alkyne-biotin under copper-catalyzed azide–alkyne cycloaddtion (CuAAC) ‘click’ conditions. Analysis by streptavidin blot suggested labeling of lysate proteins with 6AzGlc only when BH-XT1 but not WT-XT1 was present (Supplementary Fig. 6). As *Xylt2* is the major xylosyltransferase gene expressed in CHO cells^10^, we used CHO^KO *Xylt2*^ cells for further in vitro glycosylation experiments. We established that labeling by BH-XT1 with UDP-6AzGlc could not be outcompeted with increasing concentrations of UDP-Xyl, suggesting that BH-XT1 specifically and potently recognizes UDP-6AzGlc as a substrate (Fig. 2b). Preincubation of the membrane protein fraction with WT-XT1 and UDP-Xyl abrogated incorporation of 6AzGlc by BH-XT1, suggesting that the same glycosylation sites are introduced by both enzymes (Fig. 2c).

We next confirmed in vitro that BH-XT1 emulates the activity of WT-XT1 to glycosylate proteoglycans. Human decorin has a single site of GAG attachment. Recombinant expression in the CHO cell mutant pgsA-745 that lacks endogenous XT activity results in a GAG-free decorin preparation^7,8^. We incubated this GAG-free decorin with either WT-XT1 or BH-XT1 in the presence of UDP-Xyl and/or UDP-6AzGlc, followed by CuAAC with alkyne-biotin and streptavidin blot (Fig. 2d). While WT-XT1 activity did not lead to discernible streptavidin signal on decorin, BH-XT1 in the presence of UDP-6AzGlc led to an intense streptavidin signal that could be abrogated by preincubation of decorin with WT-XT1 and UDP-Xyl. These data indicate that the single GAG attachment site was blocked with a Xyl residue by WT-XT1, preventing BH-XT1 activity. We observed the same behavior in a GAG-free preparation of human glypican 1, GPC1 (Supplementary Fig. 7), suggesting that BH-XT1 recapitulates the activity of WT-XT1 across a range of proteoglycans.

We confirmed the glycosylation site modified by BH-XT1 on in vitro glycosylated recombinant decorin by tandem MS. Two fragmentation methods are routinely used for *O*-glycopeptides. Higher-energy collision-induced dissociation (HCD) primarily fragments the glycosidic bond to detect glycan oxonium ions while electron transfer dissociation (ETD) fragments the peptide backbone to allow glycan site annotation. The clickable azide tag was essential to improve sugar identification in mass spectra, allowing incorporation of functional groups that are beneficial to analysis. Specifically, we used a clickable imidazolium tag (ITag) that carries a permanent positive charge and increased the charge state of glycopeptides, allowing direct glycosylation site annotation^45^. We first applied a standard workflow in which HCD fragmentation led to an ITag-containing, 6AzGlc-derived signature ion that was used to trigger ETD on the same glycopeptide^35,41,43,45^. This tandem strategy is used because *O*-linked glycan modifications are usually too labile to be detected within peptide fragments during HCD, hampering glycosylation site localization. Surprisingly, ITag-modified 6AzGlc was detected on peptide fragments in HCD spectra on a tryptic glycopeptide derived from decorin, without the need for additional ETD (Fig. 2e).

Decorin is proteolytically processed during secretion to remove a propeptide and shorten the N terminus^46^. HCD fragmentation allowed for direct identification of Ser34 as the attachment site of 6AzGlc by BH-XT1 on this mature form, consistent with Ser34 being the site of cellular GAG attachment (Fig. 2e)^47^. Taken together, these results suggest that the BH-XT1 enzyme–substrate pair glycosylates native GAG attachment sites in proteoglycans in vitro.

### Biosynthesis of UDP-6AzGlc through a caged sugar-1-phosphate

**Fig. 3:**
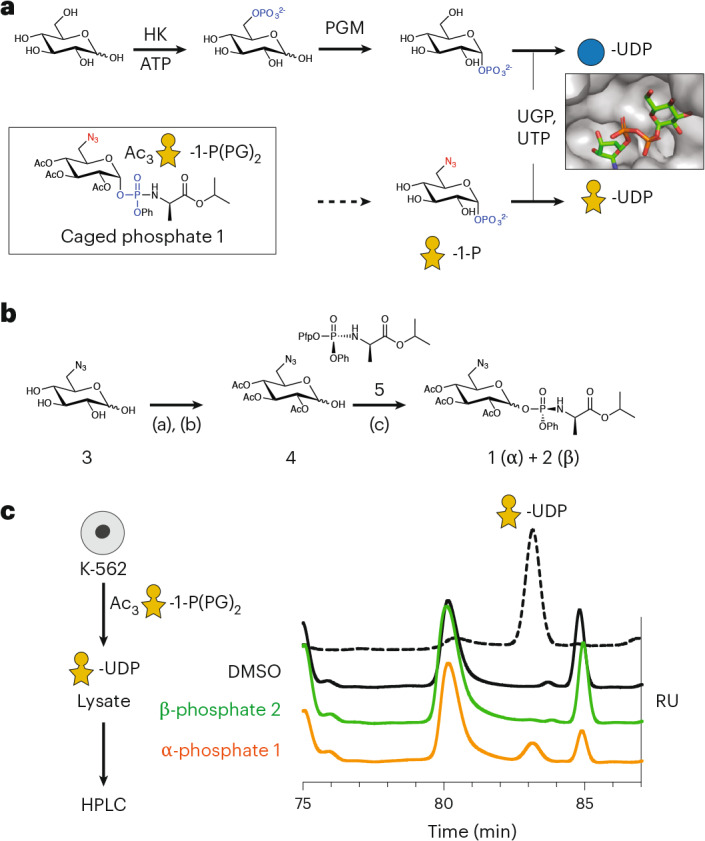
Biosynthesis of UDP-6AzGlc. **a**, Biosynthetic pathway of UDP-Glc through hexokinase (HK), PGM and UGP. Biosynthesis of UDP-6AzGlc from caged phosphate **1** bypasses the HK and PGM steps. Insert, crystal structure of UGP with UDP-Glc indicating that the 6-OH group protrudes into an open cavity (PDB 4R7P). **b**, Synthesis of caged sugar-1-phosphates **1** and **2** from 6AzGlc **3** through triacetylated intermediate **4**, followed by treatment with phosphoramidite precursor **5**. **c**, Biosynthesis of UDP-6AzGlc in K-562 cells as assessed by ion-exchange HPLC of lysates fed with compounds **1** or **2**. Data are from one of *n* = 2 independent experiments. The dotted line represents an authentic standard of UDP-6AzGlc. Peaks at 80 and 85 min represent unspecified contents in cellular lysates. Reagents and conditions of reactions in **b**: (a) acetic anhydride, pyridine, dimethylaminopyridine, room temperature, 90%; (b) acetic acid, ethylene diamine, room temperature, 45–73%; (c) 2 M lithium diisopropylamide in tetrahydrofuran, **5**, −78 °C to −70 °C, (**1**) 60%, (**2**) 9.8%. RU, relative units. Source data

Application of a GT BH system in living cells requires biosynthesis of the nucleotide-sugar. In general, caged, membrane-permeable monosaccharide precursors are used with ester modifications that are deprotected in the cytosol. Free monosaccharides can then be converted to UDP-sugars before transport to the Golgi^42,43,48^. Although human cells are devoid of a salvage pathway for UDP-Xyl, the use of UDP-6AzGlc provided an opportunity for the cellular biosynthetic pathway for UDP-Glc instead. Glc is activated in mammalian cells first by phosphorylation to Glc-6-phosphate and, subsequently, isomerization by phosphoglucomutase (PGM) to Glc-1-phosphate (Fig. 3a). Conversion to UDP-Glc then features the enzymes UDP-Glc pyrophosphorylase 1 or 2 (UGP1/2). As phosphorylation at the 6-position was prevented by the azido group, we sought to bypass the kinase and PGM steps and provide a sugar-1-phosphate as a direct substrate for UGP1/2. We were encouraged by analysis of the UGP1–UDP-Glc cocrystal structure in which the UDP-Glc 6-hydroxyl group is solvent exposed, suggesting that an azido group at that position should be tolerated by the enzyme (Fig. 3a)^49^. While we and others have made sugar-1-phosphates caged as labile bis-*S*-acetylthioethyl (SATE) phosphotriesters, synthesis of SATE-caged 6AzGlc-1-phosphate failed in our hands^43,50,51^. Instead, we took inspiration from the increasingly popular ProTide technology that has gained attention to cage phosphates in antiviral nucleotides^52,53^ and has been recently used to cage sugar-phosphates^54–56^. We synthesized phosphoramidate diester **1** as a caged sugar-1-phosphate to be deprotected by hydrolases in the cytosol of living mammalian cells (Fig. 3a and Supplementary Note)^52^. The synthesis proceeded from 6-azido-6-deoxy-D-Glc **3** through the intermediate triacetate **4**. Treatment of **4** with phosphoramidite precursor **5** under basic conditions yielded both α-phosphoramidate diester **1** (60% yield) and β-phosphoramidate diester **2** (9.8% yield) (Fig. 3b)^57^. As UGP1/2 is naturally restricted to the α-configured Glc-1-phosphate, preferential formation of the α-anomer was gratifying. In turn, β-phosphoramidate diester **2** served as a negative control in feeding experiments. Feeding K-562 cells the α-phosphoramidate diester **1** led to notable and reproducible biosynthesis of UDP-6AzGlc (Fig. 3c). In turn, β-configured phosphoramidate diester **2** led to negligible UDP-6AzGlc levels, possibly arising from very small (4%) amounts of α-configured **1** as a contaminant of **2** (Supplementary Information). These data suggest that α-phosphoramidate diester **1** is a suitable precursor to deliver UDP-6AzGlc to mammalian cells by entering the UDP-Glc biosynthetic pathway.

### Development of a cellular BH-XT1 system

**Fig. 4:**
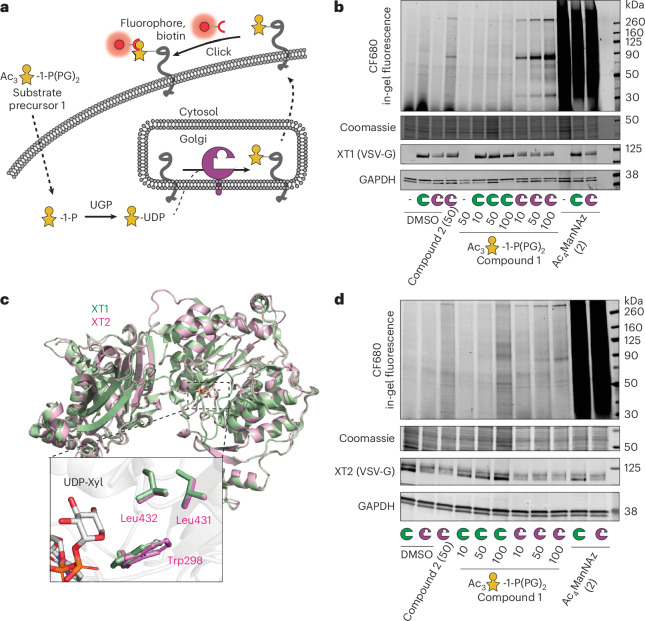
BH-engineered xylosyltransferases label glycoproteins in mammalian cells. **a**, Overview of cellular BH engineering. UDP-6AzGlc is biosynthesized after delivery through compound **1** and used by BH-engineered XTs. Cell-surface CuAAC detects proteoglycans. **b**, Chemical tagging of proteoglycans by BH-XT1 on pgsA-745 CHO cells as assessed by in-gel fluorescence. Cells stably expressing WT-XT1 or BH-XT1 or nontransfected were fed with compounds in the indicated concentrations in µM before on-cell CuAAC and in-gel fluorescence. Western blots are from a separate gel using the same samples. Data are from one of *n* = 2 independent replicates. **c**, Structural alignment between the crystal structure of human XT1 (PDB 6EJ7, green) and the AlphaFold structure of human XT2 (UniProt Q9H1B5, purple) with aligned gatekeeper residues in the insert. **d**, Chemical tagging of proteoglycans by BH-XT2 on pgsA-745 CHO cells as assessed by in-gel fluorescence. Cells stably expressing WT-XT2 or BH-XT2 were fed and treated as in **b**. Data are from one of *n* = 2 independent experiments. Source data

With a strategy for UDP-6AzGlc delivery in hand, we established a BH system to chemically tag XT1 substrate proteins in mammalian cells (Fig. 4a). The pgsA-745 CHO cell line was stably transfected with plasmids encoding WT-XT1 or BH-XT1 using a transposase-based genome insertion method^43,58^, followed by feeding the 6AzGlc-1-phosphate precursor **1**. After overnight incubation, CuAAC was performed to attach clickable alkyne-CF680 to 6AzGlc on the cell surface while keeping cells alive^35,43,48^. Surplus click reagents were washed away, cells were lysed and fluorophore incorporation was assessed by SDS–PAGE and in-gel fluorescence (Fig. 4b). Minimal background fluorescence was observed in cells fed with DMSO or only expressing WT-XT1, even when fed with increasing concentrations of 6AzGlc-1-phosphate precursor **1**. In the presence of BH-XT1, clear bands of fluorescently labeled proteins were observed at 30 kDa, 90 kDa and >260 kDa. With increasing feeding concentration of **1**, a concentration-dependent increase of fluorescence was observed, along with labeled protein bands of lower intensity, especially between 50 and 90 kDa. In accordance with biosynthetic experiments, the β-configured 6AzGlc-1-phosphate **2** yielded a weak and diffuse labeling signal when fed to cells, indicating that UDP-6AzGlc biosynthesis is a direct prerequisite for cellular chemical tagging of glycoproteins by BH-XT1. The fluorescence bands at 90 kDa and 260 kDa in the BH-XT1–compound **1** lanes were observed weakly when compound **2** was fed, which we attribute to small residual levels of compound **1** in the preparation (Fig. 3c). Ac_4_ManNAz as a precursor to azide-tagged sialic acid yielded a strong and uniform fluorescence signal across all cell lines tested^59^. Our data indicate that BH-XT1 specifically tags cell-surface proteins with bioorthogonal 6AzGlc in the secretory pathway of mammalian cells.

The mammalian genome encodes two xylosyltransferase isoenzymes that have been differentially implicated in disease and proteoglycan function^28,30,32,60^. To allow for substrate profiling of the second isoenzyme XT2, we extended the BH strategy by using structural similarities between the two isoenzymes. A structural overlay between the XT1 crystal structure and the XT2 AlphaFold structure highlighted conservation of amino acids interacting with UDP-Xyl (Fig. 4c). We identified the gatekeeper residues Leu431, Leu432 and Trp298 in XT2 that occupied the same role as the respective residues in XT1, with Leu432 in XT2 overlaying with Leu526 in XT1. The BH-XT2 mutant L432G was, thus, stably expressed in pgsA-745 CHO cells. Feeding with 6AzGlc-1-phosphate precursor **1** and cell-surface CuAAC reaction with CF680-alkyne led to a similar band pattern by in-gel fluorescence, with glycoprotein bands at 90 kDa and >260 kDa identified in BH-XT2-expressing cells in a concentration-dependent manner (Fig. 4d). We noted that the intensity of fluorescence bands specifically tagged by BH-XT2 was lower than by BH-XT1, indicating a lower activity of the second isoenzyme. WT-XT2 expression did not lead to the same fluorescence band pattern. Neither feeding DMSO nor compound **2** led to discernible signal over background and Ac_4_ManNAz was included as a positive labeling control. To apply the BH-XT approach in human cells, we knocked out the *XYLT1* or *XYLT2* gene in the K-562 leukemia cell line. Cells were transfected with plasmids encoding for the corresponding WT-XT or BH-XT and biorthogonal tagging was performed through feeding **1**. A cell-surface CuAAC reaction with CF680-alkyne led to a discernible glycoprotein band pattern by in-gel fluorescence, with bands at 70 kDa and 160 kDa identified in both BH-XT1-expressing and BH-XT2-expressing cells in a concentration-dependent manner (Supplementary Fig. 8). Our data suggest that the BH approach is applicable to both xylosyltransferase isoenzymes in mammalian cells, allowing to assess their substrate profiles.

### XT engineering enables profiling of cellular proteoglycans

**Fig. 5:**
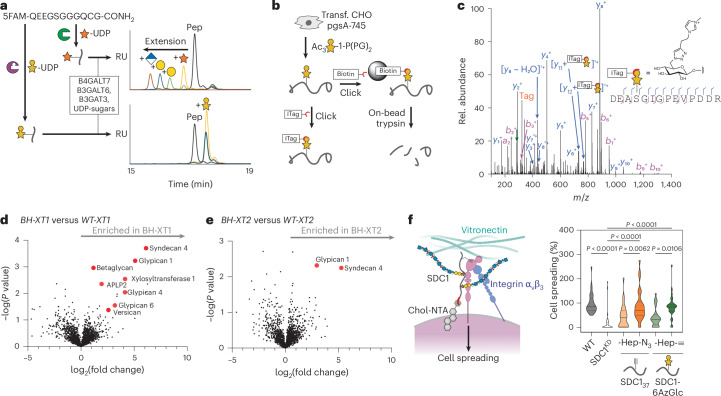
Chemical functionalization by BH-XT1/2 enables detection and manipulation of proteoglycans. **a**, 6AzGlc is not elongated by GAG linker enzymes. Fluorescently labeled bikunin-derived peptide was incubated with WT-XT1–UDP-Xyl or BH-XT1–UDP-6AzGlc and, subsequently, with the indicated soluble GTs^7^ and corresponding UDP-sugars. Elongation was assessed on each step by HPLC. Data are from one experiment each with different combinations of transferases. **b**, Using BH-XT1/2 to profile proteoglycans by MS. **c**, Analysis of the glycosylation site on decorin introduced by BH-XT1 in living cells by MS and HCD fragmentation. Decorin was coexpressed with BH-XT1 in cells fed with **1**, then subjected to CuAAC with ITag-alkyne^45^, digested and subjected to tandem MS. Fragments are annotated on the tryptic peptide from mature decorin. Not all fragments are shown. ‘Tag’ denotes the signature ion 322.1508 *m*/*z* from chemically modified sugar. Data are from one experiment. **d**, Differential MS experiment of protein fractions enriched in BH-XT1-expressing versus WT-XT1-expressing pgsA-745 CHO cells fed with compound **1** (250 µM) from *n* = 3 independent replicates. The proteoglycans were assigned and processed from a *C*. *griseus* protein database search (UniProt) followed by visualization of data on Perseus. **e**, Differential MS experiment of protein fractions enriched in BH-XT2-expressing versus WT-XT2-expressing pgsA-745 CHO cells fed with compound **1** (250 µM) from *n* = 3 independent replicates. In **d**,**e**, statistical significance was assessed using a two-sample Welch’s *t*-test to evaluate differential enrichment between BH/WT-XT1/XT2 pairs and generate *P* values. Multiple-hypothesis testing was corrected using the Benjamini–Hochberg procedure to calculate FDRs. Protein hits with adjusted FDR values ≤ 0.05 were retained for visualization. **f**, Analysis of cell spreading of MDA-MB-231 cells (dark gray) with SDC1-knockdown (SDC1^KD^, light gray) and rescue with clickable heparin introduced either into alkyne-tagged SDC1_37_ or azide-tagged SDC1-6AzGlc (light orange for SDC1_37_, dark orange for SDC_37_-Hep, light green for SDC1-6AzGlc and dark green for SDC1-6AzGlc-Hep). Spreading relies on the interaction between HS/heparin-containing SDC1 and α_V_β_3_ integrin on MDA-MB-231 cells and vitronectin on coated wells. Data are truncated violin plots of cell counts in at least *n* = 6 different frames per replicate in *n* = 3 independent replicates. Lines depict the median and quartiles. Statistical significance was assessed using a one-way analysis of variance with Tukey’s post hoc test. Source data

Xylosyltransferase BH engineering is poised to allow the identification of proteoglycans, a feat that normally requires elaborate methods of glycopeptide enrichment and characterization^9,21,23^. A prerequisite to glycoprotein enrichment and identification is biosynthetic simplicity; ideally, 6AzGlc would replace an entire GAG chain without added complexity for glycan elaboration. Thus, to establish an MS-glycoproteomics workflow, it was important to assess whether 6AzGlc, like Xyl, was extended to a functional GAG linker tetrasaccharide. We recently reported an enzymatic method for extension of xylosylated glycopeptides by recombinant preparations of the GTs: B4GALT7, B3GALT6 and B3GAT3 (termed linker enzymes) in the presence of UDP-galactose (UDP-Gal) and UDP-glucuronic acid (UDP-GlcA). Using a fluorescently labeled bikunin-derived peptide, we first confirmed by HPLC that attachment of either Xyl (by WT-XT1) or 6AzGlc (by BH-XT1) led to a shift of peptide retention time (Fig. 5a). Upon addition of the linker enzymes, the Xyl moiety was sequentially elongated to the full tetrasaccharide. In contrast, a 6AzGlc-modified peptide did not shift in retention time upon incubation with the GTs and UDP-sugars. We concluded that 6AzGlc is a chain-terminating modification that is not extended to functional GAG chains. We interpreted this substantial decrease in glycan complexity as an advantage for MS and to manipulate the composition of proteoglycans, as discussed below.

We next determined that a functional XT1 system chemically tags a model proteoglycan in mammalian cells by MS. FLAG-tagged human decorin was overexpressed as a secreted proteoglycan in pgsA-745 CHO cells that expressed BH-XT1. Cells were fed with 6AzGlc-1-phosphate precursor **1** and decorin was immunoprecipitated from conditioned supernatant. The preparation was subjected to CuAAC with ITag-alkyne, digested and subjected to MS with HCD fragmentation. We confirmed unambiguously that Ser34 was glycosylated by BH-XT1 inside mammalian cells, confirming the BH approach as suitable to identify proteoglycans including native Xyl attachment sites (Fig. 5b,c).

Our in-gel fluorescence data suggested that the two isoenzymes XT1 and XT2 may exhibit an overlapping substrate glycoprotein profile. To test this notion, we compared the glycoproteome tagged by either BH-XT1 or BH-XT2 in cells by MS. Chemically tagged glycoproteins in conditioned supernatants of stably transfected pgsA-745 CHO cells fed with 250 µM caged phosphate **1** were treated with alkyne-biotin under CuAAC conditions, enriched on neutravidin resin and subjected to on-bead proteolytic digest. Peptide fractions were analyzed by MS using data-independent acquisition over three replicates each, with WT-XT1/2-expressing cells fed with 250 µM caged phosphate **1** as control conditions. Data were processed using the software DIA-NN to identify and quantify *Cricetulus* *griseus* proteins^61^. Enriched peptide and protein fractions were analyzed with Perseus^62^. Glycosylation by BH-XT1 led to a striking enrichment of cellular proteoglycans, including syndecan 4 (SDC4), glypicans (GPCs) 1, 4 and 6, APLP2 (amyloid precursor-like protein 2), versican, betaglycan and CD44, compared to the supernatant of cells expressing WT-XT1 (Fig. 5d and Supplementary Fig. 9). To control for the relatively high concentration of compound **1** used in these experiments, we repeated the experiment with 50 µM **1** over four independent replicates (Supplementary Fig. 10). Similarly, glycosylation by BH-XT1 led to the enrichment of bona fide proteoglycans decorin, SDC4, GPC1 and CD44. We note that these data may explain the band pattern seen by in-gel fluorescence (Fig. 4b), with tentative annotations of SDC4 (30 kDa), glypicans (50–70 kDa), APLP2, betaglycan or CD44 (90 kDa) and versican (>260 kDa). BH-XT2 led to strong and selective enrichment of GPC1 and SDC4 over WT-XT2 at both concentrations tested (Fig. 5e and Supplementary Figs. 9 and 10). In both cases, we analyzed the identity of detected peptides from these enriched proteins and found them to lie outside previously annotated glycosylation site(s) (Supplementary Fig. 11 and Supplementary Table 1). These findings suggest that cellular BH-XT1/2 engineering allows unambiguous proteoglycan profiling without the need to annotate the GAG attachment site. When a less stringent comparison was made between cells expressing BH-XT1/2 fed with compound **1** against the same cell lines fed with DMSO as a control, the bona fide proteoglycans nidogen 1, CS proteoglycan 4, agrin and aggrecan were additionally found to be enriched (Supplementary Fig. 12). We conclude that BH engineering enables straightforward detectability of proteoglycans from mammalian cells.

### Functional annotation of the GAG modification on proteoglycans

The BH approach replaces the GAG chain of a proteoglycan with a bioorthogonal modification. We reasoned that this approach could be used to introduce suitably modified GAG polysaccharides, creating neoglycoproteins that allow for functional dissection of the proteoglycan components in cell biology. We previously used unnatural amino acid introduction through amber stop-codon reassignment to produce SDC1 chemically tagged at position 37 (termed SDC1_37_) with a clickable alkyne^16^. Through CuAAC, SDC1_37_ could be furnished with azide-containing, clickable heparin to generate a neoglycoprotein termed SDC1_37_-Hep. In the presence of either fully glycosylated, HS-containing SDC1 or SDC1_37_-Hep, MDA-MB-231 cells exhibit enhanced spreading on vitronectin-coated surfaces in an integrin α_v_β_3_-dependent fashion that can be analyzed by microscopy. Soluble SDC1 preparations can be deposited on cells by virtue of their 6×His tags through the use of a synthetic Ni^2+^-displaying, membrane-anchored cholesterol anchor^16^. We used BH-XT1 to introduce 6AzGlc into a nonglycosylated WT-SDC1 preparation in vitro, to generate SDC1-6AzGlc. Heparin was derivatized with an anomeric alkyne tag through hydrazide chemistry and introduced into 6AzGlc-SDC1 through CuAAC to furnish SDC1-6AzGlc-Hep.

Both SDC1-Hep conjugates displayed an increase in molecular weight (Supplementary Fig. 13). MDA-MB-231 cells were depleted of endogenous SDC1 by siRNA-based knockdown, significantly impacting cell spreading (Fig. 5f). Deposition of nonglycosylated SDC1_37_ or SDC1-6AzGlc enhanced cell spreading by approximately 20%, suggesting a role of the SDC backbone alone for MDA-MB-231 spreading that has been noted before^16^. Heparin-containing SDC1_37_-Hep and SDC1-6AzGlc-Hep fully rescued cell spreading, indicating that BH engineering and bioorthogonal chemistry restored the functional properties of a proteoglycan.

## Discussion

The importance of proteoglycans in physiology is undisputed, as the vast majority of signaling events between cells or with the extracellular matrix are modulated by the associated GAG chains. While great efforts are being made to understand the details of GAG polysaccharide sequence on biology^10,14,22,63,64^, we still lack important information on the first step of glycosylation to the protein backbone. The two human xylosyltransferases display tissue selectivity and differences in attached GAG sequences but we do not yet have fundamental insight into their individual biological functions^28,31–33,60^. A chemical tool to dissect XT1/2 biology must accurately report on XT1/2 activity while being orthogonal to other glycosylation events in the secretory pathway and deliverable to living mammalian cells. Both catalytic efficiency and peptide substrate preference of the BH-XT1 enzyme–substrate pair were remarkably conserved. Incorporation of 6AzGlc into cell-surface proteins was dependent on the presence of BH-XT1. We note that this finding does not exclude incorporation into glycoconjugates that naturally contain D-Glc, as most of these are either glycolipids or intracellular glycoproteins, neither of which would be visible with our assays.

The finding that 6AzGlc is not extended to the common GAG linker tetrasaccharide was expected because of the restrictive active site architecture of the follow-on enzyme B4GALT7 (ref. ^65^). However, we recognize this nonextension as an advantage for MS because 6AzGlc is structurally well defined and can be directly modified through bioorthogonal chemistry. In our MS experiments, annotation was further simplified by the availability of the ITag technology to facilitate MS^45,66^. Furthermore, a chain-terminating, clickable inhibitor of chain extension has the potential to be used to study GAG biology in vitro or in vivo^16,39,67^, substantially expanding our toolbox.

Establishing a cellular XT BH system required a biosynthetic entry point for UDP-6AzGlc. Previously used per-acetylated 6AzGlc was not a suitable precursor for glycosylation in our hands^36^ but we note that cell lines from different organisms can vary in their biosynthetic potential^68^. Nevertheless, a ProTide-based caged sugar-1-phosphate was a reliable precursor for UDP-6AzGlc to fashion a cellular BH system.

While XT2 appears to be the dominant isoenzyme expressed in humans, dysfunctions in both enzymes lead to severe yet differential disorders in mouse models and in humans^30–33^. After fully characterizing a BH-XT1 system, we designed a functional BH-XT2 mutant simply on the basis of structural homology. BH-XT2 was directly applied in living cells without characterizing the corresponding soluble recombinant enzyme first, showcasing the reliability of the tactic and the importance of structural data for identification of nucleotide-sugar binding.

Proteoglycans can be perceived as a modular assembly between a protein backbone and one or more GAG chains. While biological function can be imparted by either component, it can be challenging to differentiate both. The biosynthetic details of GAG extension by either HS or CS/DS are beginning to be understood^7^ but methods to reliably swap the GAG chain on a given proteoglycan are of fundamental importance for proteoglycan biology. We recently used stop-codon reassignment to introduce an alkyne-tagged amino acid into a recombinant proteoglycan backbone^16^, as a critical aspect of understanding the role of SDC1 for cell spreading. We applied BH engineering to underpin these findings, allowing to chemoenzymatically attach a chemical modification to the recombinant protein. We note the ability to use this modular approach to generate ‘designer proteoglycans’ with unnatural GAG chains to generate functional understanding.

Our work is setting the foundation to establish the fine differences between XT1 and XT2 and profile proteoglycans in a range of different model systems.

## Methods

### Cloning of XT1 mutants for in vitro assays

A pCEP-Pu vector containing DNA coding residues 232–959 of WT and Trp392Ala human xylosyltransferase 1 (original complementary DNA from Dharmacon, BC045778, clone 4791553) with a tobacco etch virus protease-cleavable His tag at the N terminus of the secreted protein was used previously^17^. The plasmid was used as a template to generate genes of single and double mutants of XT1 by overlap extension PCR using the Q5 HiFi polymerase (New England Biolabs) and bespoke primers (Supplementary Table 1) designed with the Agilent QuikChange tool (https://www.agilent.com/store/primerDesignProgram.jsp, accessed on 4 December 2023), according to the manufacturer’s instructions. In PCR step 1, two 25-µl reactions were set up individually with 10 ng of WT-XT1 template DNA, 12.5 µl of Q5 HiFi mastermix and either 0.5 µM primer mix of ‘XT1 pOPING for’ or ‘XT1 pOPING rev’ primer with ‘XT1 pOPING infusion rev’ or ‘XT1 pOPING infusion for’ primer for the targeted XT1 mutation. Conditions featured 1 min at 98 °C (denaturation), 25 cycles of 10 s at 98 °C, 30 s at 68 °C and 1 min at 72 °C and a final extension of 15 min at 72 °C. PCR products from the two reactions were purified by agarose gel using a Macherey-Nagel NucleoSpin gel and PCR cleanup kit (Thermo Fisher Scientific), concentration was measured by nanodrop ranging from 10 to 50 ng µl^−1^ and then 2.5 µl of the purified PCR products were mixed with 0.5 µM primer mix of infusion for and infusion rev primers following the same PCR protocols as above. Next, 100 ng of purified final PCR product was then mixed with 50 ng pOPING plasmid digested with KpnI-HF and Pmel (New England Biolabs). pOPING plasmid was a gift from R. Owens (Addgene, plasmid 26046; RRID:Addgene_26046)^69^. Inserts were introduced using an infusion HD cloning kit (Takara Bio) according to the manufacturer’s instructions. Stellar competent cells (Takara Bio) were transformed by heat shock and used for plasmid amplification. All plasmids were confirmed by Sanger sequencing (Genewiz) and nanopore sequencing (Plasmidsaurus) before use.

### Expression and enrichment of XT1 mutants

Expi293F cells (Thermo Fisher Scientific) were cultured in 10 ml of Gibco FreeStyle medium (Thermo Fisher Scientific) in 50-ml cell culture flasks at 37 °C, 8% CO_2_ and 125 rpm. The cells were subcultured to 0.8 × 10^6^ cells per ml 2 days before transfection at the exponential growth phase with a viability of at least 95%. Transfection of the Expi293F cells was performed at a density of 3 × 10^6^ cells per ml using the ExpiFectamine 293 transfection kit (Thermo Fisher Scientific). For each 10-ml cell culture, 10 µg of DNA and 30 µl of ExpiFectamine 293 were diluted in 500 µl of OptiMEM I (Thermo Fisher Scientific), with the ratio of DNA to ExpiFectamine 293 at 1:3 (w/v) and incubated at room temperature for 5 min. They were then mixed and incubated for 20 min at room temperature to allow the DNA–transfection reagent complexes to form before being added drop by drop to the cells. The cells were then incubated in the same conditions for another 20–24 h before adding 50 µl of ExpiFectamine 293 transfection enhancer 1 and 500 µL of transfection enhancer 2. The cell culture supernatants were harvested on the fifth day in 15-ml tubes by centrifuging at 500*g* for 5 min and then 100× Halt protease inhibitor cocktail (Thermo Fisher Scientific) was added to a 1× final concentration.

HisPur Ni-NTA RA resin (Thermo Fisher Scientific) (100 µl of bead slurry per 10 ml cell culture medium volume) were washed with ten column volumes (CVs) of water and equilibration buffer (25 mM Tris-HCl pH 7.5, 150 mM NaCl and 20 mM imidazole) twice each before being added to the cell culture supernatant and incubated at 4 °C on a roller overnight. The cell culture supernatants were then centrifuged at 500*g* for 10 min at 4 °C. The resulting resin was resuspended and eluted sequentially with ten CVs of equilibration buffer, ten CVs of 25 mM Tris-HCl pH 7.5 with 150 mM NaCl and 50 mM imidazole and ten CVs of 25 mM Tris-HCl pH 7.5 with 150 mM NaCl and 200 mM imidazole twice sequentially and centrifugation at 380*g*, 4 °C for 5 min after each wash to collect the supernatant. All fractions were checked by SDS–PAGE and those containing XT1 were pooled, concentrated to 600 µl by Vivaspin column (30-kDa molecular weight cutoff (MWCO); Cytiva) and buffer-exchanged with 50 mM Tris-HCl pH 7.5 with 150 mM NaCl and 20% (v/v) glycerol. Protein concentration was measured by NanoDrop One (Thermo Fisher Scientific) as 0.05–0.12 mg ml^−1^ (yield: 30–72 μg). Protein was aliquoted into 50 µl per aliquot, flash-frozen in liquid nitrogen and stored at −80 °C.

### Analyses of enzyme specificities of XT1 mutants to UDP-Xyl, UDP-Glc and UDP-6-Azido-Glc

A bikunin-derived peptide described previously^17^ was conjugated to 5-carboxyfluorescein (FAM) through an ɛ-aminohexoic acid (ɛ-AHx) linker resulting in 5FAM-ɛ-Ahx-GQEEEGSGGGQGG-CONH_2_ (5FAM-bik) as described previously^7^. Glycosylation assays with a total volume of 25 µl were carried out using 100 µM 5FAM-bik peptide, 0.2 µM purified XT1 (WT or mutants) and 200 µM UDP-Xyl, UDP-Glc or UDP-6-azido-6-deoxy-Glc (Biosynth) and incubated in reaction buffer (50 mM Tris-HCl pH 7.0 with 50 mM NaCl) at 37 °C overnight. The reaction mixtures were then boiled at 95 °C for 2 min to stop the glycosylation reaction and were briefly centrifuged. Of each reaction mixture, 5 µl was then run on an Acquity H-Class PLUS qDA ultra-HPLC (UPLC)–MS (Waters) equipped with an Acquity UPLC glycan BEH amide column (130 Å, 1.7 µm, 2.1 × 100 mm). Samples were run at flow rate of 0.35 ml min^−1^ using buffer A (10 mM ammonium formate at pH 4.5) and buffer B (10 mM ammonium formate in 90:10 acetonitrile and water). The percentage of turnover of 5FAM-bik peptide into the corresponding glycopeptides was calculated by integration of the HPLC ultraviolet trace (260 nm absorption) of the peptide and the glycopeptide and calculated as turnover percentage = peak area of glycopeptide/peak area of (5FAM-bik + glycopeptide).

### Enzyme kinetics of WT-XT1 and BH-XT1

Enzyme concentration titration was carried out to identify the concentration of WT-XT1 and BH-XT1 required for a turnover of maximum 10–20%. For purified XT1 preparations, a serial dilution from 500 nM to 3.9 nM was used in reaction buffer. Following this, 200 µM UDP-sugar and 100 µM synthetic fluorescent peptide 5FAM-bik were added with a total volume of 25 µl. The reaction mixtures were incubated at 37 °C for 1.5 h, stopped by incubation at 95 °C for 2 min and then briefly centrifuged. Aliquots of 5 µl of the reaction mixtures were checked by HPLC and the turnover rate was calculated to give 16 nM WT-XT1 for incubation with UDP-Xyl, 30 nM XT1-Leu526Gly (BH-XT1) for incubation with UDP-6AzGlc and 100 nM BH-XT1 for incubation with UDP-Xyl as suitable concentrations for Michaelis–Menten kinetics. A serial dilution of UDP-sugar from 400 to 3.125 µM was run in glycosylation reactions in 20 µl of reaction buffer with 100 µM synthetic fluorescent peptide 5FAM-bik and the optimal concentration of WT-XT1 or BH-XT1. The reaction mixtures were incubated at 37 °C for 1.5 h; reactions were stopped by incubation at 95 °C for 2 min and briefly centrifuged. Aliquots of 5 µl of the reaction mixtures were checked by HPLC; the turnover was calculated and transformed into rate with the formula 1,000 × ((peak area of glycopeptide/peak area of (5FAM-bik + glycopeptide)) × 100 µM)/5,400 s to give the rate *v* in nM s^−1^. The kinetics curve was then plotted with Prism 10 (GraphPad) and fitted with a Michaelis–Menten function to calculate *k*_cat_, *K*_M_ and *v*_max_.

### Peptide specificity analyses

BH-XT1 activity toward a collection of substrate peptides was assessed by using a UDP-Glo GT assay kit (Promega) following the manufacturer’s instructions. The collection of 240 peptides was described previously^17^ and their sequences are shown in the heat map in Fig. 2. In brief, 25-µl reactions contained 25 nM BH-XT1, 100 µM UDP-6AzGlc and 25 µM peptide in 50 mM Tris-HCl pH 7.0 with 50 mM NaCl in 96-well plates. Reactions were shaken at 350 rpm for 30 s on a thermomixer (Thermo Fisher Scientific) at room temperature and then incubated at room temperature for 1 h followed by adding 25 µl of freshly prepared UDP-Glo reagent for detection. A series of diluted UDP standards from 0 to 25 µM were included in each set of experiments to plot the standard curve and blank UDP-6AzGlc was run as background subtraction. Luminescence was read using a plate-reader (Tecan) with luminescence readout at 1,000 ns of integration. Luminescence values were background-substracted using the UDP-6AzGlc blank, normalized to the average luminescence of WT bikunin peptide run in parallel, and the resulting percentage turnover relative to WT is presented as a heat map. The Pearson correlation coefficients and *P* values were calculated using the pearsonr function in the scipy.stats Python library.

### In vitro glycosylation of membrane fractions

CHO cells with a CRISPR KO for either *Xylt1* or *Xylt2* or parental CHOZN GS^−/−^ (WT CHO cell with glutamine synthetase KO) were cultured in EX-CELL CD CHO fusion medium (Sigma-Aldrich) supplemented with 2% Glutamax (Thermo Fisher Scientific) as described previously^10^. Supernatants (15 ml) were collected when cells were at a density of 1 × 10^6^ cells per ml, concentrated with Amicon ultra centrifugal filters (30-kDa MWCO; Merck), aliquoted and stored at −80 °C. Cell pellets were treated with the subcellular fractionation kit for cultured cells (Thermo Fisher Scientific) following the manufacturer’s instructions. Membrane fraction protein concentrations were measured using a Pierce BCA protein assay (Thermo Fisher Scientific). Glycosylation reactions were run in 25 µl of 50 mM Tris-HCl pH 7.0 with 50 mM NaCl with 16 µg of membrane protein, 200 nM WT-XT1 or BH-XT1, 250 µM UDP-6AzGlc and 100, 200 or 300 µM UDP-Xyl. The reaction mixtures were incubated at 37 °C overnight, treated with 7.5 µl of a click reaction mastermix (final concentrations: 1,200 µM BTTAA, 600 µM CuSO_4_, 100 µM biotin-alkyne, 5 mM sodium ascorbate and 5 mM aminoguanidinium chloride), incubated at room temperature overnight and quenched by the addition of 3 µl of 50 mM EDTA. Reaction mixtures were then subjected to SDS–PAGE and western blot. The total protein and streptavidin signal was recorded as described above.

Competition glycosylation reactions were run as described above, except that membrane fractions were preincubated with WT-XT1 and UDP-Xyl before the addition of BH-XT1–UDP-6AzGlc in the indicated concentrations and incubating for another 16 h. Reactions were treated as described above.

### In vitro glycosylation of recombinant, GAG-free glypican 1

GAG-free glypican 1 was prepared as reported previously^7^. For each sample, the 25-µl reaction mixture contained 200 nM WT/BH-XT1, 250 µM UDP-Xyl and/or UDP-6AzGlc and 16 µM glypican 1 in 50 mM Tris-HCl pH 7.0 and 50 mM NaCl. Reactions contained WT-XT1 and UDP-Xyl (A and D), WT-XT1, UDP-Xyl and UDP-6Az-Glc (B) or BH-XT1, UDP-Xyl and UDP-6AzGlc (C). Reactions A–D were incubated at 37 °C overnight, after which 200 nM BH-XT1 and 250 µM UDP-6AzGlc were added to reaction D, which was then incubated for an additional 16 h. Following this, 7.5 µl of a click reaction mastermix was added to give a final concentration of 1,200 µM BTTAA (Jena Bioscience), 600 µM CuSO_4_ (Sigma-Aldrich), 100 µM biotin-alkyne (Biotium), 5 mM sodium ascorbate (Thermo Fisher Scientific) and 5 mM aminoguanidinium chloride (Cayman Chemical). Click reactions were carried out at room temperature overnight at 350 rpm and later quenched by the addition of 3 µl of 50 mM EDTA. Reaction mixtures were subjected to SDS–PAGE and western blot. Total protein content was measured on an Odyssey CLx (LI-COR Biosciences) using the Revert total protein kit (LI-COR Biosciences) and biotinylation was assessed with IRDye 800CW streptavidin (LI-COR Biosciences). FLAG-tagged decorin was visualized with a rabbit polyclonal antibody (Invitrogen, PA1-984b) and a 700CW anti-rabbit antibody (LI-COR Biosciences).

### In vitro glycosylation of recombinant, GAG-free decorin

GAG-free decorin in pgsA-745 CHO cells was prepared previously^7^. For each sample, a 25-µl reaction mixture contained 200 nM WT/BH-XT1, 250 µM UDP-Xyl and/or UDP-6AzGlc and 0.64 µM decorin in 50 mM Tris-HCl pH 7.0 and 50 mM NaCl. Reactions contained WT-XT1 and UDP-Xyl (A and D), WT-XT1, UDP-Xyl and UDP-6Az-Glc (B) or BH-XT1, UDP-Xyl and UDP-6AzGlc (C). Reactions A–D were incubated at 37 °C overnight, when 200 nM BH-XT1 and 250 µM UDP-6AzGlc were added to reaction D, which was then incubated for an additional 16 h. Following this, 7.5 µl of a click reaction mastermix was added to give a final concentration of 1,200 µM BTTAA, 600 µM CuSO_4_, 100 µM biotin-alkyne, 5 mM sodium ascorbate and 5 mM aminoguanidinium chloride. Click reactions were carried out at room temperature overnight at 350 rpm and later quenched by the addition of 3 µl of 50 mM EDTA. Reaction mixtures were subjected to SDS–PAGE and western blot. Total protein content was measured on an Odyssey CLx (LI-COR Biosciences) using the Revert total protein kit (LI-COR Biosciences) and biotinylation was assessed with IRDye 800CW Streptavidin (LI-COR Biosciences). FLAG-tagged decorin was visualized with rabbit polyclonal anti-FLAG and a 700CW anti-rabbit antibody (LI-COR Biosciences).

### Sample prep for MS analyses of glycosylated decorin

GAG-free decorin protein (10 µg) was incubated with 250 µM UDP-6AzGlc and 200 nM BH-XT1 in 50 mM Tris-HCl pH 7.0 with 50 mM NaCl in a 30-µl total reaction volume at 37 °C overnight. The glycosylation reaction was treated with 7.5 µl of a click reagent mastermix containing 1-(3-butyn-1-yl)-3-methylimidazolium tetrafluoroborate described before^45^ and termed ITag-alkyne (final concentrations: 1,200 µM BTTAA, 600 µM CuSO_4_, 100 µM Itag-alkyne, 5 mM sodium ascorbate and 5 mM aminoguanidinium chloride). The reaction was incubated by shaking at 350 rpm on a thermomixer at room temperature overnight. The clicked decorin sample was digested by trypsin in solution with S-trap (Protifi) following the manufacturer’s instructions.

### MS data acquisition for glycopeptide analysis

Samples were analyzed by online nanoflow LC–tandem MS using an Orbitrap Eclipse Tribrid MS instrument (Thermo Fisher Scientific) coupled to a Dionex UltiMate 3000 HPLC (Thermo Fisher Scientific). For each analysis, 14 µl was injected onto a trap column (Acclaim PepMap 100, 75 µm × 2 cm, NanoViper) with loading buffer (2% acetonitrile and 0.05% TFA) at 7 μl min^−1^ for 6 min (40 °C). Glycopeptides were then separated on an analytical column (PepMap RSLC C18, 75 µm × 50 cm, 2-µm particle size, 100-Å pore size, reversed-phase EASY) using a gradient of 2–40% solvent B (5% DMSO, 0.1% formic acid, 75% acetonitrile and 20% water) over 140 min at 275 nl min^−1^.

Full-scan MS1 spectra acquired in the Orbitrap were collected at a resolution of 120,000 at full width at half-maximum and a mass range from 300 to 1,500 *m*/*z*.

Dynamic exclusion was enabled with a repeat count of three, repeat duration of 10 s and exclusion duration of 10 s. Only charge states 2–6 were selected for fragmentation. MS2 scans were generated at top speed for 3 s. HCD was performed on all selected precursor masses with the following parameters: isolation window of 2 *m*/*z*, 28% normalized collision energy, orbitrap detection (resolution of 30,000), maximum inject time of 54 ms and a standard automatic gain control target. An additional ETD fragmentation of the same precursor was triggered if (1) the precursor mass was between 300 and 1,500 *m*/*z* and (2) the fingerprint ion generated by the specific tag (322.1507 for ITag-Alkyne) was present at ±0.1 *m*/z and greater than 10% relative intensity.

### MS data analysis of glycopeptides

Raw files were searched using Byonic (Protein Metrics, version 4.6.1). For glycopeptide analysis, search parameters included semispecific cleavage specificity at the C-terminal sites of Arg and Lys, with two missed cleavages allowed. Mass tolerance was set at 10 ppm for MS1s, 20 ppm for HCD MS2s and 0.2 Da for ETD MS2s. Carbamidomethyl cysteine was set as a fixed modification. Variable modifications included methionine oxidation and asparagine deamidation. *O*-glycan modification was set to *N*-acetyl-hexosamine with an additional 118.0643 *m*/*z* to account for the chemical modification. A maximum of two variable modifications were allowed per peptide. For each sample, variable modifications were searched against a focused FASTA file that exclusively contains protein sequences find in that sample. Identifications that contained chemically modified glycans on the peptide of interest were manually validated and localized using a combination of HCD and ETD information.

### Analysis of UDP-sugar biosynthesis

This protocol was adapted from previously published procedures^35,43,48^. K-562 cells (American Type Culture Collection, CCL-243) were cultured in RPMI (Thermo Fisher Scientific) with 10% (v/v) FBS, penicillin (100 U per ml) and streptomycin (100 µg ml^−1^). Approximately 5–10 million cells (comparable numbers between treatment conditions) were fed with DMSO or 250 µM caged sugar-1-phosphates **1** or **2**. After 16 h of incubation, K-562 cells were harvested by centrifugation (500*g*, 5 min, 4 °C). Cells were washed with ice-cold PBS twice and zirconia and silica beads (0.1 mm; Biospec) were added to packed cell pellets with a 1:1 ratio. Then, 1 ml of 1:1 acetonitrile and water was added to lyse the cells with a bead beater at 6 m s^−1^ for 30 s and the cell lysates were cooled at 4 °C for 10 min. They were then centrifuged at 14,000*g* for 10 min at 4 °C and the supernatant was transferred to a protein low-bind Eppendorf. Supernatants were dried down with a SpeedVac and the residue was dissolved in 0.3 ml of Milli-Q water. The supernatant was passed through a centrifuge filter (30 min, 14,000*g*) using an Amicon ultra centrifugal filter (3-kDa MWCO). The flowthrough was dried by SpeedVac and the residue resuspended in 50 µl of MQ water. High-performance ion-exchange chromatography was used to analyze lysates, using a Waters Arc Premier HPLC with photodiode array detector equipped with a Dionex CarboPac PA1 column (2 mm × 150 mm) and matching PA1 guard column at a flow rate of 0.25 ml min^−1^. The gradient with buffers A (1 M sodium acetate and 1 mM NaOH), B (1 mM NaOH) and C (1 M NaOH) was as follows: 0 min, 5% A, 95% B; 20 min 40% A, 60% B; 60 min, 40% A, 60% B; 63 min, 50% A, 50% B; 87 min, 80% A, 20% B; 95 min, 80% A, 20% B; 96 min, 5% A, 95% B; 101 min, 5% A, 95% B.

### Site-directed mutagenesis and cloning of full-length XT1

A full-length XT1 plasmid in pDonor221 vector was purchased from DNASU (HsCD00744626). Site-directed mutagenesis was performed using a Q5 HIFI mutagenesis kit following the manufacturer’s instructions using the two mutagenesis primers CTACACCCTCGGCCCCGCTGAGTC and GAGTAGAACTGTTTCATCTTG. Full-length WT/BH-XT1 DNA was then prepared using the two primers ATGGTGGCGGCGCCAT and CCGTAGCCGGCCATCAG. To add a VSV-G tag, a third PCR was performed using the primers ACCCCAAGCTGGCCTCTGAGGCCATGGTGGCGGCGCCATGCGCCCG and CCCCAAGCTTGGCCTGACAGGCCCTACTTACCCAGGCGGTTCATTTCGATATCAGTGTACCGTAGCCGGCCATCAG. pSBbi-GH was a gift from E. Kowarz (Addgene, plasmid 60514; RRID:Addgene_60514)^58^. The plasmid was linearized using restriction enzyme SfiI (New England Biolabs) following the manufacturer’s instructions and purified from agarose gel. Full-length WT-XT1 and BH-XT1 were then inserted into pSBbi-GH using the infusion cloning kit following the manufacturer’s instructions. Genes of interest were sequenced by Sanger sequencing and the full plasmids were sequenced by nanopore sequencing before use.

### Generation of K-562 XT-KO cells

RNA-guided DNA endonuclease was performed to edit genes through coexpression of the Cas9 protein and guide RNAs (gRNAs). The targeting sequences for XT1 and XT2 were at exon 3 of XYLT1 (5′-ACAACAGCAACTTCGCACCC-3′) and exon2 of XT2 (5′-GACAGTTCAGCAGGGCGACG-3′), respectively. The target sequences were cloned into the gRNA cloning vector using the restriction enzyme BsmBI (New England Biolabs, R3539). KO cells were all obtained through clonal propagation from a single cell. For genotyping, the following PCR primers were used: 5′-CGGGACGCTGGAACAAAATG-3′ and 5′-GGGGTTGGAACTTACCCTCG-3′ for XT1 alleles; 5′-GGTGGTACTGATTGTGCGGA-3′ and 5′-CAGGGAGGTAGGATCCCCTT-3′ for XT2 alleles. PCR products were sequenced.

### Stable transfection of pgs745 cells with full-length WT-XT1 and BH-XT1

The plasmid pCMV(CAT)T7-SB100 was a gift from Z. Izsvak (Addgene, plasmid 34879; RRID:Addgene_34879)^70^. PgsA-745 CHO cells were cultured in growth medium to 0.5 × 10^6^ cells per ml for 24–48 h before transfection. Next, 2.5 µg of pSBbi plasmid containing full-length WT-XT1 or BH-XT1 was transfected together with 125 ng of pCMV(CAT)T7-SB100 plasmid per well of a six-well plate using Lipofectamine LTX (Thermo Fisher Scientific) according to the manufacturer’s instructions. After 24 h, cell culture medium was aspirated and cells were treated with fresh growth medium containing 200 µg ml^−1^ hygromycin B. Cells were cultured under these conditions for 2 weeks to obtain stable cells. Following selection, cells were propagated with 150 µg ml^−1^ hygromycin B in growth medium.

### Metabolic cell-surface labeling and in-gel fluorescence

Stably transfected pgsA-745 CHO cells with full-length WT-XT1 or BH-XT1 were plated in six-well plates at a density of 0.4 × 10^6^ cells per ml in growth medium without hygromycin B and then treated with the indicated concentration of caged sugar-1-phosphates **1** or **2**, Ac_4_ManNAz (Jena Bioscience) or DMSO. Cells were grown for 16 h. The cell culture medium was aspirated and cells were washed with cold PBS without Ca^2+^ or Mg^2+^. The cells were then detached with 1 ml of ice-cold 8 mM EDTA for 20 min at 4 °C. Cells were transferred to a 1.5-ml cup and harvested by centrifugation at 500*g* for 5 min at 4 °C.

Cells were then resuspended in 200 µl of cell buffer (2% FBS in PBS), transferred to a V-shaped 96-well plate (Thermo Fisher) and harvested by centrifugation at 500*g* for 5 min at 4 °C. The cells were resuspended in 35 µl of cell buffer and treated with 35 µl of click solution mastermix (200 µM CuSO_4_, 1200 µM BTTAA, 10 mM sodium ascorbate, 10 mM aminoguanidinium chloride and 50 mM CF680-alkyne in cell buffer). Cells were briefly mixed and incubated for 7 min at room temperature on an orbital shaker. The reaction was quenched by the addition of 35 µl of 3 mM bathocuproinedisulfonic acid in PBS. Cells were then harvested, washed twice with 200 µl of cell buffer and once with PBS and then resuspended in 100 µl of ice-cold lysis buffer (50 mM Tris-HCl pH 8 with 150 mM NaCl, 1 mM MgCl_2_, 0.5% (w/v) sodium deoxycholate, 0.1% (w/v) SDS, 1% (w/v) Triton X-100, 1× Halt protease inhibitor and 100 mU per µl of benzonase (Merck)). Cells were lysed for 20 min at 4 °C on an orbital shaker and centrifuged at 1,500*g* for 20 min at 4 °C. The supernatants were then transferred to 1.5-ml protein LoBind tubes (Eppendorf). The protein concentration was measured using Pierce BCA protein assay kits before in-gel fluorescence. Samples were then analyzed after SDS–PAGE by in-gel fluorescence on an Odyssey CLx (LI-COR Bosciences). Total protein content was then assessed by Coomassie staining on the same gel using SafeBLUE protein stain (NBS Biologicals). Another gel was prepared and transferred to a nitrocellulose membrane for western blot, using antibodies to rabbit VSV-G tag (Abcam, ab50549) and GAPDH (Abcam, ab181602) and secondary antibodies IRDye 800CW donkey anti-mouse (LI-COR) and IRDye 680RD donkey anti-rabbit IgG (LI-COR). The image background was adjusted by LI-COR software.

### Chemoenzymatic GAG linker synthesis on 5FAM-bik peptide

Xyl-containing or 6AzGlc-containing glycopeptides were generated enzymatically in a 100-µl reaction with 100 µM 5FAM-bik peptide, 200 nM WT-XT1 and 200 µM UDP-Xyl or 200 nM BH-XT1 and 200 µM UDP-6AzGlc, respectively. When full conversion was reached, glycopeptides were desalted with a Strata-X (60 mg ml^−1^) Phenomenex solid-phase extraction column following the manufacturer’s instructions and dried with a Genevac miVac centrifugal concentrator (Fisher Scientific) before downstream one-pot enzyme reactions. These were carried out in 50 mM Na-HEPES pH 7.5, 25 mM MnCl_2_ and 50 mM NaCl in a total reaction volume of 20–30 μl as reported previously^7^. Soluble enzymes for extension (B4GALT7, MBP–B3GALT6 and B3GAT3) were prepared previously^7^. These enzymes were added at 0.025 μg ml^−1^ in different combinations to glycopeptides at 500 μM and the UDP-sugars UDP-Gal and UDP-GlcA were added as needed at a twofold molar excess to the acceptor. The reaction mixture was left at 30 °C overnight and the reaction progress was monitored by HPLC as reported previously^7^.

### Expression and cellular glycosylation by BH-XT1 of decorin in pgsA-745 cells

An expression construct of human decorin containing a C-terminal FLAG tag was prepared previously^7^. Cultured pgsA-745 cells stably transfected with full-length BH-XT1 were plated at 1 × 10^6^ cells per ml in 5 ml of growth medium in a T25 flask and grown to 100% confluency. The cells were then detached using 0.05% (v/v) Trypsin-EDTA (Thermo Fisher Scientific) and resuspended in 5 ml of fresh medium. While the cells were still in suspension, 5 µg of plasmid DNA and 15 µg of polyethylenimine MAX (40 kDa; Polysciences) were diluted separately in 250 µl of OptiMEM (Thermo Fisher Scientific) and incubated at room temperature for 5 min. Both solutions were mixed and incubated at room temperature for another 20 min before added to the cell culture dropwise. The cells were incubated overnight to attach and 50 µM caged sugar-1-phosphate **1** was added. On the fourth day after transfection, the culture supernatant was collected. A 100-µl slurry of Pierce anti-DYKDDDDK affinity resin (Thermo Fisher Scientific) was washed with 30 CVs of FLAG buffer (25 mM HEPES with 150 mM NaCl) and added to the cell culture medium. The suspension was incubated at 4 °C overnight and then centrifuged at 4 °C at 1,000*g* for 5 min. The supernatant was removed and the beads were incubated with FLAG buffer containing 100 µg ml^−1^ FLAG peptide (Sigma-Aldrich) for 1 h at room temperature. An Amicon ultra centrifugal filter (3-kDa MWCO) was used to concentrate the protein to 300 µl and remove the FLAG peptide. The protein concentration was measured by BCA and the final yield of the protein estimated as 120 µg.

An aliquot of 20 µg of purified decorin in 60 µl of FLAG buffer was treated with 30 µl of CuAAC mastermix (final concentrations: 1,200 µM BTTAA, 600 µM CuSO_4_, 100 µM Itag-alkyne, 5 mM sodium ascorbate and 5 mM aminoguanidinium chloride) overnight at room temperature.

The clicked decorin sample was digested by trypsin in solution with S-trap (Protifi) following the manufacturer’s instructions. MS data acquisition and analysis were performed as described above.

### Site-directed mutagenesis of full-length XT2 and cloning into pSBbi plasmids

A full-length XT2-pJF7_nHalo vector was purchased from DNASU (accession number HsCD00866744). Site-directed mutagenesis was performed by overlap extension as described above using the mutagenesis primers AGAAGGACTCGGCTGGGCCCAGTGTGTATGTGTAG and CTACACATACACACTGGGCCCAGCCGAGTCCTTCT, as well as ATGGTGGCGAGCGCGCGAG and CAACCTGAGTCGCCCGTCTG. PCR reaction conditions were as stated above. Following assembly of the full-length XT2 gene, another PCR was performed using the primers AACTACCCCAAGCTGGCCTCTGAGGCCATGGTGGCGAGCGCGCGAG and CCCCAAGCTTGGCCTGACAGGCCCTACTTACCCAGGCGGTTCATTTCGATATCAGTGTACAACCTGAGTCGCCCGTC. The PCR product was inserted into pSBi-GH by infusion cloning. Genes of interest were sequenced by Sanger sequencing and the full plasmids were sequenced by nanopore sequencing before use. Cell-surface labeling and in-gel fluorescence were performed as described above.

### Sample prep for proteomics analysis

Stably transfected pgsA-745 CHO cells with full-length WT-XT1 or BH-XT1 were seeded in T-75 flasks at a density of 1 × 10^6^ cells per ml in growth medium. After 6 h of incubation, cells were fed with either 50 µM or 250 µM caged sugar-1-phosphate **1** or DMSO. The cell culture medium used for feeding was BalanCD CHO growth A medium (Fujifilm Irvine Scientific). Cells were grown for 16 h. The cell culture medium (secretome) was harvested and centrifuged (350*g*, 5 min) to pellet debris. The secretome samples were concentrated using Amicon ultra centrifugal filters (3-kDa MWCO). The buffer was exchanged with PBS twice. The Pierce BCA protein assay kit was used to measure the protein concentration of secretome samples.

Secretome samples (0.50 mg each) were normalized up to 250 µl with PBS and incubated for 1 h at room temperature with 300 µl of Neutravidin bead slurry (Sera-Mag SpeedBeads neutravidin-coated magnetic beads, Cytiva), previously washed twice with PBS (200 µl each), to remove endogenous biotinylated proteins. The supernatant was collected and then incubated with PNGase F overnight at 37 °C to remove *N*-glycans. The reaction was then quenched by heating to 95 °C for 10 s with subsequent cooling at 4 °C. The samples were then treated with a 10× click solution mastermix (6 mM CuSO_4_, 12 mM BTTAA, 1 mM biotin-DADPS-alkyne (Vector Laboratories), 50 mM sodium ascorbate and 50 mM aminoguanidinium chloride) to a 1× final concentration. The click reaction was incubated overnight at room temperature under shaking (400 rpm). The reaction was passed through Amicon ultra centrifugal filters (3-kDa MWCO) to exchange the buffer with PBS.

The samples were then incubated with 350 µl of dimethylated Neutravidin bead slurry (previously washed twice with 200 µl of PBS) for 1 h at room temperature^41^. Supernatant was discarded and beads were washed with 1% (w/v) SDS (three times, 350 µl each), 6 M urea in PBS (three times, 350 µl each), 50 mM ammonium bicarbonate (AmBic; three times, 350 µl each) and 40% (v/v) LC–MS-grade acetonitrile (four times, 100 µl each). Beads were resuspended in 100 µl of AmBic containing 10 mM DTT and then incubated at 50 °C for 15 min. Beads were washed with AmBic (two times, 350 µl each) and 100 µl of 20 mM iodoacetamide in AmBic was then added. Samples were kept for 30 min in the dark. Iodoacetamide was then quenched by adding DTT 10 mM (final concentration). The beads were washed with AmBic (three times, 350 µl each) and then resuspended in 100 µl of AmBic. Next, 300 ng of Lys-C (MS grade; Promega) was added to beads, followed by overnight incubation at 37 °C. The supernatant was transferred to a new tube and 200 ng of trypsin gold (MS grade; Promega) were added. The digestion was left for 8 h at 37 °C. Peptides were desalted by UltraMicroSpin (The Nest group) according to the manufacturer’s protocol and vacuum-dried by SpeedVac.

Dried peptides were resuspended in 16 µl of 0.1% (v/v) formic acid in LC–MS-grade water, sonicated for 15 min in a water bath, vortexed briefly and harvested for 5 min at 18,000*g*. The peptides were then loaded on Evotips (Evosep) according to the manufacturer’s protocol. The data were acquired on TIMS TOF Pro2 (Bruker) coupled to an Evosep One LC system. For the LC separation, a standard 60SPD 2.3 method was used, separation was performed using an EV-1109 column and the column was heated to 40 °C during analyses. TIMS TOF Pro2 was operated in data-independent acquisition parallel accumulation–serial fragmentation mode, scan width was set to 100–1,700 *m*/*z* with ion mobility (1/*K*_0_) of 0.6–1.6 and ramp and accumulation time were locked at 100 ms.

Raw MS files were loaded into DIA-NN 1.8.1 for quantification and identification by using the *C*. *griseus* FASTA protein sequences database from UniProt for database search. Among the DIA-NN output files, both protein groups and peptide groups were uploaded into Perseus (version 2.0.11)^62^ to allow for data transformation and visualization and then into GraphPad for statistical analysis. Briefly, to visualize the results on Perseus, the proteingrougs.txt file or peptidegroups.txt file was uploaded, followed by transformation of all the values to log_2_(*x*). Then, the data were imputed to replace missing values from normal distribution. Data from three independent replicate experiments of each sample in a row were categorically annotated with the same name. Once annotated, a two-sample Welsh’s *t*-test was performed to statistically analyze the data. Welch’s *t*-test was performed between samples from BH-XT1-expressing versus WT-XT1-expressing cells and BH-XT2-expressing versus WT-XT2-expressing cells to generate *P* values. Multiple-hypothesis testing was corrected using the Benjamini–Hochberg procedure to calculate false discovery rates (FDRs). Protein hits were filtered using an FDR value ≤ 0.05. The scatter plot function was used to visualize the volcano plots.

### Preparation of alkyne-heparin

A previously published procedure was modified to produce alkyne-modified heparin^16,71^. Briefly, 20 mg of heparin (Iduron, HEP001) was dissolved in 94 µl of 100 mM sodium acetate and 100 mM aniline buffer (pH 5.5) and prewarmed to 55 °C. Warmed heparin was mixed with 6 µl of alkyne hydrazide (BroadPharm BP-28990, 1.68 mg in DMSO, 80 equivalents), and DMSO was added to bring the ratio of aqueous buffer to DMSO to 1:1. The mixture was protected from light, incubated at 55 °C for 72 h before dilution into 10 ml of PBS, filtered at 0.45 µm and dialyzed into Milli-Q H_2_O (48 h, buffer changed three times). The sample was then lyophilized. Proton nuclear magnetic resonance verified the conjugation of alkyne hydrazide to heparin.

### Production of SDC1 and SDC1_37_

The human SDC1 ectodomains were cloned into pET28a expression vectors for production in BL21 (DE3) *Escherichia* *coli*. SDC1_37_ refers to a variant ectodomain wherein the canonical GAG attachment site S37 is replaced by the unnatural amino acid *p*-propargyltyrosine (pPY) with an alkyne handle for click chemistry. This variant was produced as previously described^16^, wherein the expression plasmid was cotransformed into BL21 with pULTRA-CNF, which permits the incorporation of unnatural amino acids. pPY (400 mg L^−1^) was added to the bacterial culture during induction. Protein was purified using HisPur cobalt resin (Thermo Fisher). SDC1_37_ was treated with azide-containing heparin as previously described^16^.

### In vitro glycosylation of SDC1 with BH-XT1–UDP-6AzGlc

Recombinant SDC1 and SDC1_37_ were in vitro glycosylated with 6AzGlc using the procedure established for decorin described above. In brief, two 78.5-µl reaction mixtures containing 15 µM SDC1 or SDC1_37_, 200 nM BH-XT1, 250 µM UDP-Xyl and UDP-6AzGlc in 50 mM Tris-HCl pH 7.0 with 50 mM NaCl were incubated at 37 °C overnight. The enzymes were then deactivated by incubation of the reaction mixtures at 95 °C for 2 min. The glycosylation reaction mixtures were then buffer-exchanged with the reaction buffer using Amicon ultra centrifugal filters (3-kDa MWCO) to remove excess UDP-sugars. The supernatants containing glycosylated SDC1 and SDC1_37_ were then collected and dried under SpeedVac before proceeding to the next step to be clicked with the alkyne-heparin.

### SDC1 click reactions

6AzGlc-modified hSDC1 (100 µM) and alkyne-heparin (20 molar equivalents) were dissolved in PBS with aminoguanidinium chloride (5 mM) in protein LoBind Eppendorf tubes. Click reagents (320 µM CuSO_4_, 1,600 µM Tris(3-hydroxypropyltriazolylmethyl)amine and 21 mM sodium ascorbate) were added and the reactions were incubated at 37 °C. Reactions were monitored using an Ultimate 3000 ultra-HPLC system and WAX-10 (4 × 250 mm) column at 1.0 ml min^−1^ in 20 mM Tris (pH 7.5) buffer. After reaction completion (~16 h), 5 µl of HisPur cobalt resin beads (Thermo Fisher, 89966) and an equal volume of wash buffer (10 mM imidazole and PBS, 40 µL) were added to each reaction. This mixture was incubated on a VortexGenie with the multiple-sample attachment for 1 h at room temperature. The supernatant was removed and beads were washed twice with wash buffer (5 min, orbital shaker), before incubation with elution buffer (150 mM imidazole and PBS, 5 min, orbital shaker). Eluate was concentrated using an Amicon ultra centrifugal filter (3-kDa MWCO) and buffer-exchanged into PBS. The concentration of glycoconjugate products was measured on a NanoDrop One using absorbance at 205 nm.

### Cell spreading assay

MDA-MB-231 cells were treated with 200 nM pooled SDC1 TriFECTA Dicer substrate RNAs (hs.Ri.SDC1.13) using Lipofectamine RNAiMAX. Then, 24 h after transfection, 24-well plates were coated with 1× poly(D-lysine) (15 min, room temperature) before incubation with 10 µg ml^−1^ vitronectin (4 °C, overnight, rocking). The following day, the plate was washed twice with PBS and blocked in 2% BSA in DMEM (1 h, 37 °C). Cells were harvested with nonenzymatic dissociation buffer and remodeled in 96-well round-bottom plates with 10 µM cholPEGNTA (1 h, 37 °C) followed by SDC constructs (2 µM, 1 h, 37 °C). Cells were resuspended in DMEM + 10% FBS and allowed to adhere to vitronectin-coated plates (overnight, 37 °C). Cells were then fixed with 4% PFA in PBS and stained with rhodamine phalloidin (2 U per ml, 1 h, room temperature, rocking), followed by Hoechst staining. Cells were imaged on an EVOS M500 fluorescence microscope. The extent of cell spreading and cell number were counted by ImageJ macros using previously published methods^16^.

### Quantification of cell spreading

Microscopy images were analyzed using ImageJ software. Individual channel microscopy images were converted to 8-bit grayscale and segmented with the threshold function, with holes filled. The ‘analyze particles’ function was then used (size: 150–∞) to quantify cell spreading. This operation was performed on both Hoechst and rhodamine-conjugated phalloidin images to quantify the number of cells and extent of spreading, respectively. The extent of spreading was quantified by dividing the total rhodamine-positive area by the number of Hoechst-stained nuclei. Normalization was performed relative to the least spread cells and WT cells. Negative values were treated as 0. Measurements were taken from >6 images of selected areas of cells, which were chosen at random across three biological replicates. Image fields that showed significant autofluorescence from the plate perimeter were not used. Statistical analyses and graphs were generated using GraphPad Prism 10. Normalization was conducted as follows:

$$\mathrm{Percent}\,\mathrm{cell}\,\mathrm{spreading}=(\mathrm{value}-\min )/(\max -\min )\times 100$$

### Reporting summary

Further information on research design is available in the Nature Portfolio Reporting Summary linked to this article.

## Online content

Any methods, additional references, Nature Portfolio reporting summaries, source data, extended data, supplementary information, acknowledgements, peer review information; details of author contributions and competing interests; and statements of data and code availability are available at 10.1038/s41589-025-02113-w.

## Supplementary information

Supplementary Information Supplementary Figs. 1–13, Tables 1–3, Note, References, spectra and chromatograms (Supplementary Figs. 14–31) and unprocessed blots for supplementary figures.

Reporting Summary

Supplementary Data 2 Substrate dependance of recombinant WT and BH-XT1 with UDP-sugars.

Supplementary Data 3 Michaelis–Menten kinetics of WT and BH-XT1 with UDP-Xyl and UDP-Glc.

Supplementary Data 4 BH-XT1 peptide substrate preference.

Supplementary Data 5 Pearson correlation of BH-XT1 with WT-XT1 across three independent datasets.

Supplementary Data 9 Proteomics of pgsA-745 secretome expressing BH-XT1 versus WT-XT1 (250 μM compound 1).

Supplementary Data 10 Proteomics of pgsA-745 secretome expressing BH-XT1 versus WT-XT1 (50 μM compound 1).

Supplementary Data 11 Proteomics of pgsA-745 secretome expressing BH-XT1 versus WT-XT1 (50 μM compound 1, individual peptides).

Supplementary Data 12 Proteomics of pgsA-745 secretome expressing BH-XT1 or BH-XT2 (50 μM compound 1 or DMSO).

## Source data

Source Data Fig. 1 In vitro enzymatic conversion data for Fig. 1c.

Source Data Fig. 1 In vitro enzymatic kinetics data for Fig. 1d.

Source Data Fig. 2 Peptide substrate preference data for Fig. 2a.

Source Data Fig. 2 Glycoproteomics evaluation data for Fig. 2e.

Source Data Fig. 2 Unprocessed blots for Fig. 2b–d.

Source Data Fig. 3 HPLC traces for Fig. 3c.

Source Data Fig. 4 Unprocessed blots for Fig. 4b,d.

Source Data Fig. 5 HPLC traces for Fig. 5a.

Source Data Fig. 5 Glycoproteomics evaluation data for Fig. 5c.

Source Data Fig. 5 Proteomics data for Fig. 5d,e.

Source Data Fig. 5 Cell spreading analysis data for Fig. 5f.

## Data Availability

Proteomics and glycoproteomics data were uploaded to ProteomeXchange through the MassIVE server under accession numbers MSV000098977, MSV000098983, MSV000098981 and MSV000098982. The data supporting the findings of this study are available within the paper and its Supplementary Information. Should any raw data files be needed in another format, they are available from the corresponding author upon reasonable request. Source data are provided with this paper.
