# Supplementary material for: Xylosyltransferase engineering to manipulate proteoglycans in mammalian cells
Source: Nat Chem Biol. 2026 Jan 20;22(4):612–21. doi: 10.1038/s41589-025-02113-w (PMC13038410; doi:10.1038/s41589-025-02113-w)

nature chemical biology

Supplementary information https://doi.org/10.1038/s41589-025-02113-w

Xylosyltransferase engineering to manipulate proteoglycans in mammalian cells

In the format provided by the authors and unedited

Supplementary Information

Xylosyltransferase Engineering to Manipulate Proteoglycans in Mammalian Cells

Table of Contents

Item	Page number
Supplementary Figures 1-11	**2-12**
Supplementary Table 1	**13**
Supplementary Figures 12-13	**14-15**
Supplementary Tables 2-3	**16-17**
Supplementary Note	**17**
Supplementary Reference	**18**
Supplementary Figures for Spectra and Chromatograms of Compounds (Contents)	**19**
Supplementary Figures for Spectra and Chromatograms of Compounds (Spectra and Chromatograms)	**20-41**
Additional Supplementary Figures	**42-43**

Supplementary Figures

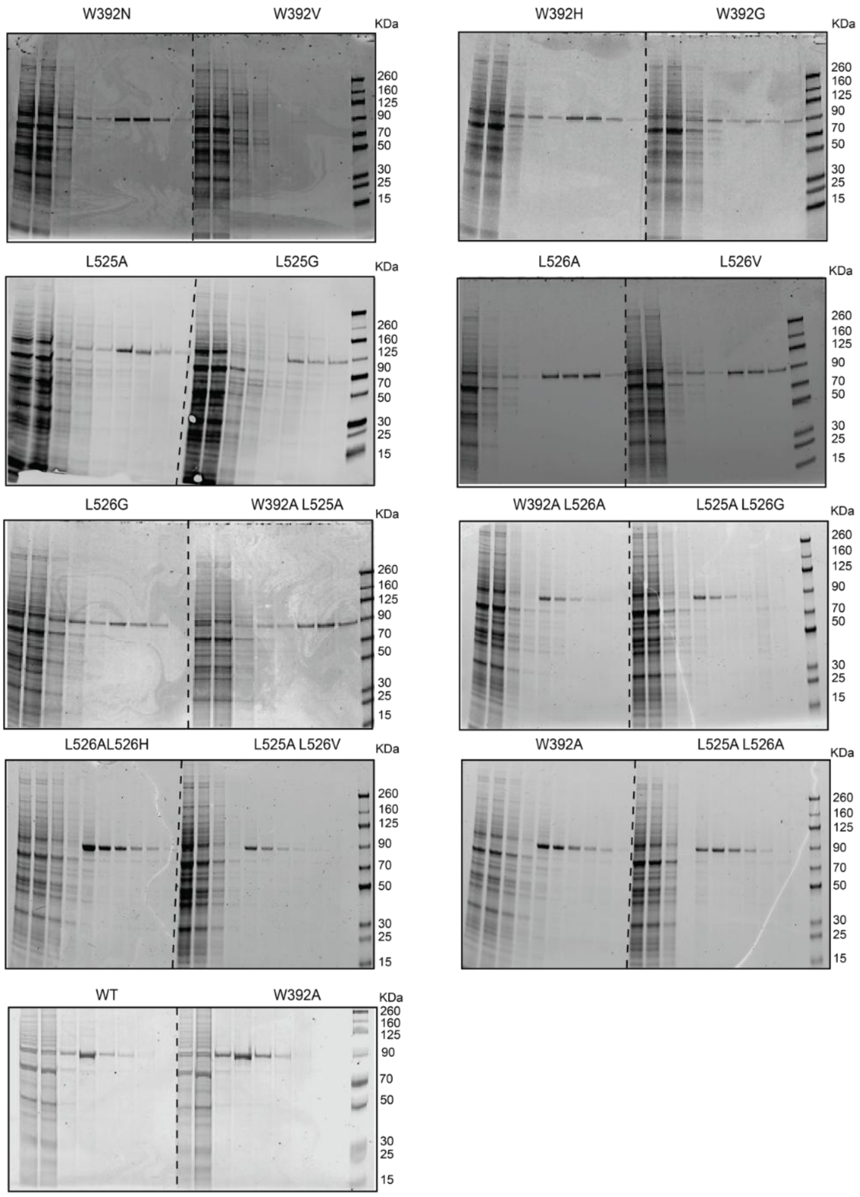

Supplementary Fig. 1: Expression of XT1 constructs in Expi293F cells on 10 mL scale purified by Ni-NTA affinity chromatography and visualised by SDS-PAGE and Coomassie staining. Fractions are from left to right: Lane 1 Input, 2 flow-through, 3-5 20 mM imidazole, 6-7 50 mM imidazolde, 8-9 200 mM imidazole. Note that the final fraction was omitted for some constructs. Gels are uncropped.

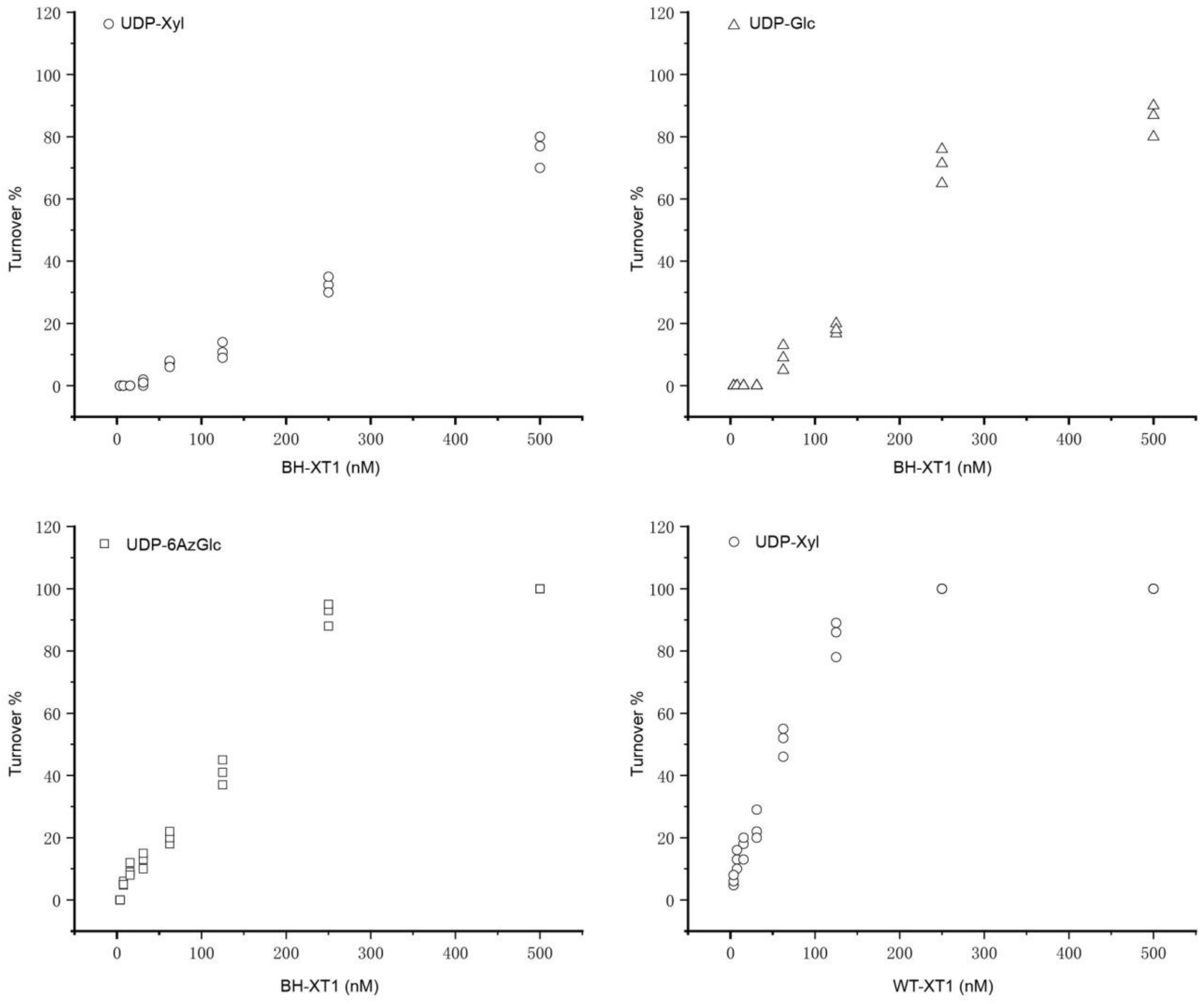

Supplementary Fig. 2: Enzyme dependence of XT1 *in vitro* glycosylation reactions. Turnover of fluorescent bikunin substrate peptide was assessed in reactions containing constant concentrations of peptide and UDP-sugar. Enzyme concentration was varied. Analysis was performed by HPLC and plots were used to determine the enzyme concentration required to achieve 10-20% conversion. Data are individual data points from n = 3 technical replicates.

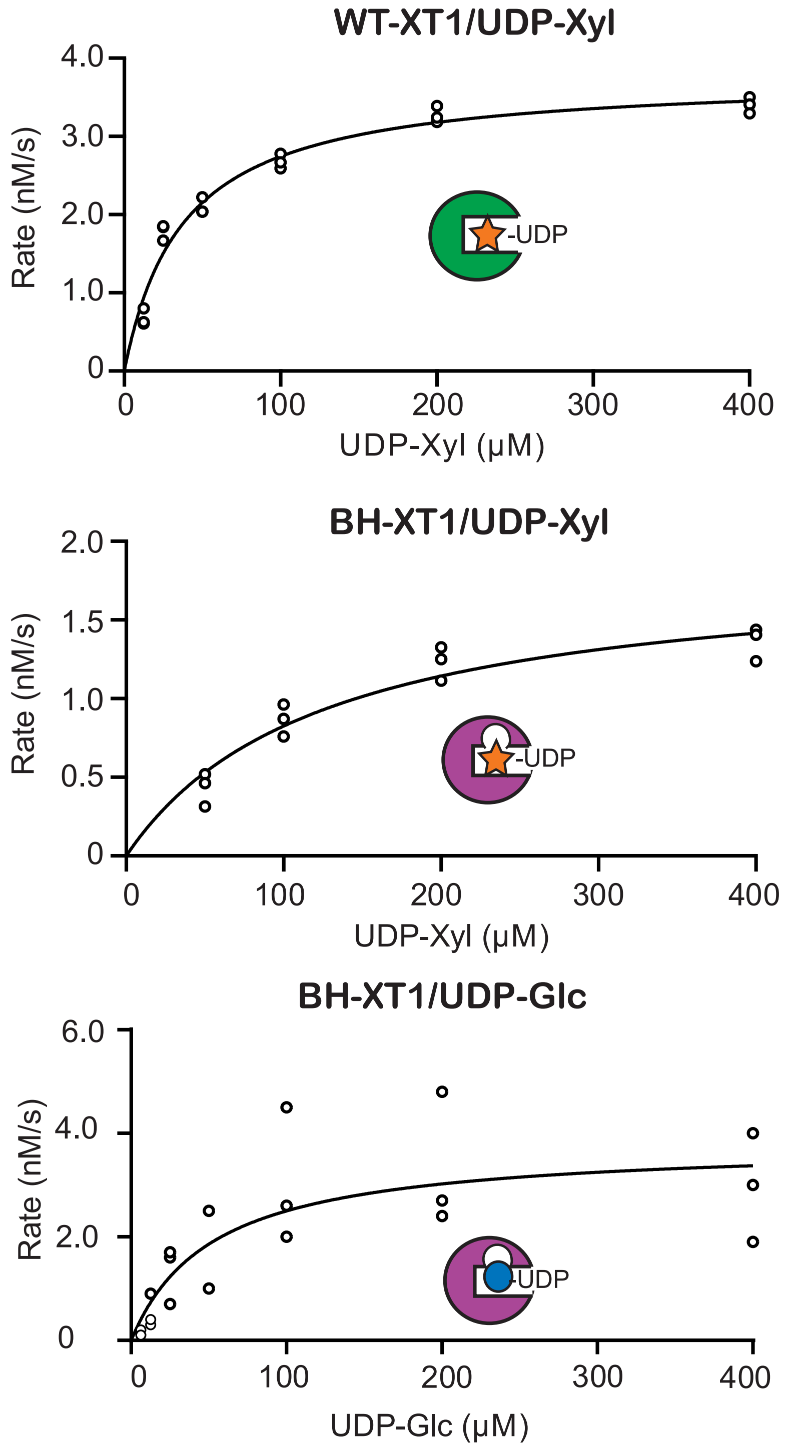

Supplementary Fig. 3: Michaelis-Menten kinetics of the indicated enzyme-substrate pairs. Data are individual data points of n = 3 technical replicates (WT-XT1/UDP-Xyl, BH-XT1/UDP-Xyl) or of n = 3 independent replicates (BH-XT1/UDP-Glc).

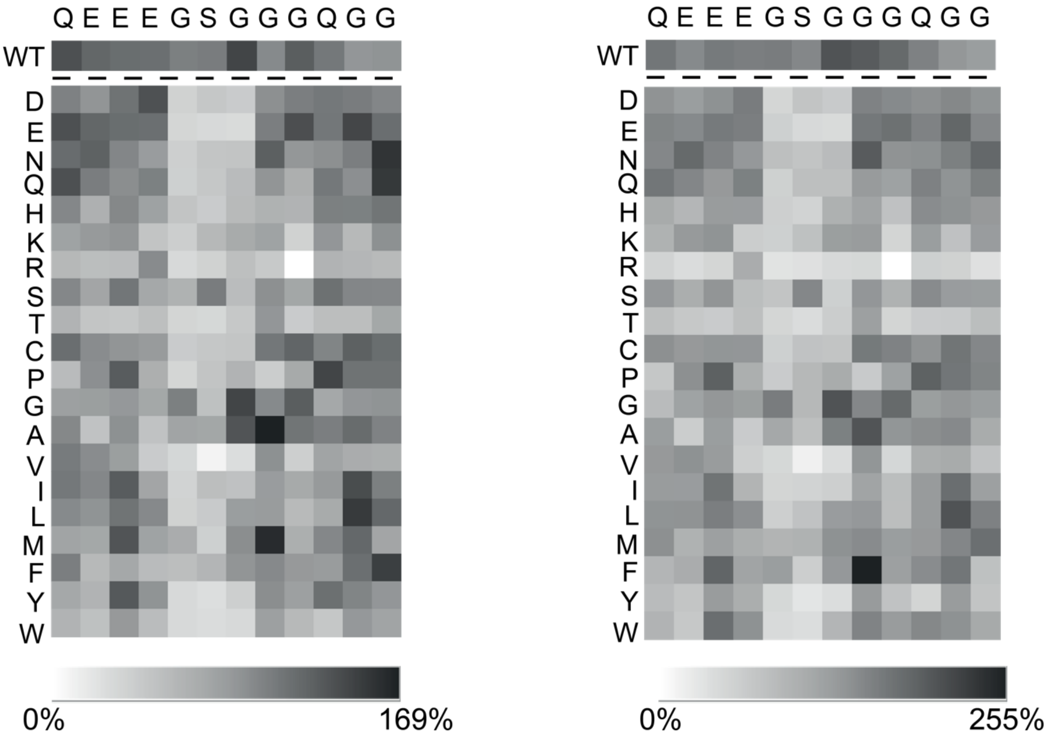

Supplementary Fig. 4: Independent replicates of *in vitro* glycosylation with the bikunin peptide substrate panel. Data were generated as in Fig. 2a.

Dataset	Pearson correlation	P-value
Set1 VS. WT	0.402	<0.001
Set2 VS. WT	0.456	<0.001
Set3 VS. WT	0.416	<0.001

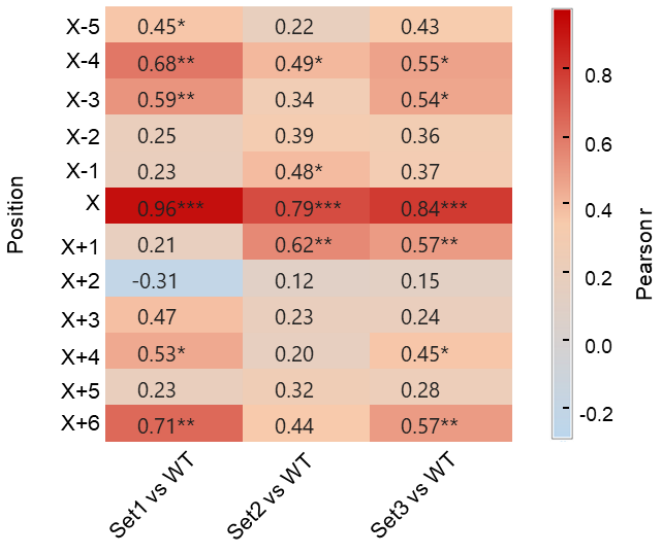

Pearson Correlation (per position) vs WT
(*p<0.05, **p<0.01,*p<0.001)**

Supplementary Fig. 5. Pearson correlation analysis was performed on the 11 amino acid positions of BH-XT1 relative to WT-XT1 activities across three independently generated datasets (Set1, Set2, and Set3) measured using a 240-peptide array. Left panel: Pearson correlation coefficients (*r*) and corresponding *P* values summarizing the correlation between each BH-XT1 dataset (Set1–Set3) and the corresponding WT-XT1 activity data. Right panel: Heat map showing pairwise Pearson correlation coefficients among different amino acids. The colour scale represents the degree of correlation (red, positive; blue, negative). Asterisks indicate levels of statistical significance (*P < 0.05, **P < 0.01, ***P < 0.001). Statistical analyses were performed using the pearsonr function in the *scipy.stats* library with python, which applies a two-tailed Student's *t*-test to determine significance. Exact *P* values are provided in the Source Data file for Supplementary Figure 5.

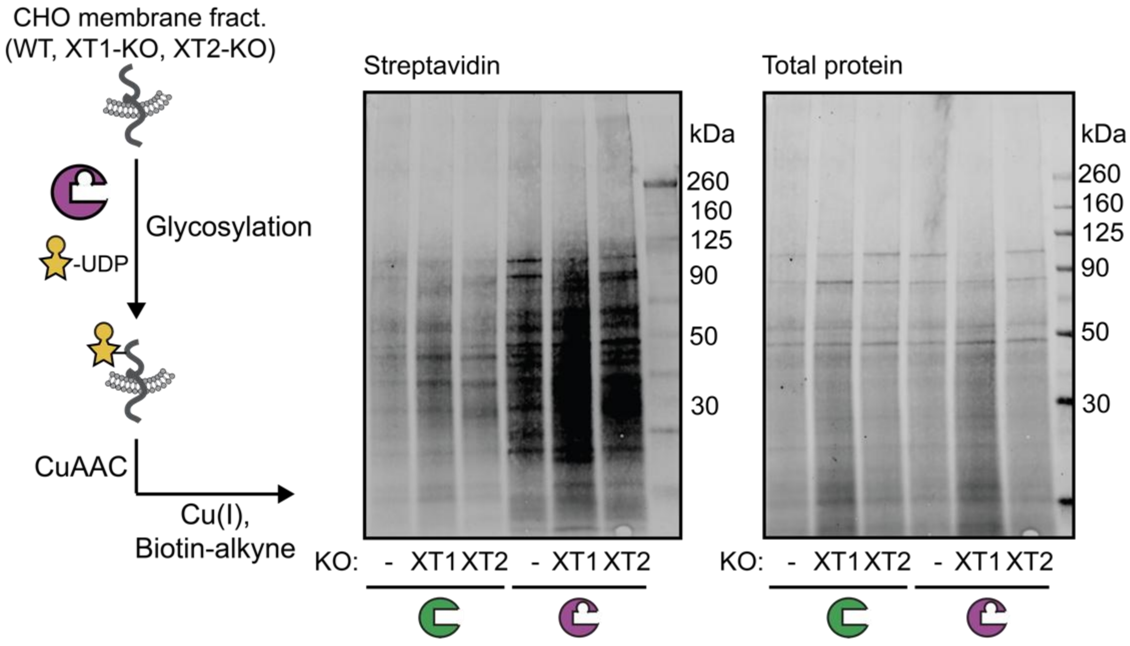

Supplementary Fig. 6: *in vitro* glycosylation of a membrane protein preparation of parental, *Xylt1*-KO or *Xylt2*-KO CHO cells as assessed by streptavidin blot. Reactions contained 250 µM UDP-6AzGlc and were reacted with biotin-alkyne before blotting as in Fig. 2b. Data are from one experiment.

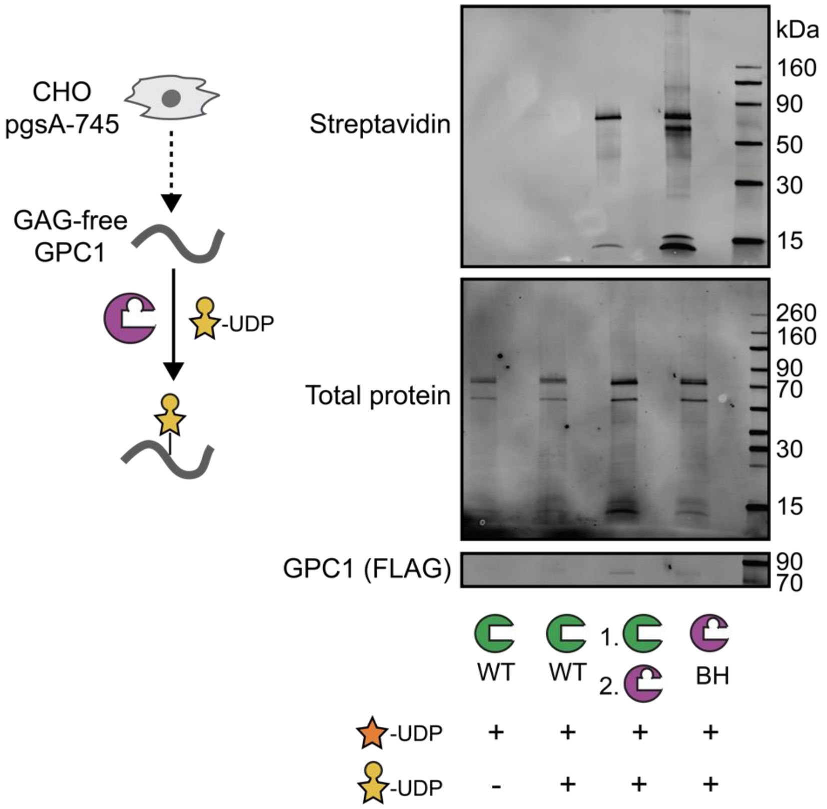

Supplementary Fig. 7: *in vitro* glycosylation of a GAG-free preparation of FLAG-tagged human glypican-1 from pgsA-745 CHO cells as assessed by streptavidin blot. Reactions contained 250 µM UDP-sugars and were processed with or without pre-incubation with WT-XT1/UDP-Xyl as indicated. Data are from one out of n = 2 independent replicates.

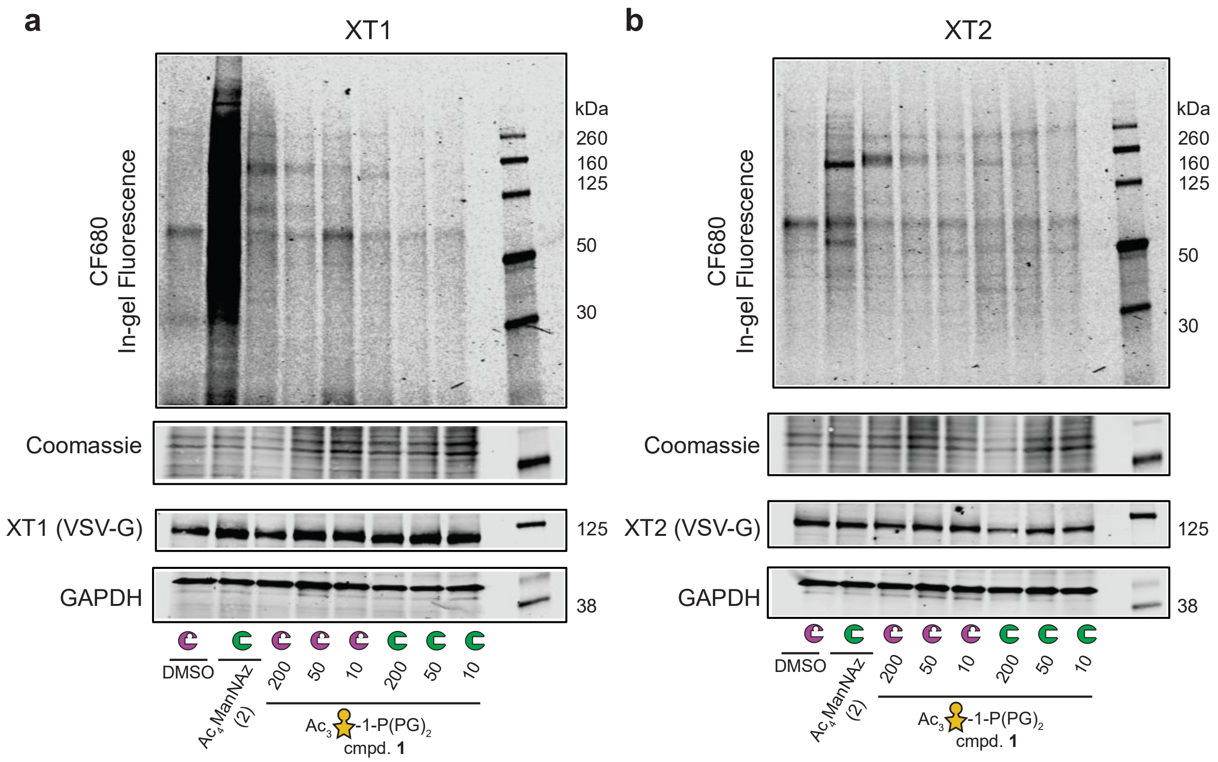

Supplementary Fig. 8: Chemical tagging of proteoglycans by BH-XT1 on K-562 XT1 KO cells as assessed by in-gel fluorescence. Cells stably expressing WT- or BH-XT1 (a) and WT- or BH-XT2 (b) or non-transfected were fed with compounds in the indicated concentrations in µM before on-cell CuAAC and in-gel fluorescence. Data are from one out of n = 3 independent replicates. Purple enzyme represents BH and green represent WT.

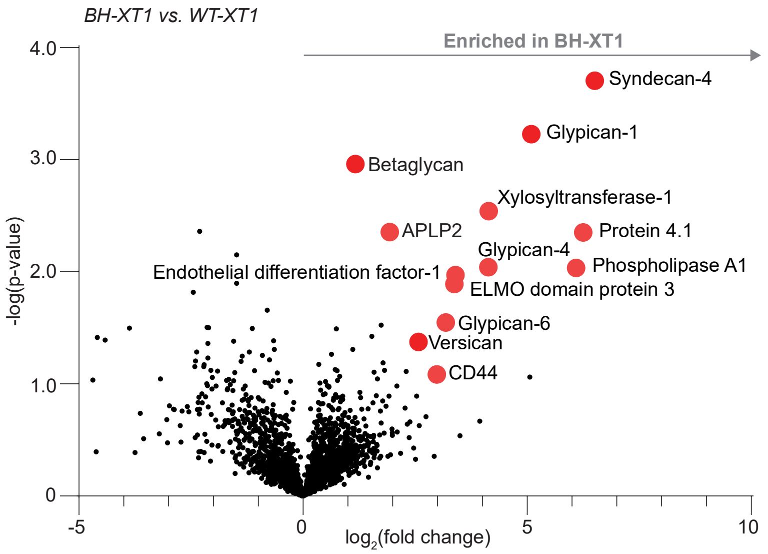

Supplementary Fig. 9: Volcano plots of individual proteins enriched by in differential MS experiments of secretome from BH- vs. WT-XT1-expressing pgsA-745 cells fed with compound 1 (250 μM) from n = 3 independent replicates.

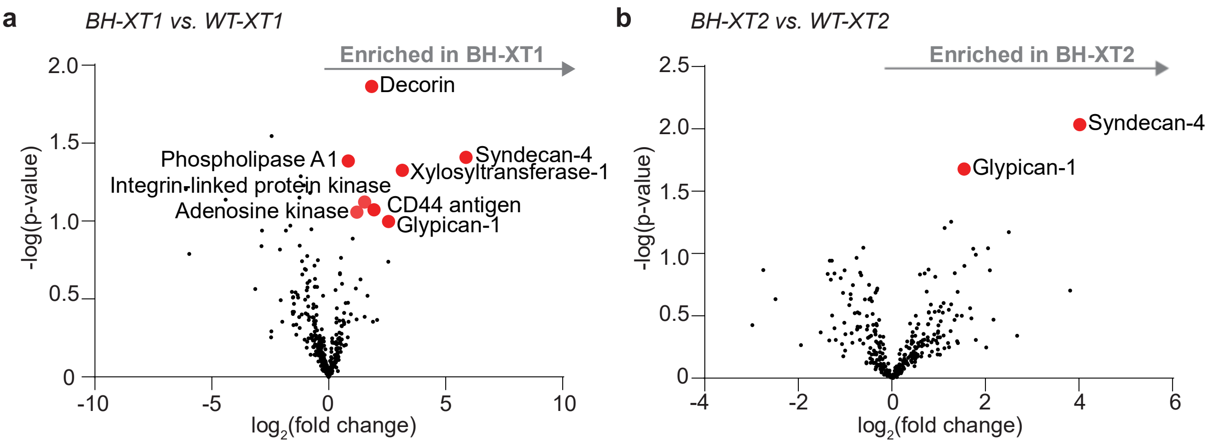

Supplementary Fig. 10: Volcano plots of individual proteins enriched by in differential MS experiments of secretome from BH- vs. WT-XT1/2-expressing pgsA-745 cells fed with compound **1** (50 µM) from n = 4 independent replicates.

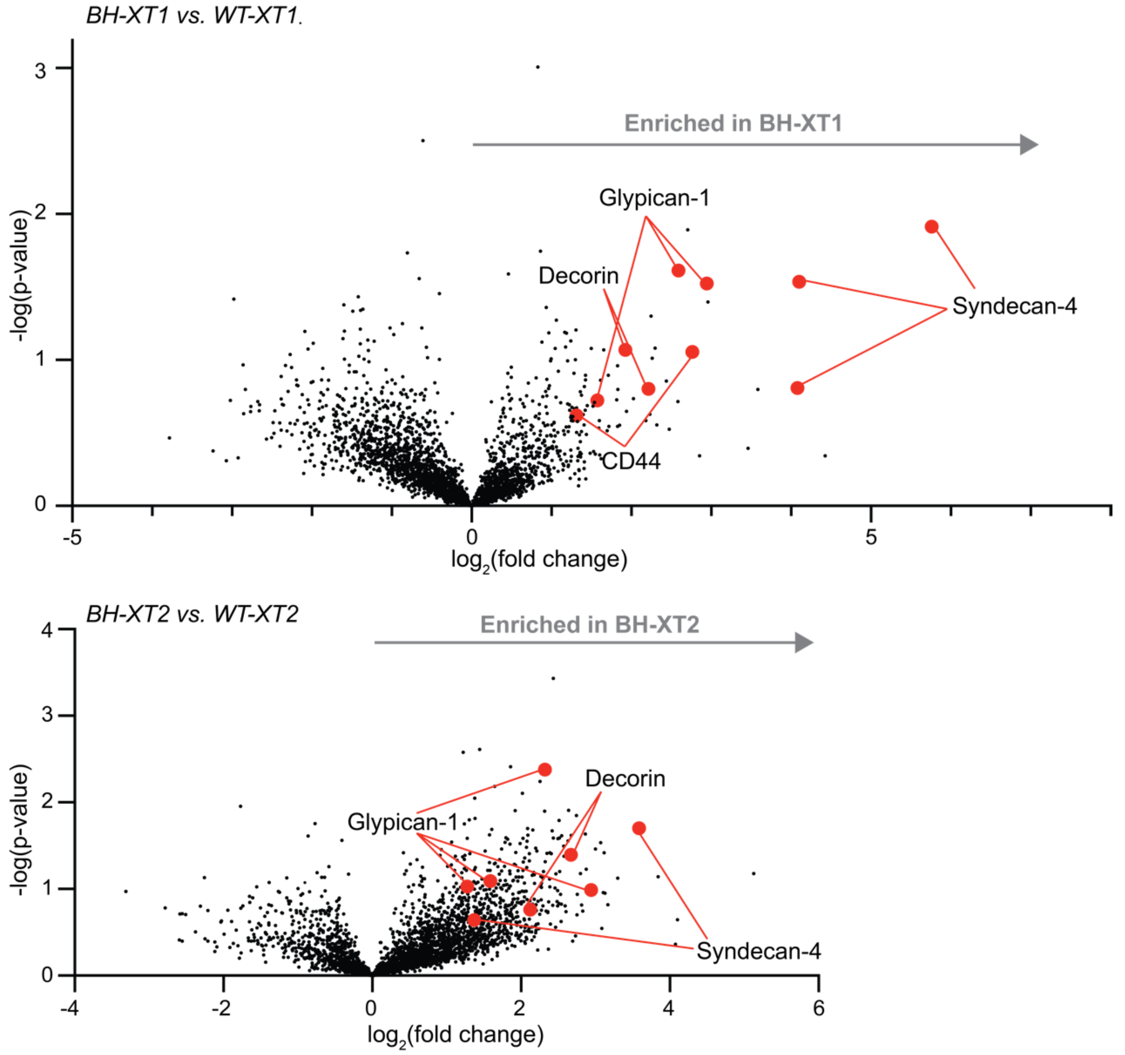

Supplementary Fig. 11: Volcano plots of individual peptides enriched by in differential MS experiments of secretome from BH- vs. WT-XT1/2-expressing pgsA-745 cells fed with compound **1** (50 µM) from n = 3 independent replicates.

Supplementary Table 1: Identified, enriched peptides from secretome samples in BH-XT1 and BH-XT2. Samples were processed from three independent replicates and analysis performed in Perseus according to Supplementary Fig. 10. Only one charge state per peptide is shown. n/a = not analyzed.

		BH-XT1 vs. WT-XT1			BH-XT2 vs. WT-XT2		
	**Peptide sequence**	**charge**	**-log(p value)**	**log2(fold change)**	**charge**	**-log(p value)**	**log2(fold change)**
**Syndecan-4**	EVEQNEVIPK	+2	1.9132256	5.76043749	+2	1.70032027	3.58913064
	VSPSEVDNDISNK	+2	0.80799931	4.0779624	+2	0.64023339	1.36967818
	RVSPSEVDNDISNK	+3	1.53655427	4.10091321	+3	0.59440457	1.77871513
	ETEVIDHQHFLEGR	+3	0.5158269	1.69561354	+3	0.35888786	0.94391918
**Glypican-1**	LALQEKPPTGSLEK	+3	0.28764139	1.1448005	+3	2.37743205	2.32337936
	SFVQGLGVASDVVR	+2	1.61215323	2.58823967	+2	1.09004828	1.58631134
	GCLANQADLDAEWR	+2	0.60544997	1.24314292	+2	1.02892665	1.27857939
	VAQVPLAPECSR	+2	1.5246964	2.94538943	+2	0.98795528	2.9424003
**Decorin**	NLHTLILVNNK	+3	0.79836121	1.82946014	+3	1.39351802	2.67002153
	ISSGAFTPLVK	+2	1.06943501	1.92464415	+2	0.54719266	1.40408516
	SSGIESGAFQGMK	+2	0.11169496	0.54547246	+2	0.49434041	1.94164133
**CD44**	YAGVFHVEK	+2	1.056109	2.76455148	+2	0.27005457	-0.9179918
	YGFIEGQVVIPR	+2	0.72334206	1.57499822	+2	0.08397354	-0.3173734

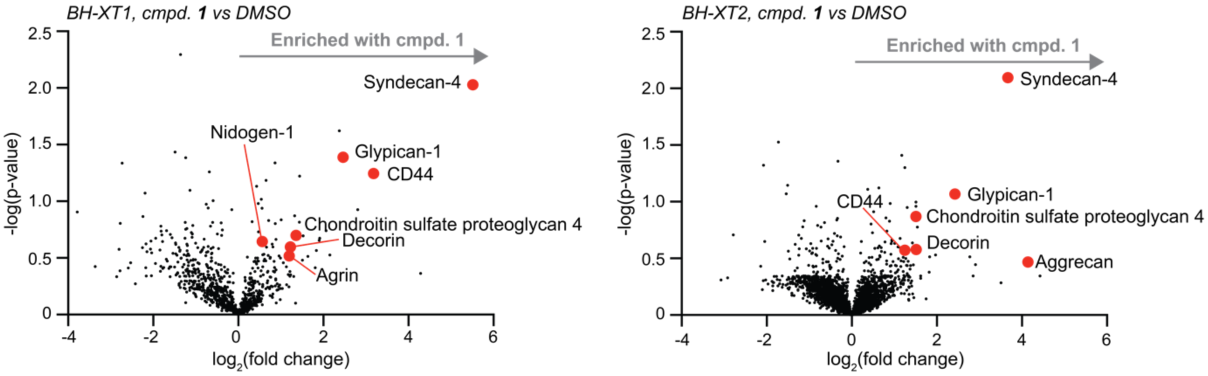

Supplementary Fig. 12: Volcano plots of proteoglycans enriched by in differential MS experiments of secretome from BH-XT1/2-expressing pgsA-745 cells fed with compound **1** (50 µM) or DMSO from n = 3 independent replicates.

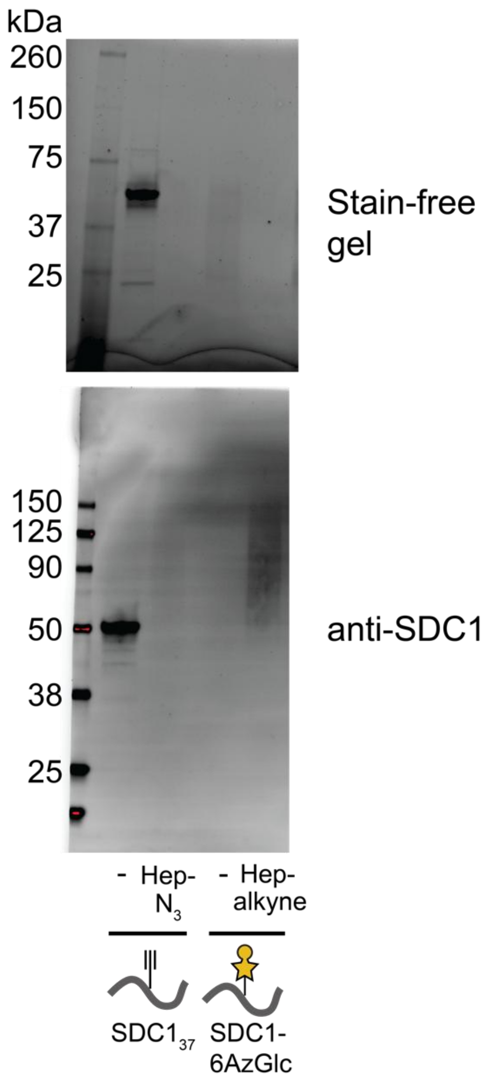

Supplementary Fig. 13: Generation of SDC1-heparin conjugates. Recombinant $SDC1_{37}$ and SDC1 were expressed and purified as described,[1] and incubated with clickable heparin under CuAAC conditions. Conjugates were analysed by stain-free gel and Western Blot with anti-SDC1 detection. Data are from one experiment.

Supplementary Table 2. Primers used for generation of XT1 mutants. Primers were from Merck KGaA (Darmstadt, Germany)

Primer names	Sequence
XT1 W392N rev	5'-ggaggctggctcctccgttgatggtggccattctc-3'
XT1 W392N for	5'-gagaatggccaccatcaacggaggagccagcctcc-3'
XT1 W392V rev	5'-aggctggctcctcccacgatggtggccattc-3'
XT1 W392V for	5'-gaatggccaccatcgtgggaggagccagcct-3'
XT1 W392H rev	5'-ggaggctggctcctccgtggatggtggccattctc-3'
XT1 W392H for	5'-gagaatggccaccatccacggaggagccagcctcc-3'
XT1 W392G rev	5'-ggctggctcctccgccgatggtggccatt-3'
XT1 W392G for	5'-aatggccaccatcggcggaggagccagcc-3'
XT1 L525A rev	5'-gaaggactcagcaggaagggcggtgtaggagtagaactg-3'
XT1 L525A for	5'-cagttctactcctacaccgcccttcctgctgagtccttc-3'
XT1 L525G rev	5'-gaaggactcagcaggaaggccggtgtaggagtagaactg-3'
XT1 L525G for	5'-cagttctactcctacaccggccttcctgctgagtccttc-3'
XT1 L526A rev	5'-ggaagaaggactcagcaggggccagggtgtaggagtagaac-3'
XT1 L526A for	5'-gttctactcctacaccctggcccctgctgagtccttcttcc-3'
XT1 L526V rev	5'-agaaggactcagcaggcaccagggtgtaggagtag-3'
XT1 L526V for	5'-ctactcctacaccctggtgcctgctgagtccttct-3'
XT1 L526G rev	5'-ggaagaaggactcagcagggcccagggtgtaggagtagaac-3'
XT1 L526G for	5'-gttctactcctacaccctgggccctgctgagtccttcttcc-3'
XT1 L525A L526A rev	5'-gtatggaagaaggactcagcaggagcggcggtgtaggagtagaactgtttca-3'
XT1 L525A L526A for	5'-tgaaacagttctactcctacaccgccgctcctgctgagtccttcttccatac-3'
XT1 L525A L526V rev	5'-gtatggaagaaggactcagcaggcacggcggtgtaggagtagaactgtttca-3'
XT1 L525A L526V for	5'-tgaaacagttctactcctacaccgccgtgcctgctgagtccttcttccatac-3'
XT1 L525A L526H rev	5'-gtatggaagaaggactcagcagggtgggcggtgtaggagtagaactgtttca-3'
XT1 L525A L526H for	5'-tgaaacagttctactcctacaccgcccaccctgctgagtccttcttccatac-3'
XT1 L525A L526G rev	5'-ccgtatggaagaaggactcagcagggccggcggtgtaggagtagaactgtttcatc-3'
XT1 L525A L526G for	5'-gatgaaacagttctactcctacaccgccggccctgctgagtccttcttccatacgg-3'
XT1 W392A L526A rev	Use XT1 L526A rev on XT1 W392A pCEP plasmid
XT1 W392A L526A for	Use XT1 L526A for on XT1 W392A pCEP plasmid
XT1 pOPING Infusion rev	5'-gtgatggtgatgtttaaactcacctgagccggccat-3'
XT1 pOPING Infusion for	5'-gcgtagctgaaaccggtaccatgagggcctggatctt-3'

Supplementary Table 3. siRNA locations used for SDC1 knock-down.

DsiRNA	Cross-reacting transcript	Location	Exon
1	NM_002997	3' UTR	5
	NM_001006946	3' UTR	6
2	NM_001006946	CDS	4
	NM_002997	CDS	3
3	NM_002997	CDS	3
	NM_001006946	CDS	4

Supplementary note

All chemicals were from commercial vendors. (N-[(*S*)-(2,3,4,5,6-Pentafluorophenoxy)phenoxyphosphinyl]-L-alanine 1-methylethyl ester was purchased from Biosynth (Staad, Switzerland). Anhydrous THF was obtained by passing solvent through activated alumina columns and dispensed from a PureSolv MD ASNA solvent purification system and stored over 4 Å molecular sieves. Unless otherwise stated, all reactions were conducted using anhydrous solvents, under an atmosphere of N_2 which was passed through a Drierite® drying column. HRMS (ESI, NSI) were obtained on Agilent 6530 Q-TOF, LQT Orbitrap XL1 or Waters (Xevo, G2-XS TOF or G2-S ASAP) Micromass LCT spectrometers using a methanol mobile phase in positive/ negative ionisation modes as appropriate. Analytical thin layer chromatography (TLC) was carried out on pre-coated 0.25 mm Merck KgaA 60 F254 silica gel plates. HPLC was performed using an Agilent 1260 Infinity II preparative HPLC system equipped with a variable wavelength detector and a fraction collector, on a reverse phase column (Polaris 180 Å C18-A. 21.2 × 250 mm, 5 µm) to achieve a purity level >95% and to determine percent purity. HPLC method: Polaris 180 Å C18-A. 21.2 × 250 mm, 5 µm, Solvent A: acetonitrile, solvent B: water, a gradient elution from 5% to 95% B over 20 mins, held for 7 mins, elution reversed to 5%B over 2 mins then held for 7 mins. Flow rate 3mL/min and UV detection at 254nm. Visualisation was achieved using UV detection at 254 nm. Visualisation was by adsorption of UV light, thermal development or thermal development after dipping in a methanolic solution of sulfuric acid (5% v/v). Column chromatography was carried out on 40–63 µm silica gel (Sigma-Aldrich) under a positive pressure of compressed air. NMR spectra were recorded on an Avance 400 spectrometer (Billerica, US). The chemical shift data for each signal are given as δ in units of parts per million (ppm) relative to tetramethylsilane, where δ = 0.00 ppm and the standardised deuterated solvent peak ($CDCl_3$ δ = 7.26 ppm). The number of protons (n) for a given resonance is indicated by nH. The multiplicity of each signal is indicated by s (singlet), br s (broad singlet), d (doublet), t (triplet), q (quartet), p (pentet), sep (septet), dd (doublet of doublets), ddd (doublet of doublet of doublets), dddd (doublet of doublet of doublet of doublets), dt (doublet of triplets), tt (triplet of triplets), dqd (doublet of quartets of doublets) or m (multiplet). Coupling constants (*J*) are quoted in Hz and calculated to the nearest 0.1 Hz. Additional experiments were performed to aid complete characterisation including: ^1H-^1H homonuclear correlation spectroscopy (COSY), ^1H-^{13}C heteronuclear correlation spectroscopy (HSQC) which was either proton coupled or proton decoupled, ^{31}P proton coupled and ^{31}P proton decoupled. Assignment of ^1H and ^{13}C atoms in NMR follows standard pyranose ring numbering.

Compound **4** was made from commercially available 6-azido-6-deoxyglucose as described before.[1]

(N-(*R*)-[phenoxyphosphinyl]-L-alanine 1-methylethyl ester)-2,3,4-tri-O-acetyl-6-azido-6-deoxy-α-D-glucopyranoside (1) and (N-(*R*)-[phenoxyphosphinyl]-L-alanine 1-methylethyl ester)-2,3,4-tri-O-acetyl-6-azido-6-deoxy-β-D-glucopyranoside (2): Compound **4**[2] (180 mg, 0.54 mmol) was dissolved in anhydrous THF (3.2 mL) and cooled to -78 °C. A solution of 2 M LDA in THF (270 µL, 0.54 mmol) was added and the reaction was stirred for 15 mins before addition of N-[(*S*)-(2,3,4,5,6-Pentafluorophenoxy)phenoxyphosphinyl]-L-alanine 1-methylethyl ester (300 mg, 0.57 mmol) as a solution in THF (2.3 mL). The reaction was kept at -70 °C for 40 mins, then quenched by the addition of methanol (300 µL). After stirring for 5 mins, the solution was directly dry loaded on to silica for column chromatography using a gradient elution of 10% - 30% EtOAc in hexane with 1% Et_3N. The alpha anomer **1** was isolated (197 mg, 60% as a waxy white solid). Successive purifications of the beta anomer did not produce anomerically pure material and so preparative TLC was performed using 60% ethyl acetate in hexane with 1% Et_3N, yielding the beta anomer **2** (32 mg, 9.8%) as an oil with approx. 4% of the alpha-anomer **1** as a remaining impurity,

as assessed by NMR. Alpha **1**: Rf = 0.61 (60% EtOAc in hexane with 1% Et_3N) ^{1}H NMR (400 MHz, $CDCl_3$) δ 7.37 - 7.32 (m, 2H ArH), 7.27 - 7.24 (m, 2H, ArH), 7.22 - 7.16 (m, 1H, ArH), 5.97 - 5.94 (dd, 1H, H1, *J* = 7.4, 3.4 Hz), 5.45 (t, 1H, H3, *J* = 10.3, 9.4 Hz), 5.11- 5.02 (m, 2H, H4, O*CH*(CH_3)$_2$), 5.01 - 4.98 (ddd, 1H, H2, *J* = 10.3, 3.4, 2.5 Hz), 4.08 - 4.00 (m, 2H, H5, NHC*H*CH_3), 3.83 - 3.77 (m, 1H, NH), 3.30 - 3.26 (dd, 1H, H6a, *J* = 13.5, 2.9 Hz), 3.22 - 3.18 (dd, 1H, H6b, *J* = 13.5, 4.9 Hz) , 2.06 (s, 3H, CH_3), 2.04 (s, 3H, CH_3), 2.02 (s, 3H, CH_3), 1.45 - 1.43 (d,3H, NHCHC*H*$_3$, *J* = 7.0 Hz), 1.26 (d, 3H, OCH(*C*H_3)$_2$, *J* = 3.9 Hz), 1.25 (d, 3H, OCH(*C*H_3)$_2$, *J* = 3.9 Hz) .^{13}C NMR (101 MHz, $CDCl_3$) δ 172.8 (d, *C*OOCH(CH_3)$_2$, *J* = 9.6 Hz), 170.2, 169.9, 169.5 (C=O), 150.62 (d, qArC, *J* = 7.0 Hz) , 130.0 (ArC), 125.3 (d, ArC, *J* = 1.3 Hz) , 120.3 (d, ArC, *J*= 5.0 Hz), 93.40 (d, C1, *J* = 5.6 Hz), 70.4 (C5), 69.9 (C2, *J* = 7.5 Hz), 69.7 (O*C*H(CH_3)$_2$), 69.5 (C3), 68.9 (C4), 50.7 (C6), 50.4 (d, N*C*HCH_3, *J* = 2.4 Hz), 21.8 (d, CH(*C*H_3)$_2$, *J* = 3.2 Hz), 21.3, (d, NHCH*C*H_3, *J* = 3.7 Hz), 20.8 (CH_3), 20.7 (CH_3), 20.7 (CH_3). ^{1}H-^{13}C-HSQC coupled: $^{1}J_{C\text{-}H}$ 180Hz. ^{31}P NMR (162 MHz, $CDCl_3$) δ 1.11 - 0.94 (q, *J* = 8.8 Hz). HRMS $C_{24}H_{34}O_{12}N_4P_1$ [M+H$^+$] calc 601.1905 found: 601.1913 $[\alpha]_D$ = +91.9 (c = 1.0, $CHCl_3$), Reverse phase C18 HPLC retention time: 21.874 mins, 95.5% purity Beta **2**: Rf = 0.5 (60% ethyl acetate in hexane with 1% Et_3N) ^{1}H NMR (400 MHz, $CDCl_3$) δ 7.33 - 7.29 (app t, 2H, ArH), 7.20 - 7.14 (m, 3H, ArH), 5.37 - 5.33 (t, H1, 1H, *J* = 7.8 Hz), 5.19 - 5.14 (m, 1H, H3), 5.09 - 5.02 (m, 3H, H2, H4, OC*H*(CH_3)$_2$) , 4.08 - 4.00 (m, 1H, NHC*H*CH_3), 3.84 (dd, 1H, NH, *J* = 12.1, 8.6 Hz), 3.76 (ddd, 1H, H5, *J* = 9.9, 5.4, 2.8 Hz, 1H), 3.42 (dd, 1H, H6a, *J* = 13.5, 2.9 Hz), 3.33 (dd, 1H, H6b, *J* = 13.5, 5.4 Hz), 2.03 (s, 3H, COC*H*$_3$), 1.97 (s, 3H, COC*H*$_3$), 1.74 (s, 3H, COC*H*$_3$),1.42 (d, 3H, CH_3 *J* = 7.1 Hz,), 1.26 (d, 3H, OCH(C*H*$_3$)$_2$, *J* = 6.3 Hz), 1.24 (d, 3H, OCH(C*H*$_3$)$_2$, *J* = 6.2 Hz).^{13}C NMR (101 MHz, $CDCl_3$) δ 172.7 (d, *C*OOCH(CH_3)$_2$, *J* = 10.1 Hz), 170.1, 169.5, 169.3 (CO), 150.6 (d, qArC, *J* = 7.3 Hz), 129.8 (ArCH), 125.3 (ArCH), 120.5 (d, ArCH, *J* = 4.8 Hz), 95.8 (d, C1, *J* = 4.5 Hz), 73.7 (C5), 72.4 (d, C3, *J* = 1.8 Hz), 71.0 (d, C2, *J* = 8.9 Hz), 69.5, 68.9 (C4, *C*H(CH_3)$_2$), 50.8 (C6), 50.4 (d, NHCH, *J* = 2.2 Hz), 21.8 (OCH(*C*H_3)$_2$), 20.9 (d, NHCH*C*H_3, *J* = 3.6 Hz), 20.7, 20.6, 20.5 (CO*C*H_3). ^{1}H-^{13}C-HSQC coupled: $^{1}J_{C\text{-}H}$ 168Hz. ^{31}P NMR (162 MHz, $CDCl_3$) δ 1.38 - 1.20 (dt, *J* = 8.1Hz). HRMS: $C_{24}H_{34}O_{12}N_4P_1$ [M+H$^+$] calc 601.1905 found: 601.1909. $[\alpha]_D$ = +4 (c = 1.0, $CHCl_3$). Reverse phase C18 HPLC retention time: 21.858 mins, 98.4% purity.

Supplementary references

1. Mehta, S., Meldal, M., Ferro, V., Duus, J. & Bock, K. Internally quenched fluorogenic, α-helical dimeric peptides and glycopeptides for the evaluation of the effect of glycosylation on theconformation of peptides. *J. Chem. Soc. Perkin* **1,** 1365–1374 (1997).
2. Müller, A. *et al.* Synthesis of Bifunctional Azobenzene Glycoconjugates for Cysteine-Based Photosensitive Cross-Linking with Bioactive Peptides. *Chem. Eur. J.* **21**, 13723–13731 (2015).

Supplementary Figures for Spectra and Chromatograms of Compounds

Supplementary Fig.14 ^{1}H NMR spectrum (0-9.5 ppm) of compound **1** (N-(*R*)-[phenoxyphosphinyl]-L-alanine 1-methylethyl ester)-2,3,4-tri-O-acetyl-6-azido-6-deoxy-α-D-glucopyranoside) in $CDCl_3$
Supplementary Fig.15 ^{1}H NMR spectrum (3-6 ppm) of **1** in $CDCl_3$
Supplementary Fig.16 ^{13}C NMR spectrum of **1** in $CDCl_3$
Supplementary Fig.17 ^{31}P NMR spectrum of **1**
Supplementary Fig.18 ^{31}P proton decoupled NMR spectrum of **1**
Supplementary Fig.19 ^{1}H -^{13}C HSQC NMR spectrum of **1**
Supplementary Fig.20 ^{1}H -^{13}C HSQC NMR spectrum of **1** highlighting $^1J_{C-H}$
Supplementary Fig.21 High-Resolution ESI Mass Spectrum of **1**
Supplementary Fig.22 HPLC chromatogram of **1**

Supplementary Fig.23 ^{1}H NMR spectrum (0-9.5 ppm) of compound **2** (N-(*R*)-[phenoxyphosphinyl]-L-alanine 1-methylethyl ester)-2,3,4-tri-O-acetyl-6-azido-6-deoxy-β-D-glucopyranoside) in $CDCl_3$
Supplementary Fig.24 ^{1}H NMR spectrum (3-6 ppm) of **2** in $CDCl_3$
Supplementary Fig.25 ^{13}C NMR spectrum of **2** in $CDCl_3$
Supplementary Fig.26 ^{31}P NMR spectrum of **2**
Supplementary Fig.27 ^{31}P proton decoupled NMR spectrum of **2**
Supplementary Fig.28 ^{1}H -^{13}C HSQC NMR spectrum of **2**
Supplementary Fig.29 ^{1}H -^{13}C HSQC NMR spectrum of **2** highlighting $^1J_{C-H}$
Supplementary Fig.30 High-Resolution ESI Mass Spectrum of **2**
Supplementary Fig.31 HPLC chromatogram of **2**

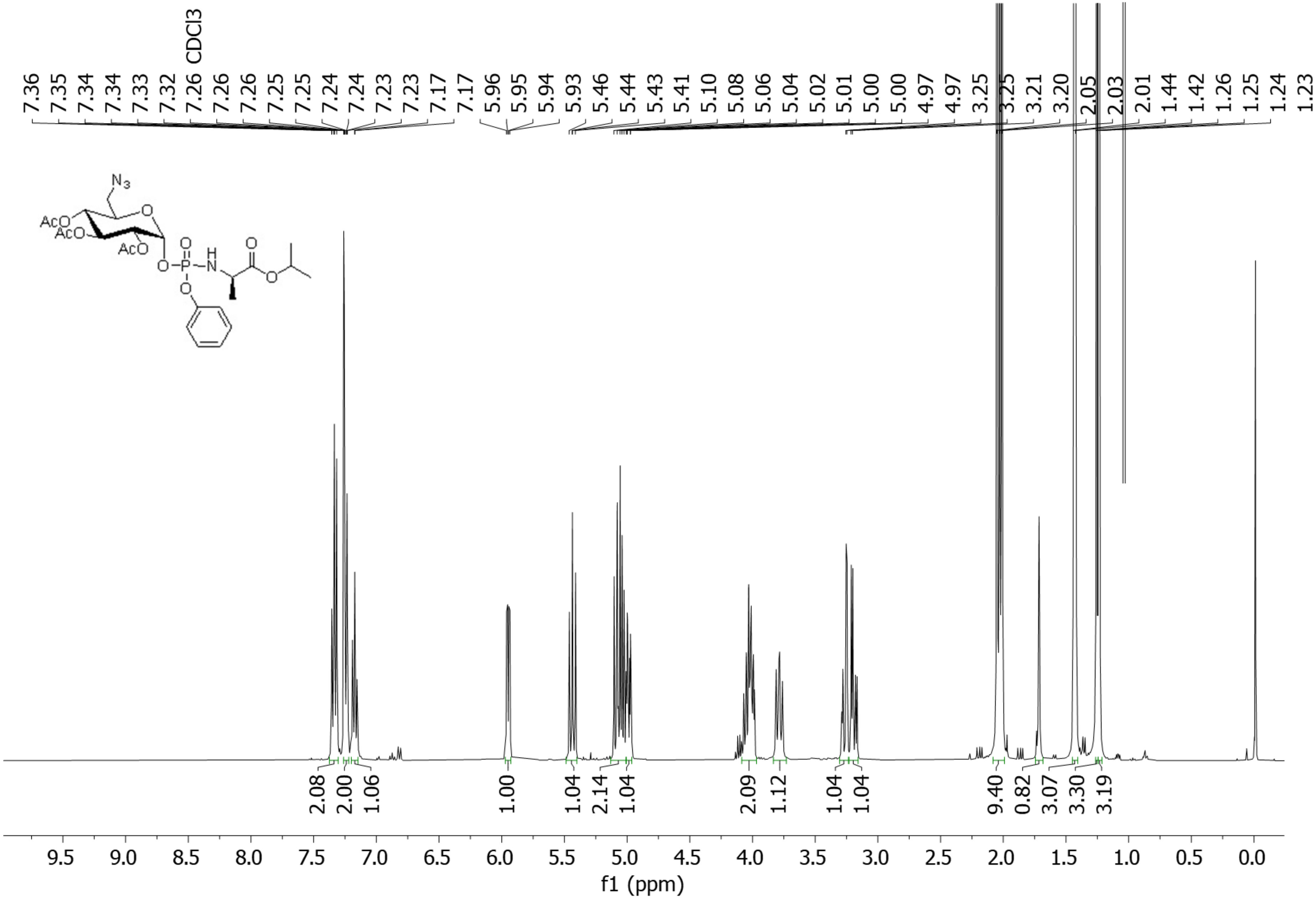

Supplementary Fig.14 ^{1}H NMR spectrum (0-9.5 ppm) of compound **1** (N-(*R*)-[phenoxyphosphinyl]-L-alanine 1-methylethyl ester)-2,3,4-tri-O-acetyl-6-azido-6-deoxy-α-D-glucopyranoside) in $CDCl_3$

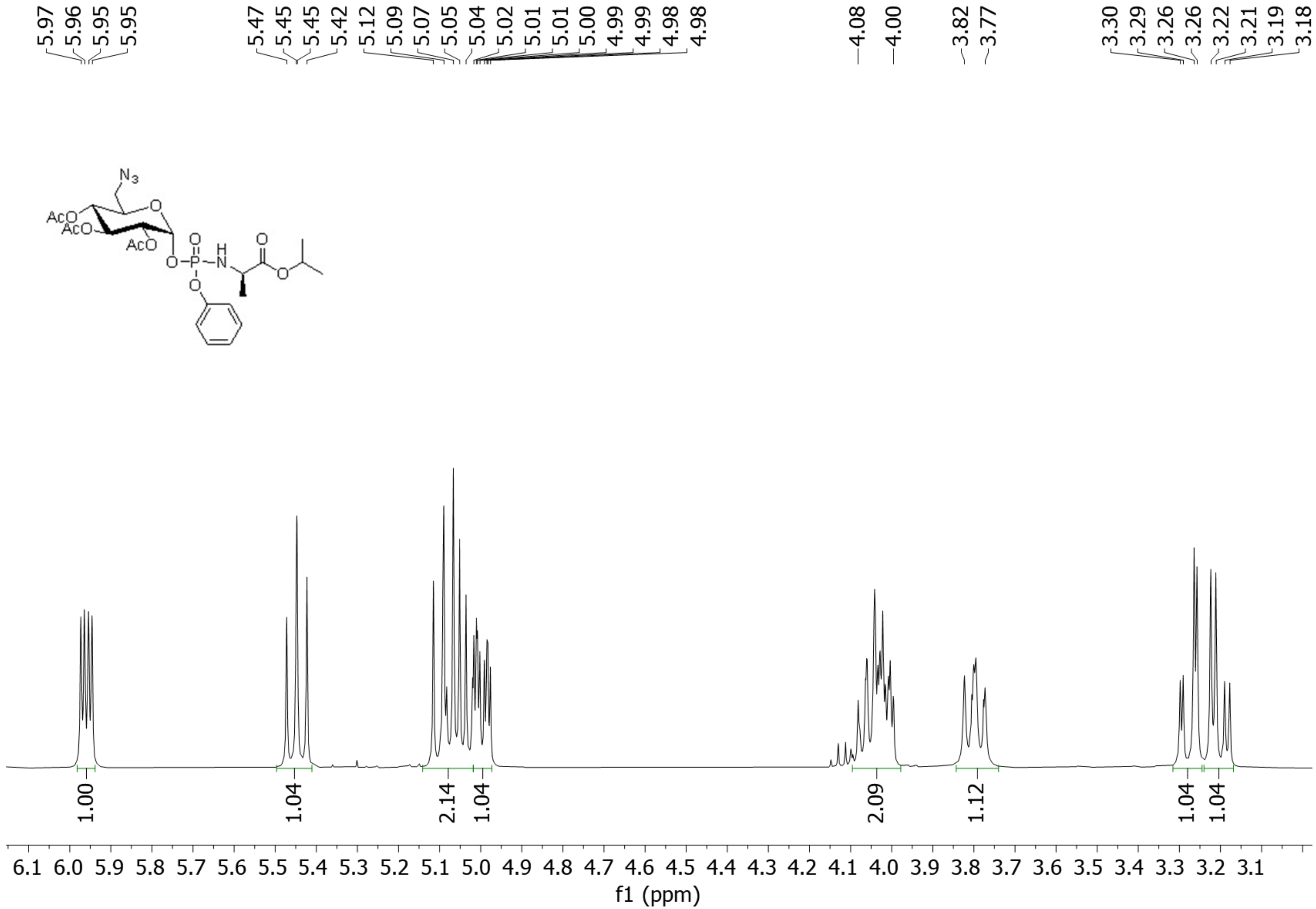

Supplementary Fig.15 ^{1}H NMR spectrum (3-6 ppm) of **1** in $CDCl_3$

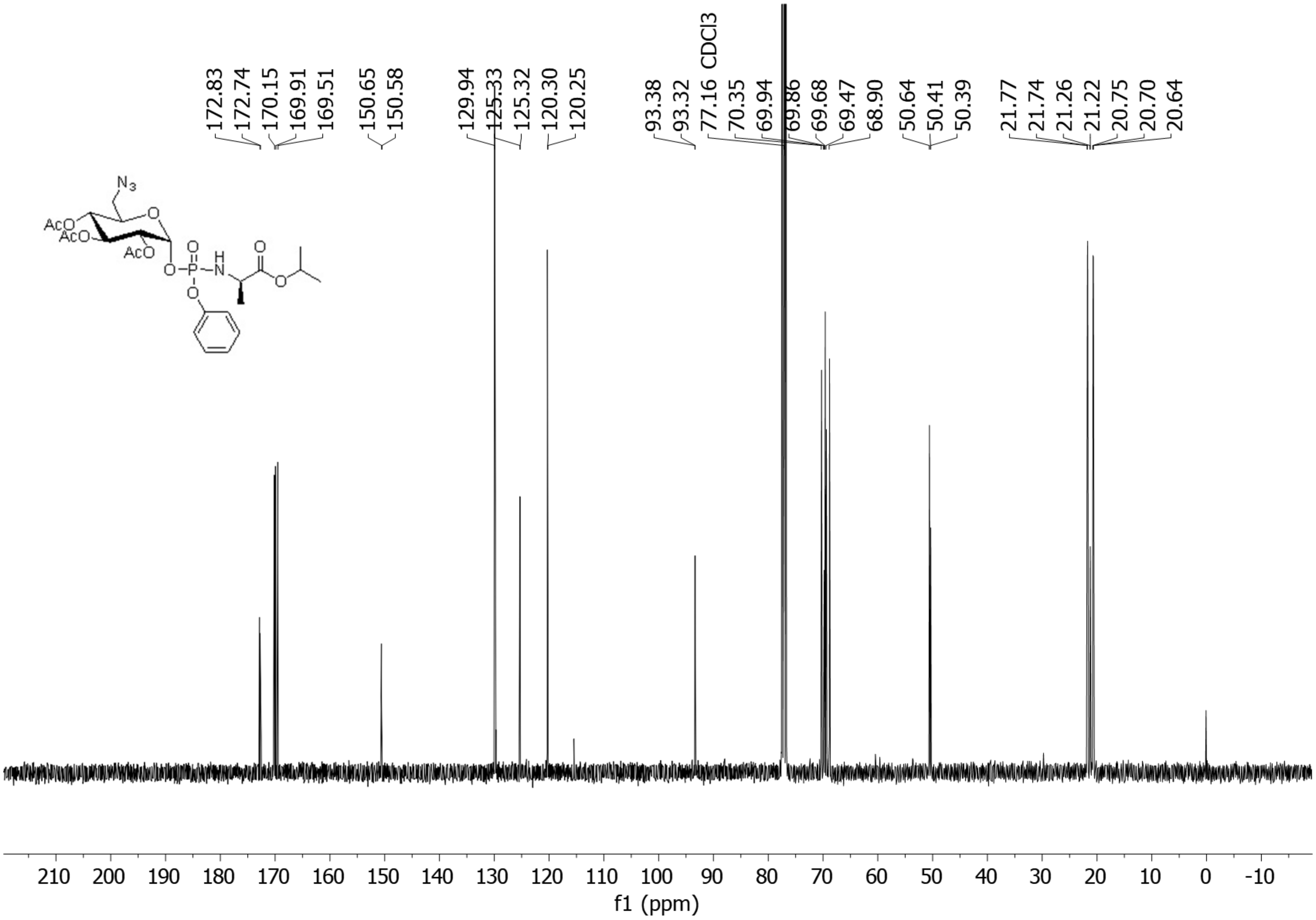

Supplementary Fig.16 ^{13}C NMR spectrum of **1** in $CDCl_3$

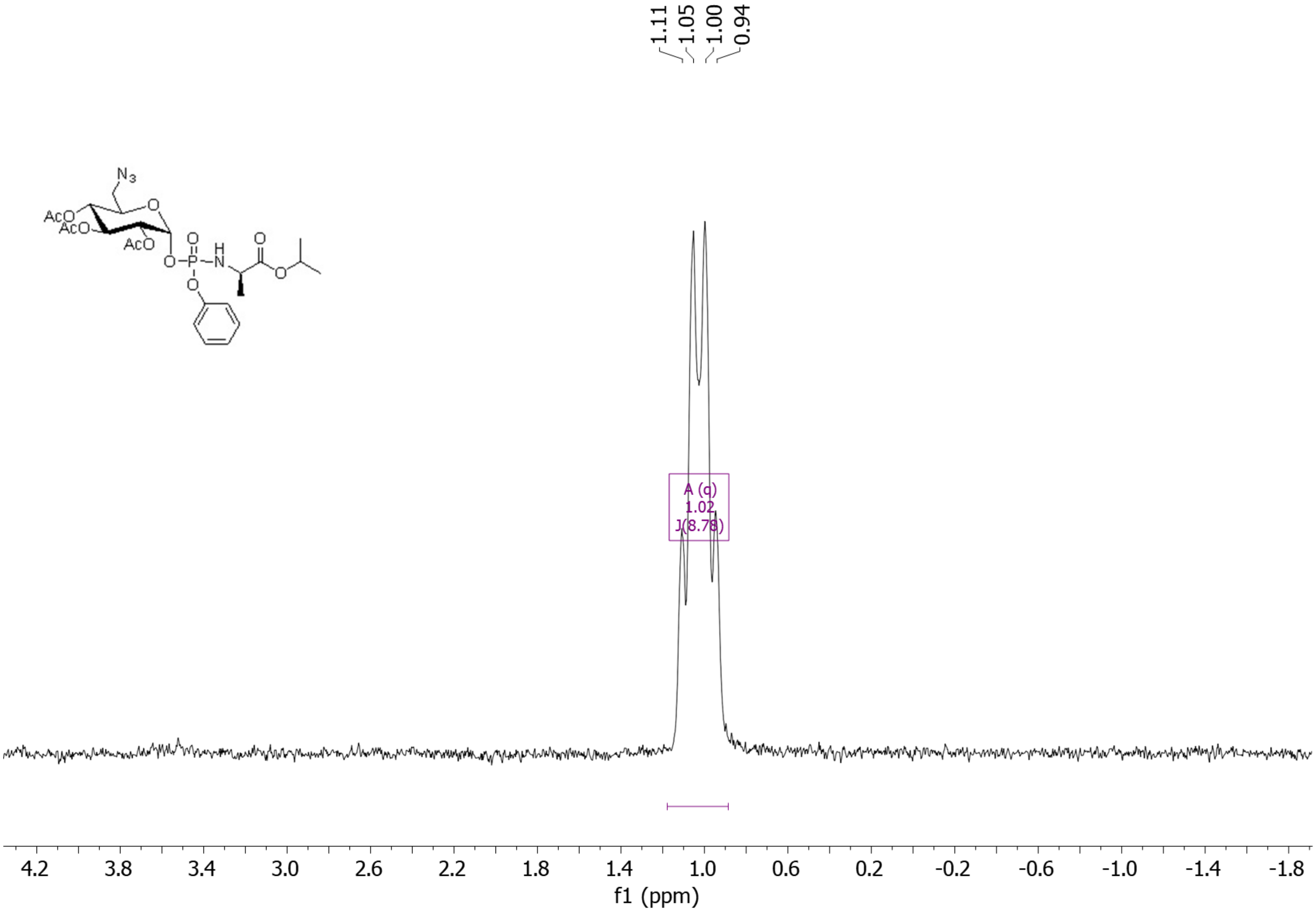

Supplementary Fig.17 ^{31}P NMR spectrum of **1**

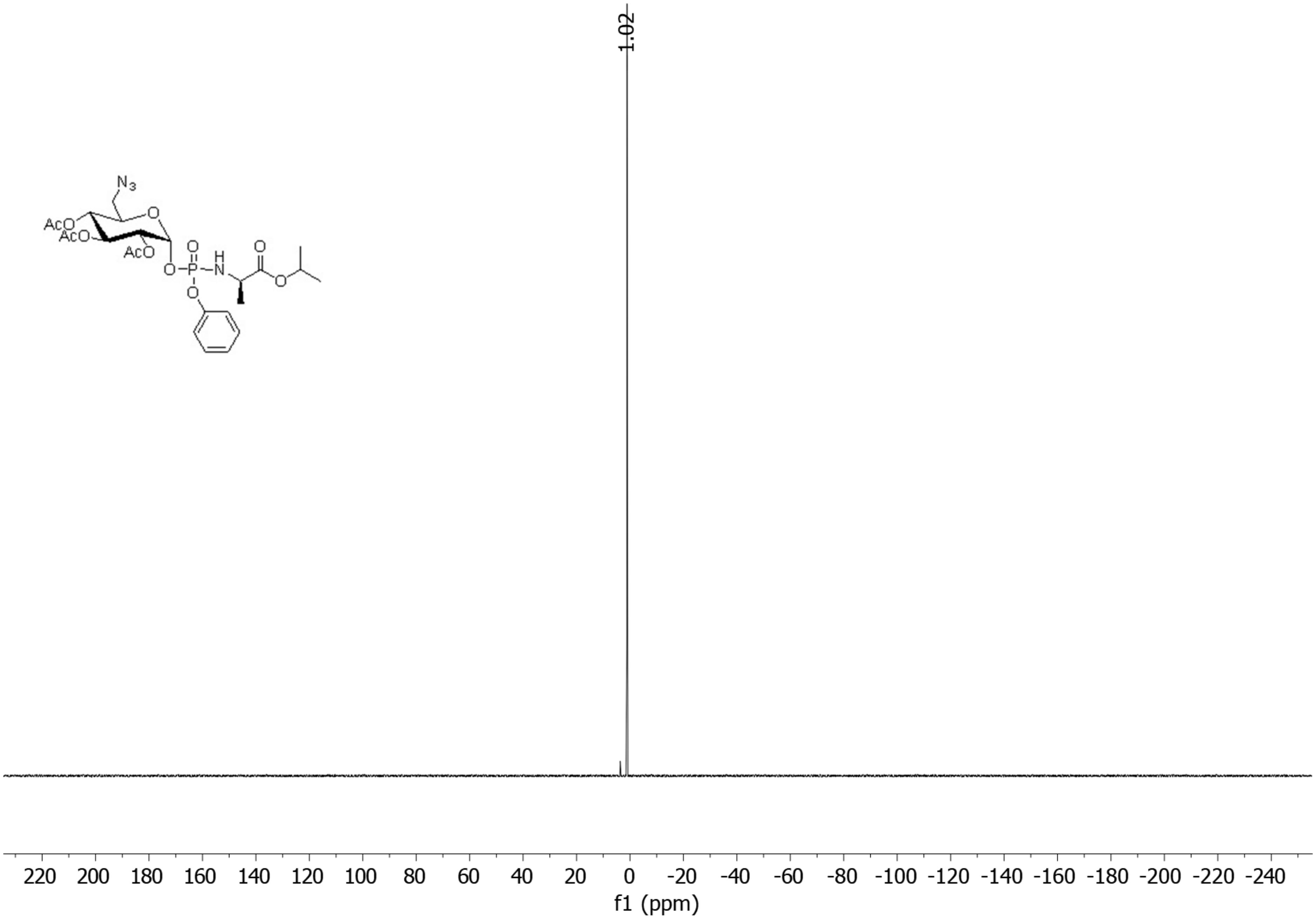

Supplementary Fig.18 Decoupled ^{31}P NMR spectrum of **1**

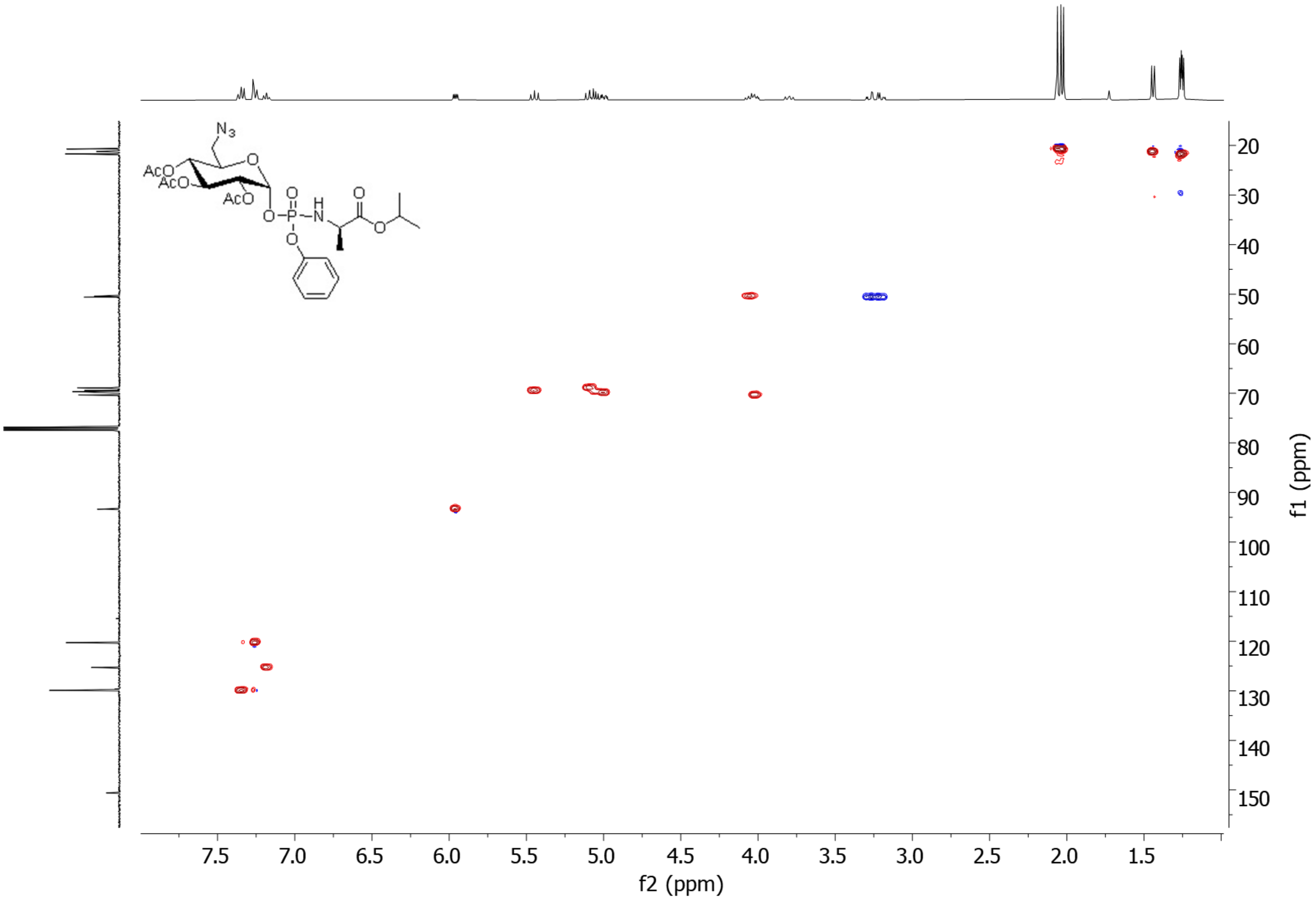

Supplementary Fig.19 ^{1}H -^{13}C HSQC NMR spectrum of **1**

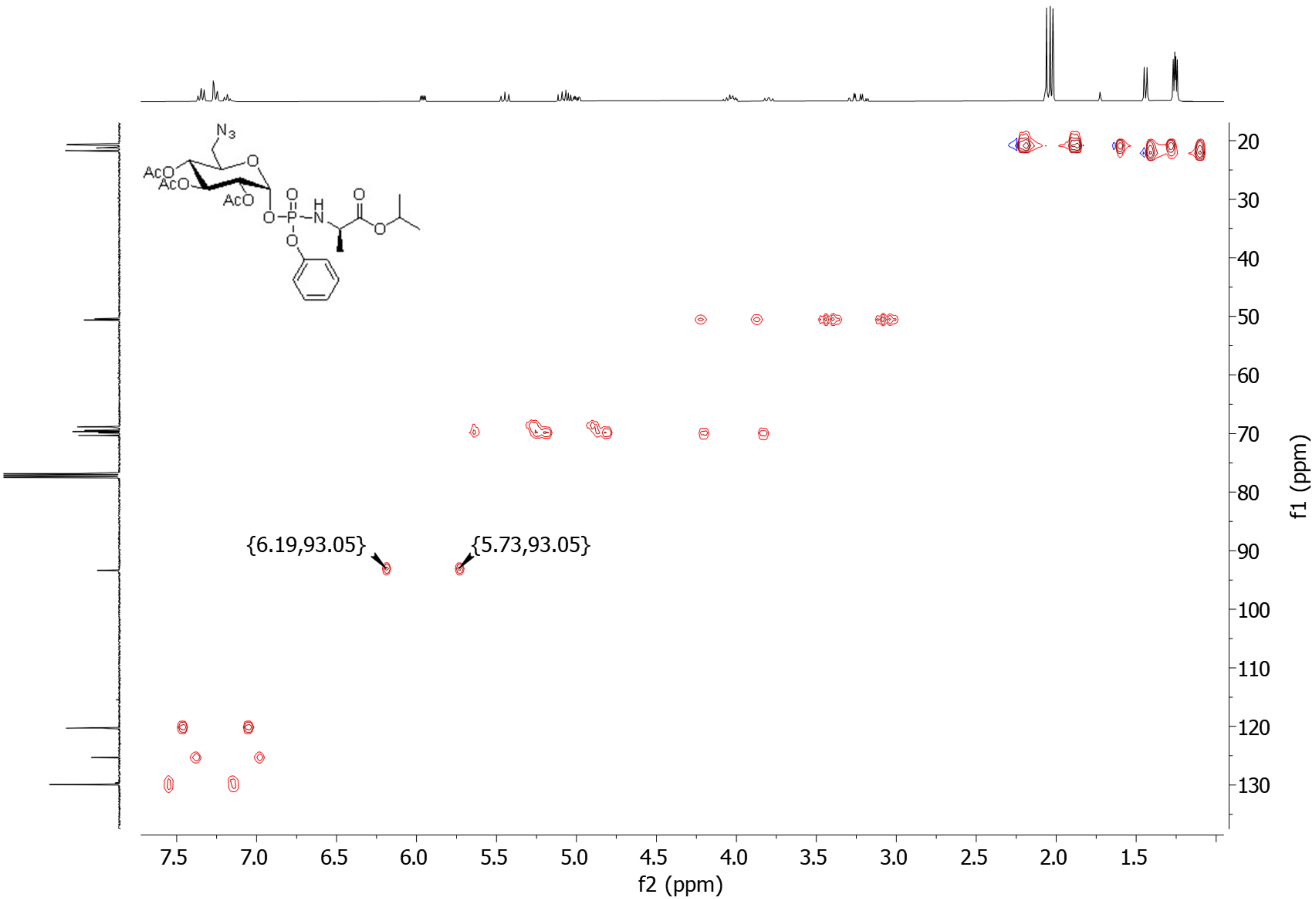

Supplementary Fig.20 ^{1}H -^{13}C HSQC NMR spectrum of **1** highlighting $^{1}J_{C\text{-}H}$

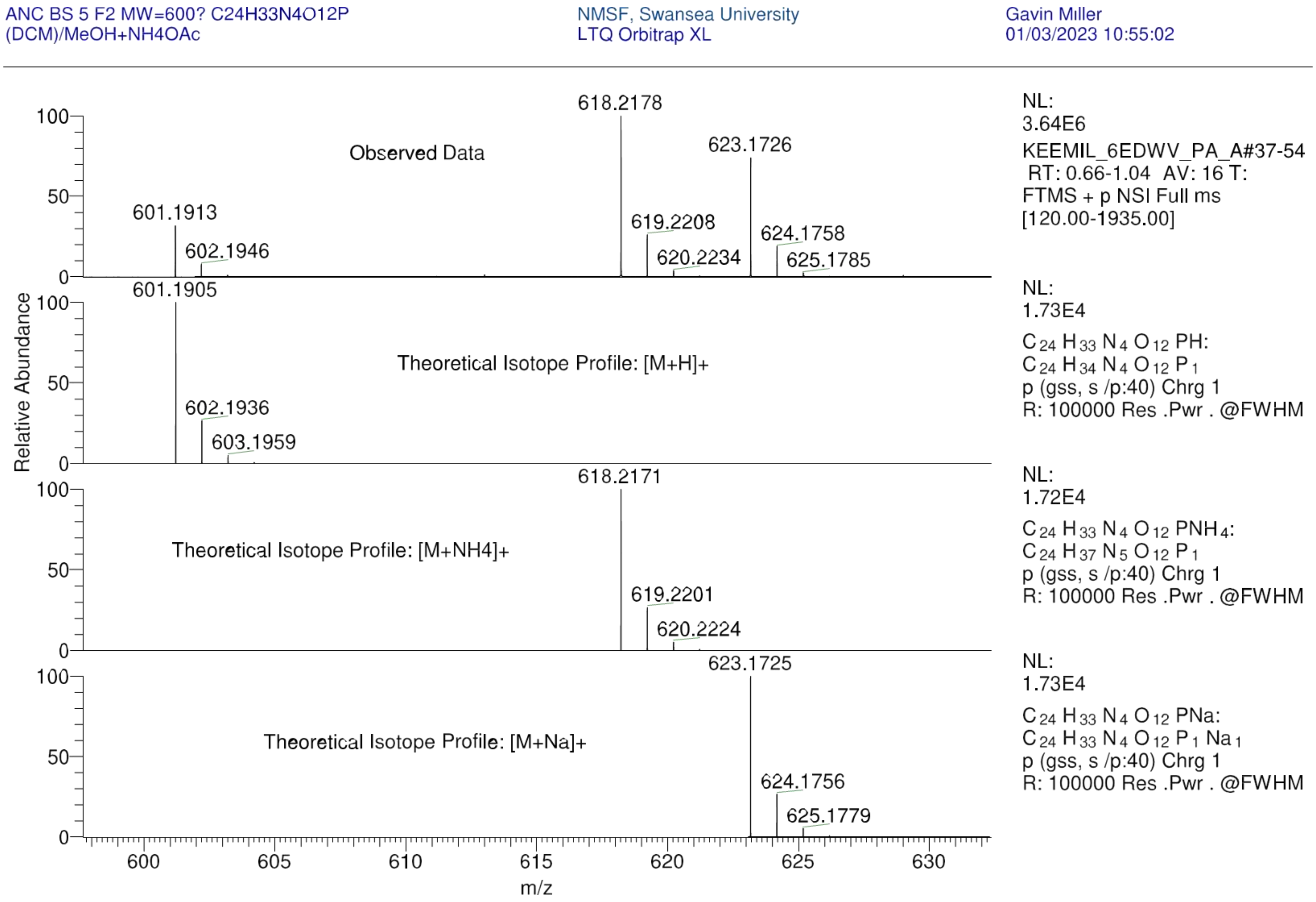

Supplementary Fig.21 High-Resolution ESI Mass Spectrum of **1**

Isotope:	Min. .. Max.		
14 N	0....16		
16 O	0....20		
12 C	0....100		
1 H	0....120		
23 Na	1....1		
31 P	0....2		
Tolerance Window:	+- 5.00 ppm		
Db/Ring Equiv:	-10.. 500	N-Rule:	Do not use
Fits:	500	Charge:	1

Mass	Theoretical Mass	Delta [ppm]	RDB	Composition
601.1913	601.1914	-0.1	-6.5	$C_{6} H_{39} O_{18} N_{10} P_{2}$
	601.1914	-0.1	28.0	$C_{39} H_{28} O_{2} N_{3} P_{1}$
	601.1911	0.4	32.5	$C_{43} H_{25} O_{2} N_{2}$
	601.1910	0.4	-2.0	$C_{10} H_{36} O_{18} N_{9} P_{1}$
	601.1916	-0.4	25.5	$C_{28} H_{21} O_{3} N_{14}$
	601.1910	0.4	3.5	$C_{9} H_{30} O_{13} N_{16} P_{1}$
	601.1916	-0.4	20.0	$C_{29} H_{27} O_{8} N_{7}$
	601.1916	-0.4	14.5	$C_{30} H_{33} O_{13}$
	601.1917	-0.6	23.5	$C_{35} H_{31} O_{2} N_{4} P_{2}$
	601.1908	0.8	6.0	$C_{20} H_{37} O_{12} N_{5} P_{2}$
	601.1908	0.8	11.5	$C_{19} H_{31} O_{7} N_{12} P_{2}$
	601.1907	0.9	2.5	$C_{14} H_{33} O_{18} N_{8}$
	601.1919	-0.9	21.0	$C_{24} H_{24} O_{3} N_{15} P_{1}$
	601.1907	1.0	8.0	$C_{13} H_{27} O_{13} N_{15}$
	601.1919	-1.0	15.5	$C_{25} H_{30} O_{8} N_{8} P_{1}$
	601.1919	-1.0	10.0	$C_{26} H_{36} O_{13} N_{1} P_{1}$
	601.1905	1.3	10.5	$C_{24} H_{34} O_{12} N_{4} P_{1}$
	601.1905	1.3	16.0	$C_{23} H_{28} O_{7} N_{11} P_{1}$
	601.1921	-1.3	7.5	$C_{15} H_{29} O_{14} N_{12}$
	601.1921	-1.3	2.0	$C_{16} H_{35} O_{19} N_{5}$
	601.1922	-1.5	16.5	$C_{20} H_{27} O_{3} N_{16} P_{2}$
	601.1922	-1.5	11.0	$C_{21} H_{33} O_{8} N_{9} P_{2}$
	601.1922	-1.5	5.5	$C_{22} H_{39} O_{13} N_{2} P_{2}$
	601.1903	1.6	18.5	$C_{34} H_{35} O_{6} P_{2}$
	601.1903	1.6	24.0	$C_{33} H_{29} O_{1} N_{7} P_{2}$
	601.1902	1.8	15.0	$C_{28} H_{31} O_{12} N_{3}$
	601.1902	1.8	20.5	$C_{27} H_{25} O_{7} N_{10}$
	601.1924	-1.8	3.0	$C_{11} H_{32} O_{14} N_{13} P_{1}$
	601.1924	-1.8	-2.5	$C_{12} H_{38} O_{19} N_{6} P_{1}$
	601.1900	2.1	28.5	$C_{37} H_{26} O_{1} N_{6} P_{1}$
	601.1900	2.1	-6.0	$C_{4} H_{37} O_{17} N_{13} P_{2}$

Supplementary Fig.21 High-Resolution ESI Mass Spectrum of **1**

```
Data File C:\Users\P...NC BS6 iii percent purity repeat 2023-10-03 14-46-55\alpha anomer.D
Sample Name: alpha anomer

    ====================================================================
    Acq. Operator    : SYSTEM                          Seq. Line :   1
    Sample Operator  : SYSTEM
    Acq. Instrument  : Prep LC                          Location :   P2-A-02
    Injection Date   : 10/3/2023 2:47:58 PM                  Inj :   1
                                                      Inj Volume : 200.000 µl
    Method           : C:\Users\Public\Documents\ChemStation\1\Data\Aisling\ANC BS6 iii percent
                       purity repeat 2023-10-03 14-46-55\ANC-BS-6iii Percent Purity.M (Sequence
                       Method)
    Last changed     : 10/3/2023 2:46:52 PM by SYSTEM
    Method Info      : Polaris 5 C15-A 250 x10mm SN 593740

    Additional Info : Peak(s) manually integrated
```

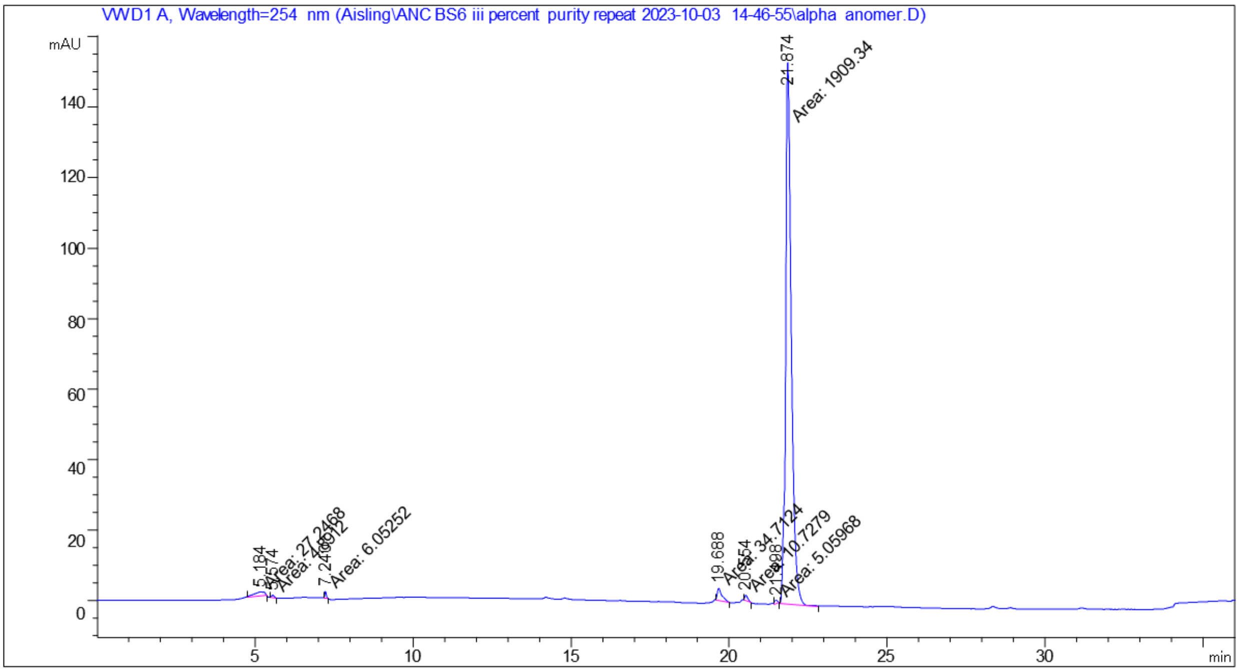


```
    =================================================================================
                               Fraction Information
    =================================================================================
    No Fractions found.
    =================================================================================
    =================================================================================
    ====================================================================
                             Area Percent Report
    ====================================================================


    Sorted By             :      Signal
    Multiplier            :      1.0000
    Dilution              :      1.0000
    Use Multiplier & Dilution Factor with ISTDs
```


Supplementary Fig.22 HPLC chromatogram of **1**

Signal 1: VWD1 A, Wavelength=254 nm

Peak #	RetTime [min]	Type	Width [min]	Area [mAU*s]	Height [mAU]	Area %
1	5.184	MM	0.3947	27.24684	1.15067	1.3637
2	5.574	MM	0.1148	4.89120	7.10091e-1	0.2448
3	7.240	MM	0.0557	6.05252	1.81123	0.3029
4	19.688	MM	0.1701	34.71244	3.40032	1.7373
5	20.554	MM	0.1191	10.72786	1.50101	0.5369
6	21.498	MM	0.1049	5.05968	8.03798e-1	0.2532
7	21.874	MM	0.2072	1909.33606	153.56323	95.5611
Totals :				1998.02659	162.94035	

*** End of Report ***

Supplementary Fig.22 HPLC chromatogram of **1**

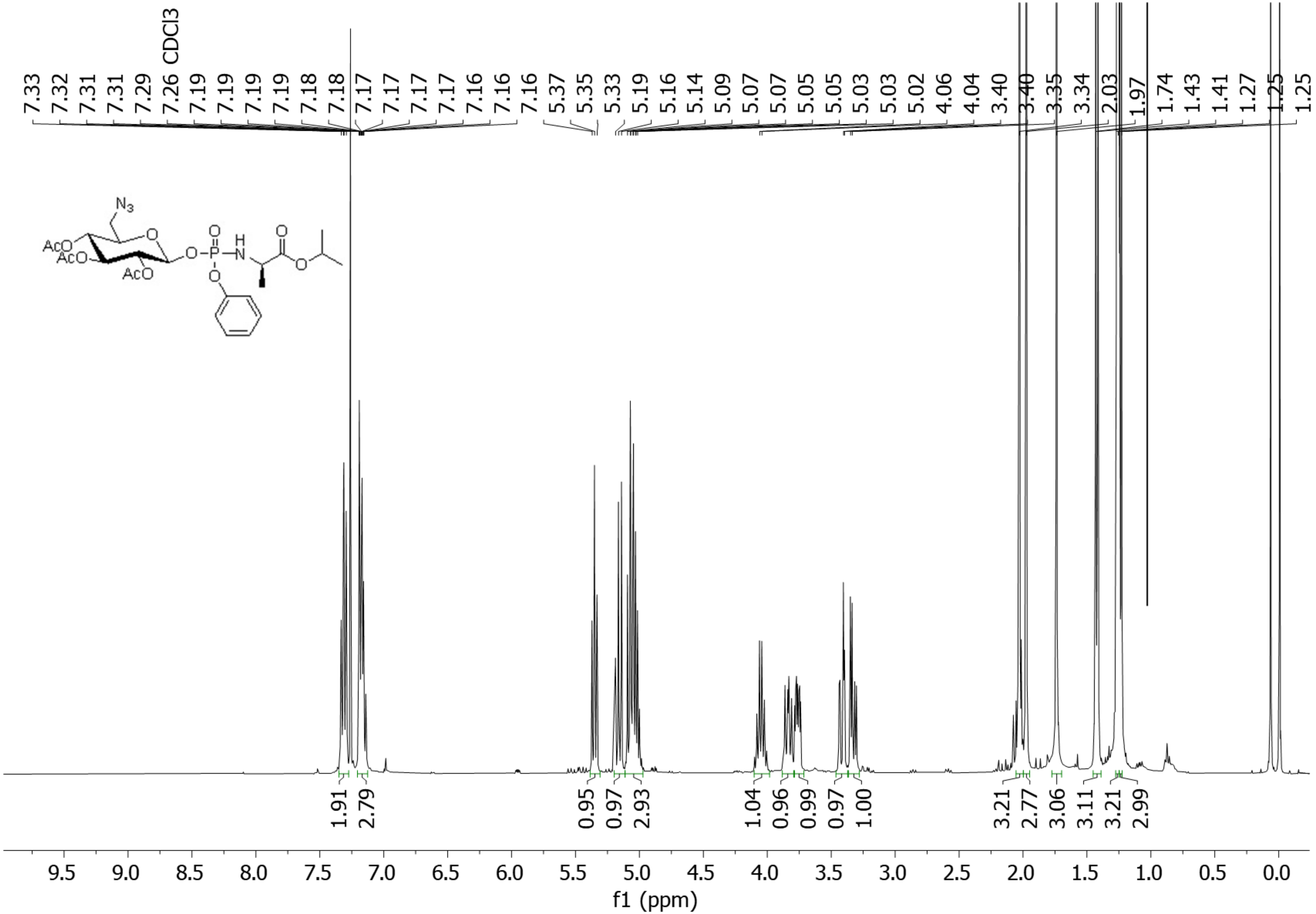

Supplementary Fig.23 ^{1}H NMR spectrum (0-9.5 ppm) of compound **2** (N-(*R*)-[phenoxyphosphinyl]-L-alanine 1-methylethyl ester)-2,3,4-tri-O-acetyl-6-azido-6-deoxy-β-D-glucopyranoside) in $CDCl_3$

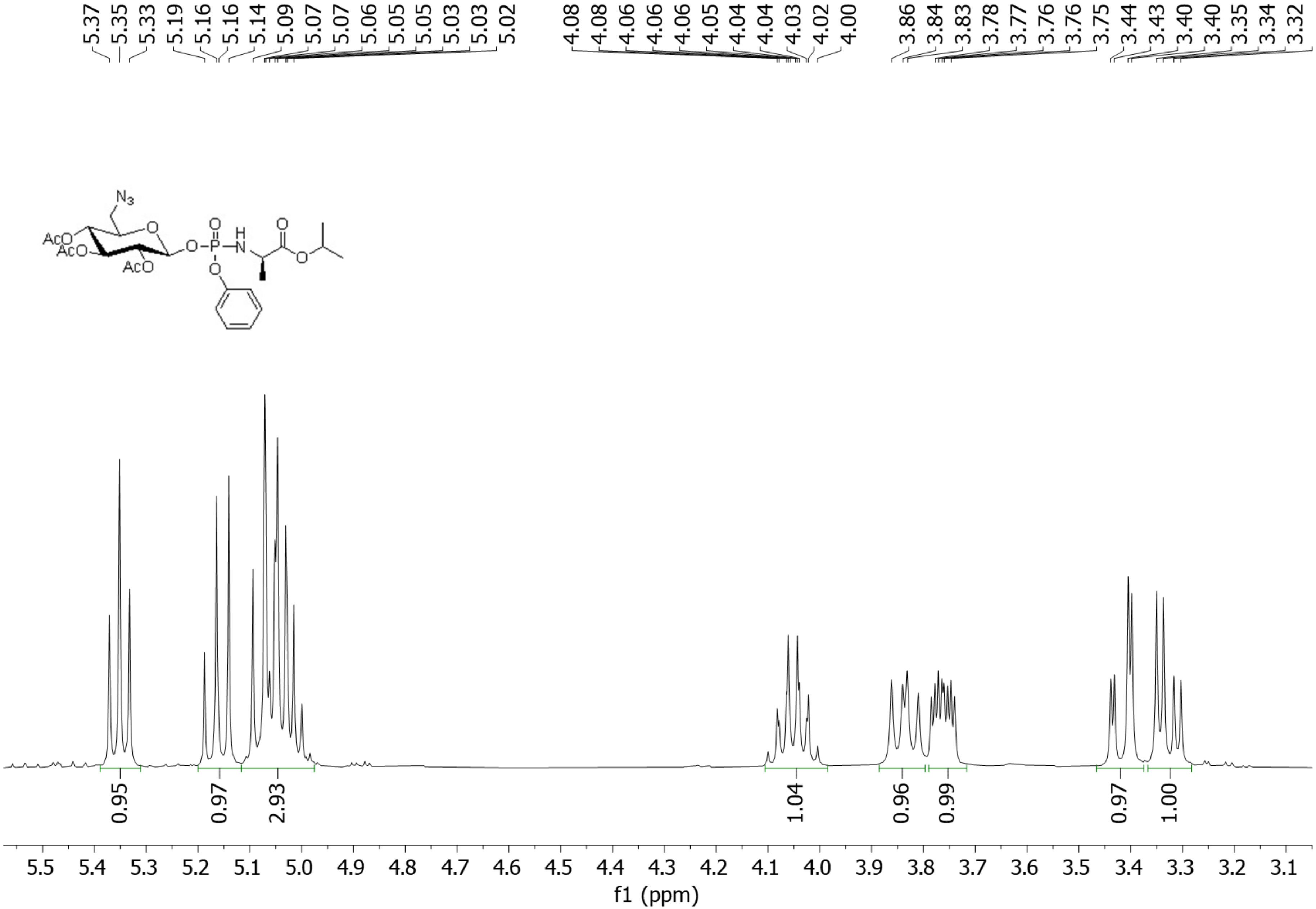

Supplementary Fig.24 ^{1}H NMR spectrum (3-6 ppm) of **2** in $CDCl_3$

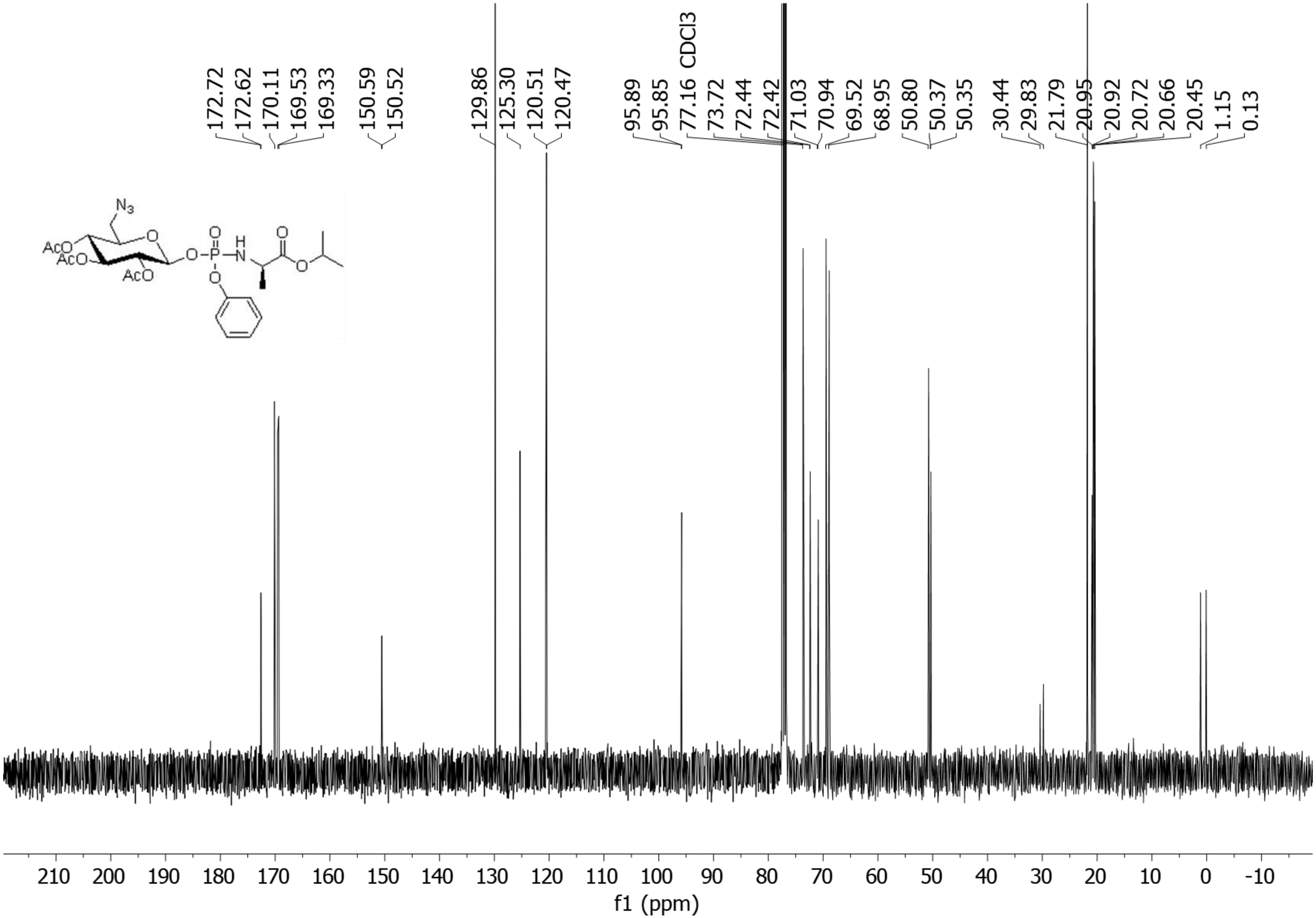

Supplementary Fig.25 ^{13}C NMR spectrum of **2** in $CDCl_3$

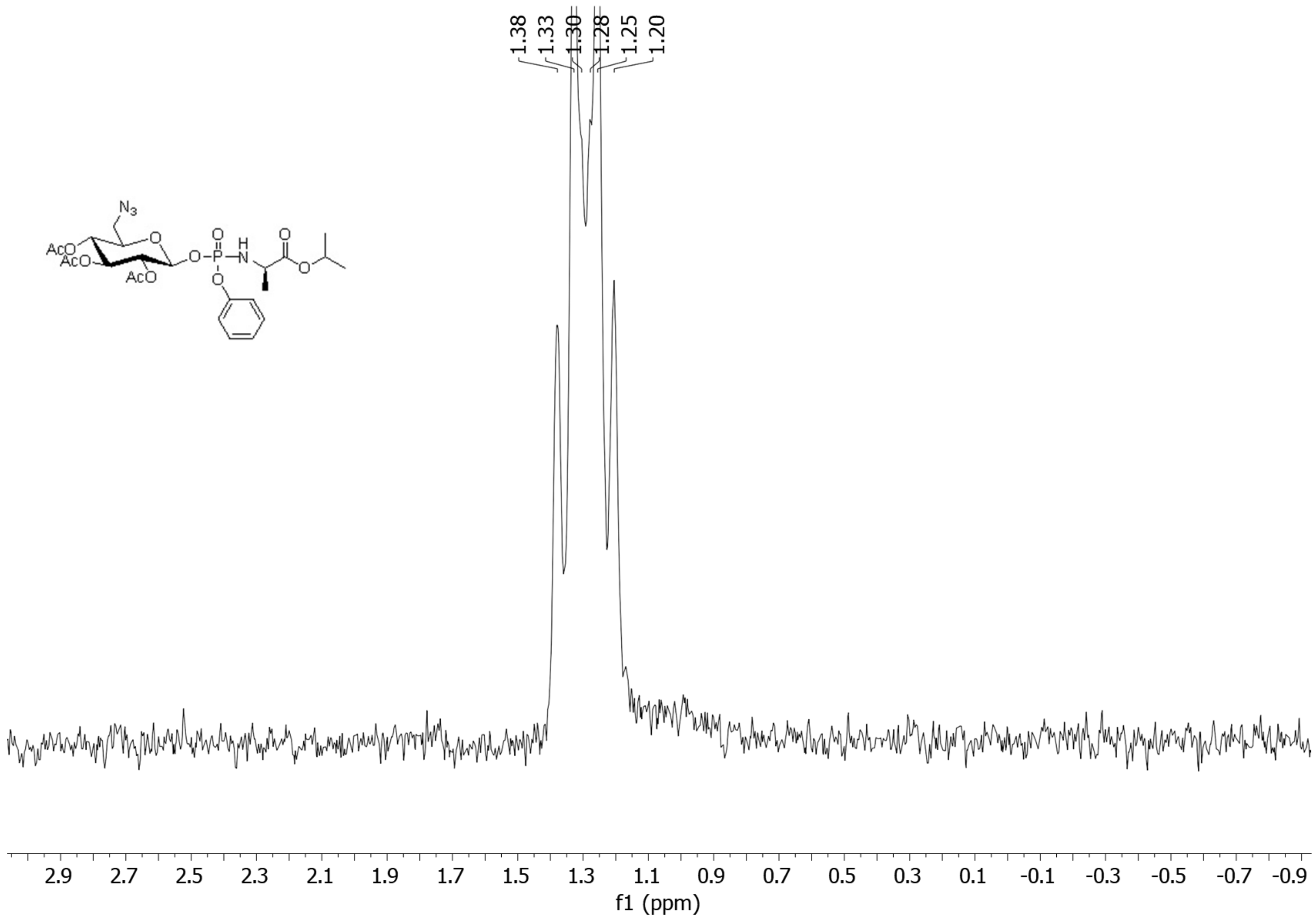

Supplementary Fig.26 ^{31}P NMR spectrum of **2**

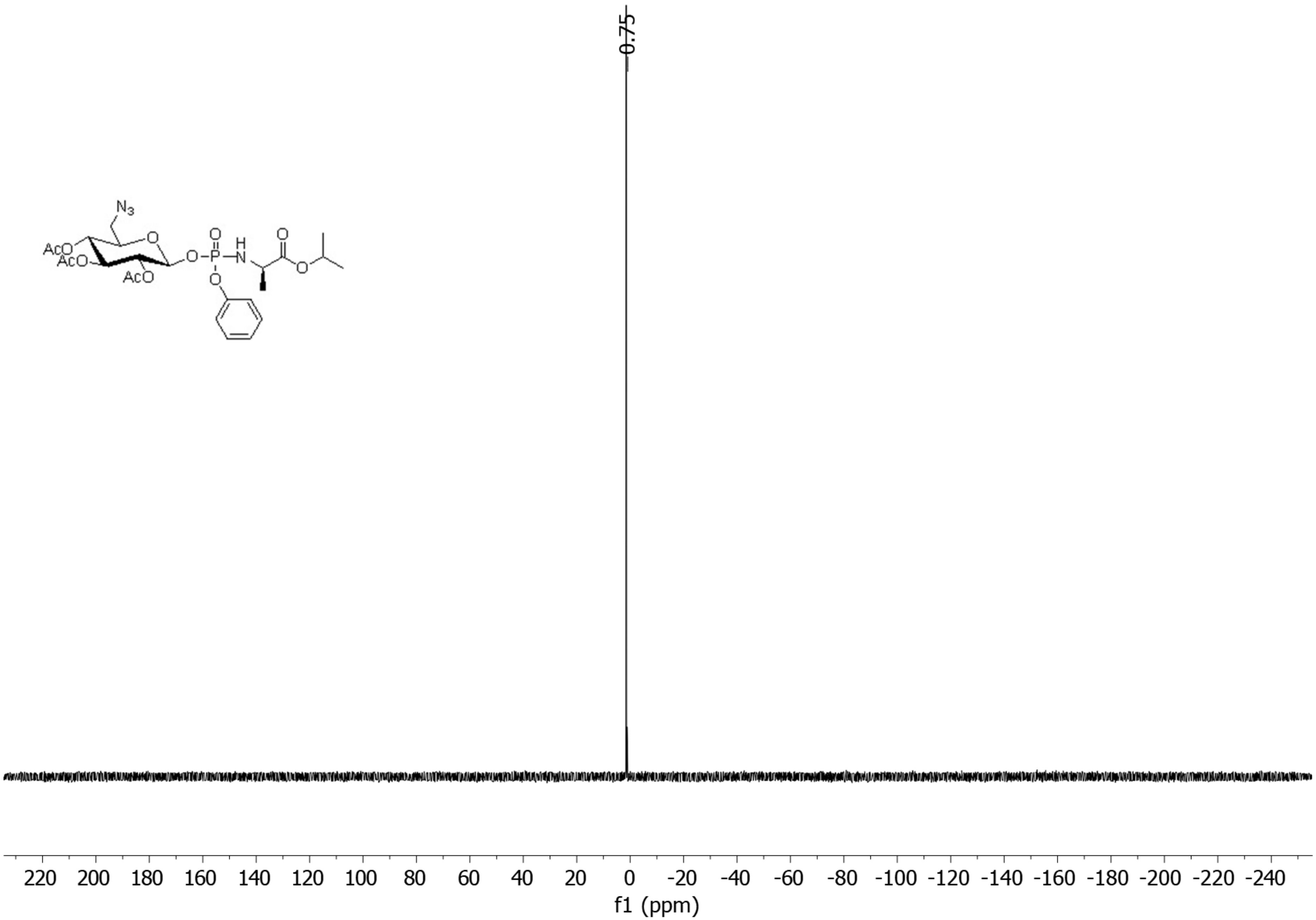

Supplementary Fig.27 Decoupled ^{31}P NMR spectrum of **2**

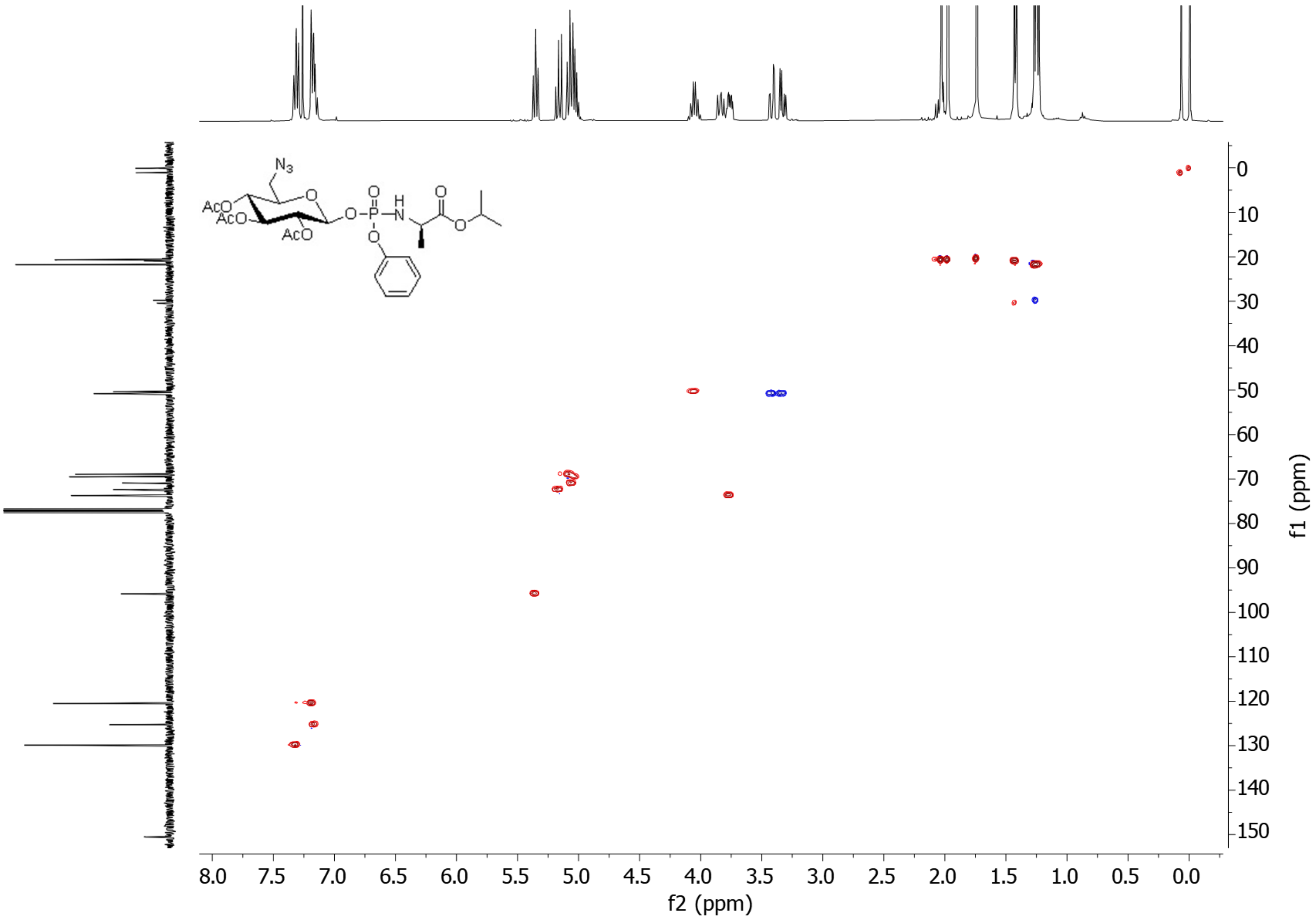

Supplementary Fig.28 ^{1}H-^{13}C HSQC NMR spectrum of **2**

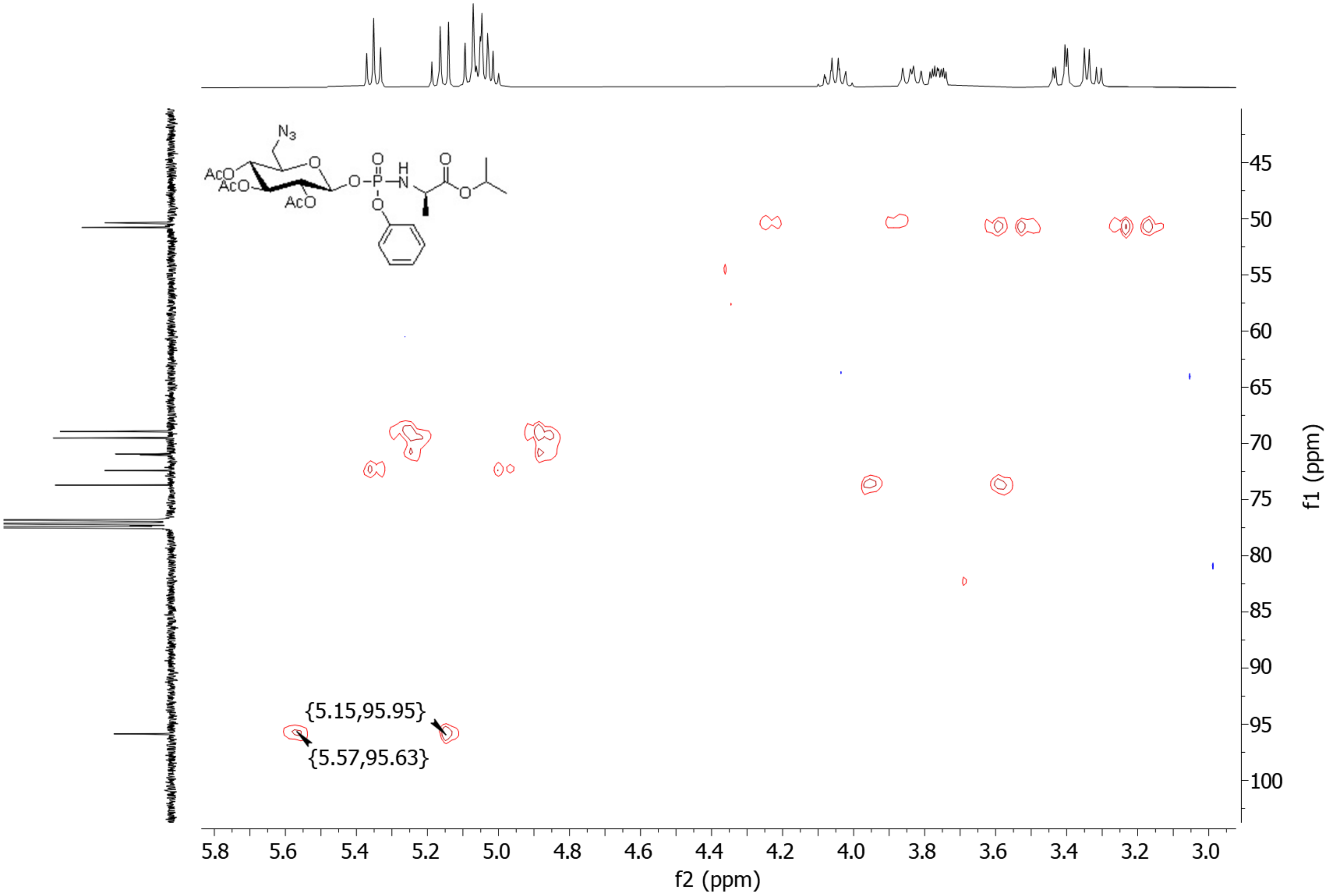

Supplementary Fig.29 ^{1}H -^{13}C HSQC NMR spectrum of **2** highlighting $^{1}J_{C\text{-}H}$

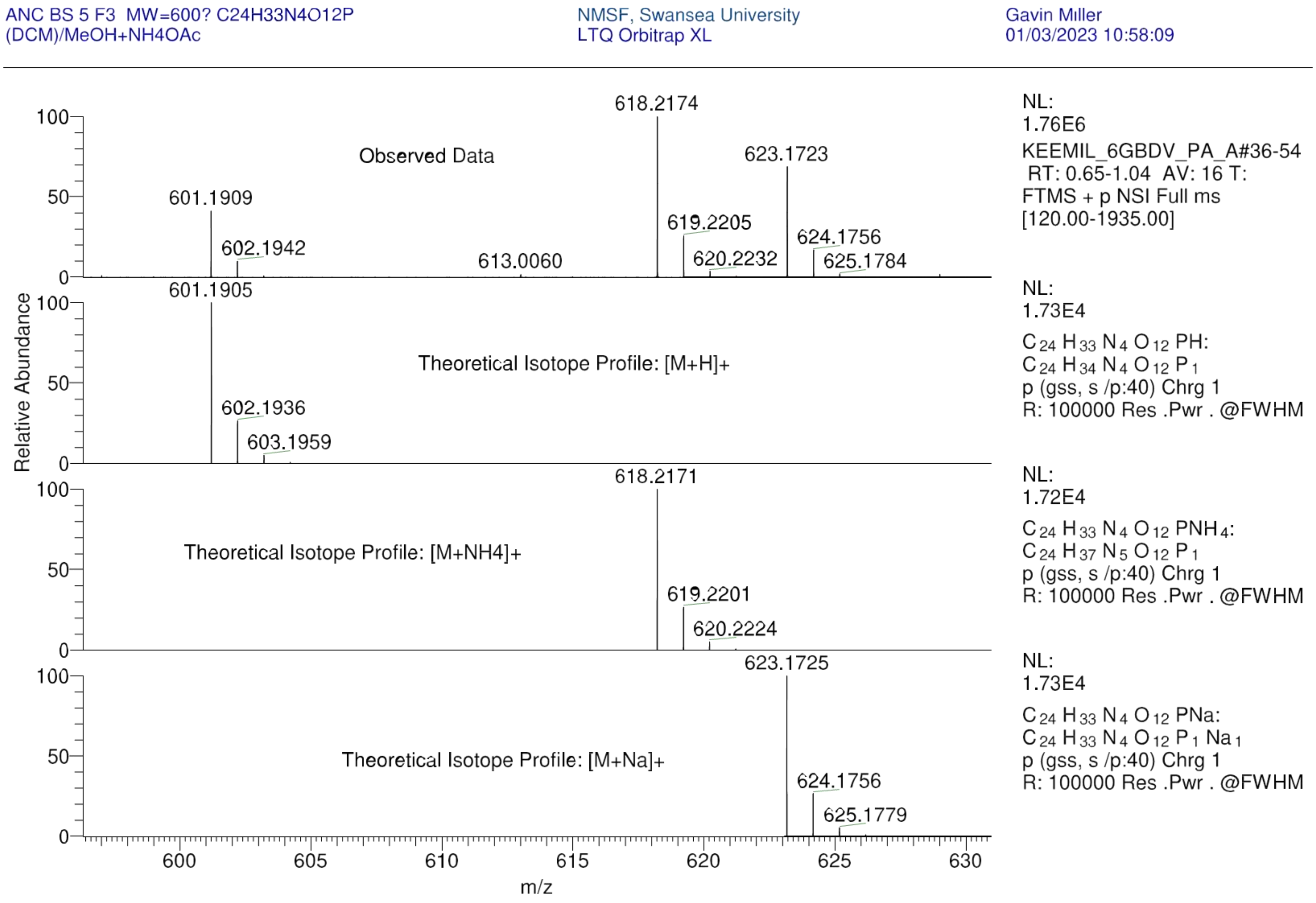

Supplementary Fig.30 High-Resolution ESI Mass Spectrum of **2**

Isotope:	Min. .. Max.		
14 N	0....16		
16 O	0....20		
12 C	0....100		
1 H	0....120		
23 Na	1....1		
31 P	0....2		
Tolerance Window:	+- 5.00 ppm		
Db/Ring Equiv:	-10.. 500	N-Rule:	Do not use
Fits:	500	Charge:	1

Mass	Theoretical Mass	Delta [ppm]	RDB	Composition
601.1909	601.1908	0.1	6.0	$C_{20} H_{37} O_{12} N_5 P_2$
	601.1908	0.1	11.5	$C_{19} H_{31} O_7 N_{12} P_2$
	601.1910	-0.2	3.5	$C_9 H_{30} O_{13} N_{16} P_1$
	601.1910	-0.2	-2.0	$C_{10} H_{36} O_{18} N_9 P_1$
	601.1911	-0.3	32.5	$C_{43} H_{25} O_2 N_2$
	601.1907	0.3	2.5	$C_{14} H_{33} O_{18} N_8$
	601.1907	0.3	8.0	$C_{13} H_{27} O_{13} N_{15}$
	601.1905	0.6	10.5	$C_{24} H_{34} O_{12} N_4 P_1$
	601.1905	0.6	16.0	$C_{23} H_{28} O_7 N_{11} P_1$
	601.1914	-0.8	-6.5	$C_6 H_{39} O_{18} N_{10} P_2$
	601.1914	-0.8	28.0	$C_{39} H_{28} O_2 N_3 P_1$
	601.1903	0.9	18.5	$C_{34} H_{35} O_6 P_2$
	601.1903	0.9	24.0	$C_{33} H_{29} O_1 N_7 P_2$
	601.1916	-1.1	25.5	$C_{28} H_{21} O_3 N_{14}$
	601.1916	-1.1	20.0	$C_{29} H_{27} O_8 N_7$
	601.1916	-1.1	14.5	$C_{30} H_{33} O_{13}$
	601.1902	1.1	15.0	$C_{28} H_{31} O_{12} N_3$
	601.1902	1.1	20.5	$C_{27} H_{25} O_7 N_{10}$
	601.1917	-1.3	23.5	$C_{35} H_{31} O_2 N_4 P_2$
	601.1900	1.5	28.5	$C_{37} H_{26} O_1 N_6 P_1$
	601.1900	1.5	-6.0	$C_4 H_{37} O_{17} N_{13} P_2$
	601.1919	-1.6	21.0	$C_{24} H_{24} O_3 N_{15} P_1$
	601.1919	-1.6	15.5	$C_{25} H_{30} O_8 N_8 P_1$
	601.1919	-1.6	10.0	$C_{26} H_{36} O_{13} N_1 P_1$
	601.1921	-1.9	7.5	$C_{15} H_{29} O_{14} N_{12}$
	601.1921	-2.0	2.0	$C_{16} H_{35} O_{19} N_5$
	601.1897	2.0	33.0	$C_{41} H_{23} O_1 N_5$
	601.1897	2.0	-1.5	$C_8 H_{34} O_{17} N_{12} P_1$
	601.1922	-2.1	16.5	$C_{20} H_{27} O_3 N_{16} P_2$
	601.1922	-2.1	11.0	$C_{21} H_{33} O_8 N_9 P_2$
	601.1922	-2.1	5.5	$C_{22} H_{39} O_{13} N_2 P_2$

Supplementary Fig.30 High-Resolution ESI Mass Spectrum of **2**

```
Data File C:\Users\P...sling\ANC BS6 iii percent purity  2023-10-03 11-36-25\beta anomer.D
Sample Name: beta anomer

    =====================================================================
    Acq. Operator   : SYSTEM                        Seq. Line :   3
    Sample Operator : SYSTEM
    Acq. Instrument : Prep LC                        Location :   P2-A-03
    Injection Date  : 10/3/2023 12:50:43 PM               Inj :   1
                                                   Inj Volume : 50.000 µl
    Method          : C:\Users\Public\Documents\ChemStation\1\Data\Aisling\ANC BS6 iii percent
                      purity  2023-10-03 11-36-25\ANC-BS-6iii Percent Purity.M (Sequence Method)
    Last changed    : 10/3/2023 11:33:37 AM by SYSTEM
    Method Info     : Polaris 5 C15-A 250 x10mm SN 593740
```

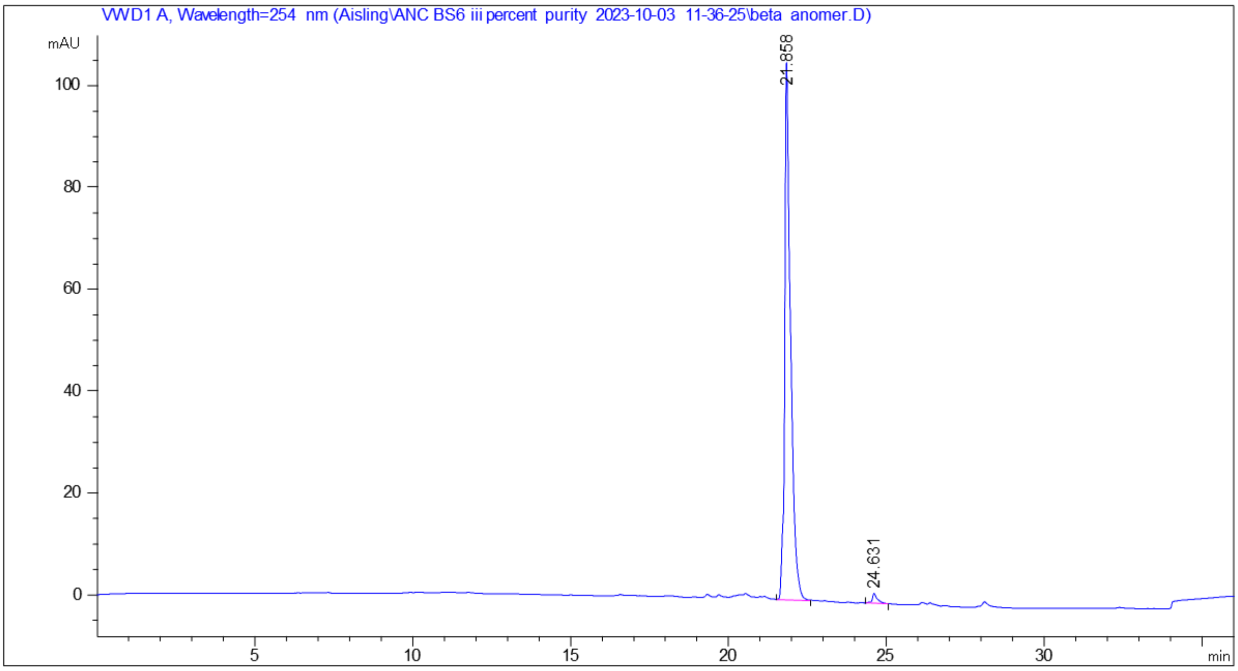


```
    ====================================================================================
                                Fraction Information
    ====================================================================================
    No Fractions found.
    ====================================================================================
    ====================================================================================
    =====================================================================
                              Area Percent Report
    =====================================================================

    Sorted By             :      Signal
    Multiplier            :      1.0000
    Dilution              :      1.0000
    Use Multiplier & Dilution Factor with ISTDs


    Signal 1: VWD1 A, Wavelength=254 nm
```


Supplementary Fig.31 HPLC chromatogram of **2**

Data File C:\Users\P...sling\ANC BS6 iii percent purity 2023-10-03 11-36-25\beta anomer.D
Sample Name: beta anomer

Peak #	RetTime [min]	Type	Width [min]	Area [mAU*s]	Height [mAU]	Area %
1	21.858	BB	0.1794	1359.55212	105.49213	98.4411
2	24.631	BB	0.1543	21.52952	1.94319	1.5589
Totals :				1381.08164	107.43532	

===

*** End of Report ***

Supplementary Fig.31 HPLC chromatogram of **2**

Additional Supplementary Figures

Unprocessed Blots for Supplementary Figure 6

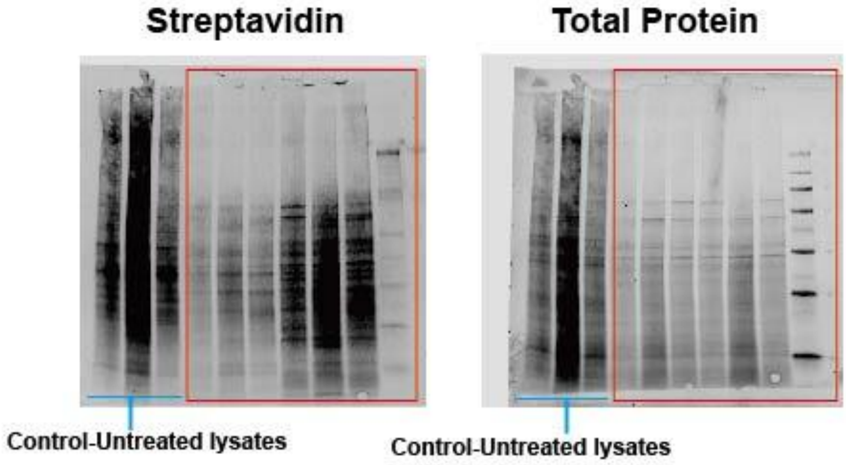

Full, unprocessed blot. Lanes 1-3 are negative controls without glycosylation treatment, which were unintentionally overloaded, leading to a high background.

For clarity and accurate representation, the overloaded control lanes (1-3) were cropped from the final figure.

Unprocessed Blots for Supplementary Figure 7

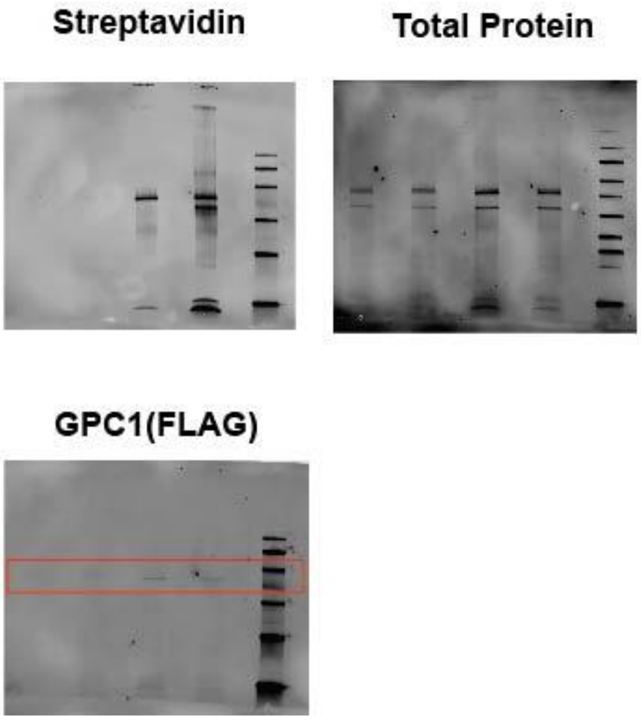

Unprocessed Blots for Supplementary Figure 8

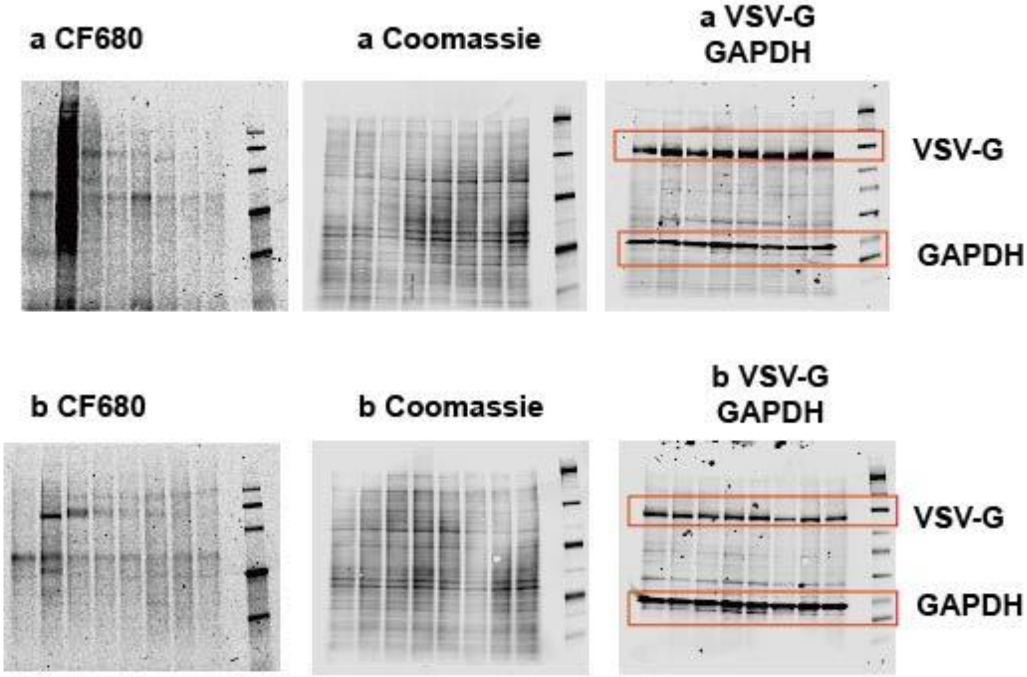

Supplement: Supplementary file 1 — Supplementary Figs. 1–13, Tables 1–3, Note, References, spectra and chromatograms (Supplementary Figs. 14–31) and unprocessed blots for supplementary figures. [file 41589_2025_2113_MOESM1_ESM.pdf]
