# Supplementary material for: Xylosyltransferase engineering to manipulate proteoglycans in mammalian cells
Source: Nat Chem Biol. 2026 Jan 20;22(4):612–21. doi: 10.1038/s41589-025-02113-w (PMC13038410; doi:10.1038/s41589-025-02113-w)
Supplement: Supplementary file 2 — Reporting Summary [file 41589_2025_2113_MOESM2_ESM.pdf]

nature portfolio

Corresponding author(s): Benjamin Schumann

Last updated by author(s): Oct 20, 2025

Reporting Summary

Nature Portfolio wishes to improve the reproducibility of the work that we publish. This form provides structure for consistency and transparency in reporting. For further information on Nature Portfolio policies, see our Editorial Policies and the Editorial Policy Checklist.

Statistics

For all statistical analyses, confirm that the following items are present in the figure legend, table legend, main text, or Methods section.

n/a	Confirmed	
☐	☒	The exact sample size (*n*) for each experimental group/condition, given as a discrete number and unit of measurement
☐	☒	A statement on whether measurements were taken from distinct samples or whether the same sample was measured repeatedly
☐	☒	The statistical test(s) used AND whether they are one- or two-sided<br>*Only common tests should be described solely by name; describe more complex techniques in the Methods section.*
☐	☒	A description of all covariates tested
☐	☒	A description of any assumptions or corrections, such as tests of normality and adjustment for multiple comparisons
☐	☒	A full description of the statistical parameters including central tendency (e.g. means) or other basic estimates (e.g. regression coefficient) AND variation (e.g. standard deviation) or associated estimates of uncertainty (e.g. confidence intervals)
☐	☒	For null hypothesis testing, the test statistic (e.g. *F*, *t*, *r*) with confidence intervals, effect sizes, degrees of freedom and *P* value noted<br>*Give P values as exact values whenever suitable.*
☒	☐	For Bayesian analysis, information on the choice of priors and Markov chain Monte Carlo settings
☒	☐	For hierarchical and complex designs, identification of the appropriate level for tests and full reporting of outcomes
☐	☒	Estimates of effect sizes (e.g. Cohen's *d*, Pearson's *r*), indicating how they were calculated

Our web collection on statistics for biologists contains articles on many of the points above.

Software and code

Policy information about availability of computer code

Data collection	UHPLC-MS (Ultra HighPerformance Liquid Chromatography) and High Performance Ion Exchange Chromatography data was recorded with Empower 3.6 (Waters);<br>Western Blot and SDS-PAGE gel images were recorded with Image studio Pro.<br>Peptide specificity was assessed using TECAN SPARKCONTROL (version 3.1).<br>Mass spectrometry glycoproteomics data to assess glycosylation site were collected using Orbitrap Eclipse Tribrid mass spectrometer (Thermo Fisher Scientific) coupled to a Dionex UltiMate 3000 HPLC (Thermo Fisher Scientific).<br>Mass spectrometry proteomics data to assess enriched glycoproteins were collected using Bruker software for TIMS TOF Pro2 coupled to Evosep One LC system.<br>GAG linker enzymatic synthesis data were collected using Agilent OpenLab software version 2.7.<br>Cell spreading was assessed using ImageJ version 1.52q.
Data analysis	WB and SDS-PAGE images were analysed with ImageStudio Lite Version 5.2<br>GraphPad Prism versions 9 and 10 were used to make bar charts and plots<br>NMR data were analysed by Mestrenova (version 14.2.2)<br>Glycopeptides were searched with ByonicTM (Protein Metrics, Cupertino, USA, Version 4.6.1)<br>DIA-NN (1.8.1) Perseus (version 2.0.11) were used for proteomics data visualization<br>Adobe Photoshop and Illustrator 2023 were used to assemble data. No non-linear image correction was performed

For manuscripts utilizing custom algorithms or software that are central to the research but not yet described in published literature, software must be made available to editors and reviewers. We strongly encourage code deposition in a community repository (e.g. GitHub). See the Nature Portfolio guidelines for submitting code & software for further information.

Data

Policy information about availability of data

All manuscripts must include a data availability statement. This statement should provide the following information, where applicable:

- Accession codes, unique identifiers, or web links for publicly available datasets
- A description of any restrictions on data availability
- For clinical datasets or third party data, please ensure that the statement adheres to our policy

Proteomics and Glycoproteomics data have been uploaded on ProteomExchange via the MassIVE server. Accession numbers: MSV000098977 (doi.org/doi:10.25345/C5HQ3SB6F); MSV000098983 (doi.org/doi:10.25345/C5R786243); MSV000098981 (doi.org/doi:10.25345/C50P0X40C); MSV000098982 (doi.org/doi:10.25345/C5VX06G5R). The authors declare that the data supporting the findings of this study are available within the paper and its Supplementary Information files. Should any raw data files be needed in another format they are available from the corresponding author upon reasonable request. Source data are provided with this paper.

Human research participants

Policy information about studies involving human research participants and Sex and Gender in Research.

Reporting on sex and gender	NA
Population characteristics	NA
Recruitment	NA
Ethics oversight	NA

Note that full information on the approval of the study protocol must also be provided in the manuscript.

Field-specific reporting

Please select the one below that is the best fit for your research. If you are not sure, read the appropriate sections before making your selection.

☒ Life sciences ☐ Behavioural & social sciences ☐ Ecological, evolutionary & environmental sciences

For a reference copy of the document with all sections, see nature.com/documents/nr-reporting-summary-flat.pdf

Life sciences study design

All studies must disclose on these points even when the disclosure is negative.

Sample size	For peptide specificity analyses, sample size were selected according to previous reports (Briggs et al., Structure, 2018);<br>For proteomics and glycoproteomics experiments, feeding conditions were chosen according to WB and SDS-PAGE analyses and sample size were decided according to previous reports (Cioce et al., Nat commun,2022);<br>Sample sizes for cell spreading experiments were chosen according to a previous report (O'Leary et al., Nat. Chem. Biol. 2022). At least six frames were counted in each replicate.
Data exclusions	One replicate dataset for proteomics analysis (Supporting Fig. 10) was excluded from analysis because loading checks indicated uneven sample quality betwween samples. In this case, the entire replicate with all samples was excluded to avoid bias. In automated cell counting (Fig. 5f), image fields that showed significant autofluorescence from the plate perimeter were not used in the analysis.
Replication	In vitro enzymatic reactions for kinetics were performed in two independent replicates with three technical replicates each, or in three independent replicates;<br>In vitro enzymatic scouting reactions to determine the optimal enzyme concentration for Michaelis-Menten kinetics was performed in three technical replicates.<br>In vitro glycosylation experiments of cell lysates and recombinant proteins were considered independent verification of the hypothesis that recombinant XT1 glycosylates proteoglycans and therefore performed in one or two independent replicates each;<br>Peptide specificity analyses were performed in three independent assays;<br>In-gel fluorescence and WB analysis of cell-surface tagging experiments were performed in duplicate;<br>Enzymatic extension of glycopeptides was performed in one experiment with a positive control and reactions containing multiple combinations of glycosyltransferases;<br>Cellular biosynthesis experiments were performed in two independent replicates;<br>Glycoproteomics experiments were subject to direct manual validation and therefore processed in one experiment;<br>Three or four independent replicates were used for proteomics experiments;<br>Assessment of heparin-SDC1 conjugation was performed as a quality control check in one experiment;<br>Cell counting was performed in three independent replicates.

Randomization	NA. The samples consist of cell lysates, culture supernatant, or purified enzymes. For technical replicates (n=3), these were aliquots from a single, homogeneous biological source allocated systematically to each experimental group. Randomization was not applicable here.<br>For biological replicates (n=3), these were samples prepared from three entirely independent cultures.These independent replicates were allocated to the experimental groups in a systematic manner.
Blinding	NA. Blinding is not applicable in our study. This is because our primary data consisted of objective, numerical output.

Reporting for specific materials, systems and methods

We require information from authors about some types of materials, experimental systems and methods used in many studies. Here, indicate whether each material, system or method listed is relevant to your study. If you are not sure if a list item applies to your research, read the appropriate section before selecting a response.

Materials & experimental systems

n/a	Involved in the study
☐	☒ Antibodies
☐	☒ Eukaryotic cell lines
☒	☐ Palaeontology and archaeology
☒	☐ Animals and other organisms
☒	☐ Clinical data
☒	☐ Dual use research of concern

Methods

n/a	Involved in the study
☒	☐ ChIP-seq
☒	☐ Flow cytometry
☒	☐ MRI-based neuroimaging

Antibodies

Antibodies used	Rabbit anti-FLAG antibody (polyclonal) (Invitrogen, Cat# PA 1-984b, RRID: AB_347227) used for WB at 1/500 dilution;<br>Rabbit anti VSV-G antibody (P5D4) (Abcam, Cat# ab50549, RRID: AB_883494) used for WB at 1/500 dilution;<br>Rabbit GAPDH antibody (EPR16891) (Abcam, Cat # ab181602, RRID: AB_2630258) was used for WB at 1/10000 dilution;<br>IRDye 800CW donkey anti-mouse (Li-Cor Biosciences, Cat #926-32212, RRID: AB_621847)was used for WB at 1/10000 dilution;<br>IRDye 680RD Donkey anti-rabbit IgG (Li-COR Biosciences; Cat #926-68073, RRID:AB_10954442) was used for WB at 1/7500
Validation	Antibodies have been validated in WB analyses including positive and negative controls of CHO pgsA-745 transfected cells.

Eukaryotic cell lines

Policy information about cell lines and Sex and Gender in Research

Cell line source(s)	Expi 293F (ThermoFisher A14527); K562 (ATCC, CCL-243); CHO pgsA-745 (ATCC CRL-2242K562); MDA-MB-231 (ATCC, CRM-HTB-26); CHOZN GS-/-, CHO KO Xylt1 and CHO KO Xylt2 (Copenhagen Center for Glycomics, University of Copenhagen); K562 KO XYLT1 and K562 KO XYLT2 are generated in this work; BH and WT XT1/XT2 transfected cells are generated in this work.
Authentication	All cells used in the manuscript except for MDA-MB-231 were authenticated by The Francis Crick Institute Cell Services STP by STR profiling (for human lines) and species identification for validation. MDA-MB-231 cells the same as in O'Leary et al., Nat. Chem. Biol. 18 (2022).
Mycoplasma contamination	All cell lines tested negative for mycoplasma contamination at the Francis Crick Institute Cell Services Science Technology Platform, or manually by the Mycoprobe detection kit (R&D Systems).
Commonly misidentified lines<br>(See ICLAC register)	None
