# Supplementary figures and images for: Xylosyltransferase engineering to manipulate proteoglycans in mammalian cells
Source: Nat Chem Biol. 2026 Jan 20;22(4):612–21. doi: 10.1038/s41589-025-02113-w (PMC13038410; doi:10.1038/s41589-025-02113-w)

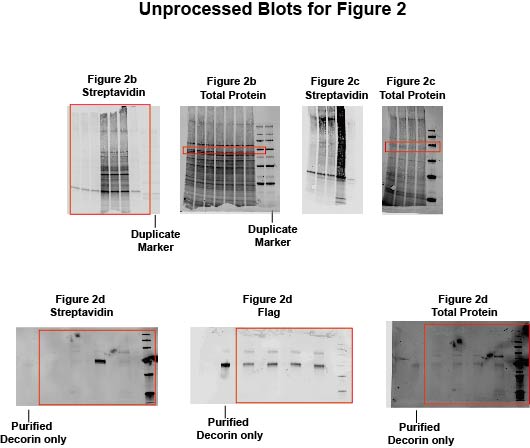

Supplement: Supplementary file 15 — Unprocessed blots for Fig. 2b–d. [file 41589_2025_2113_MOESM15_ESM.jpg]

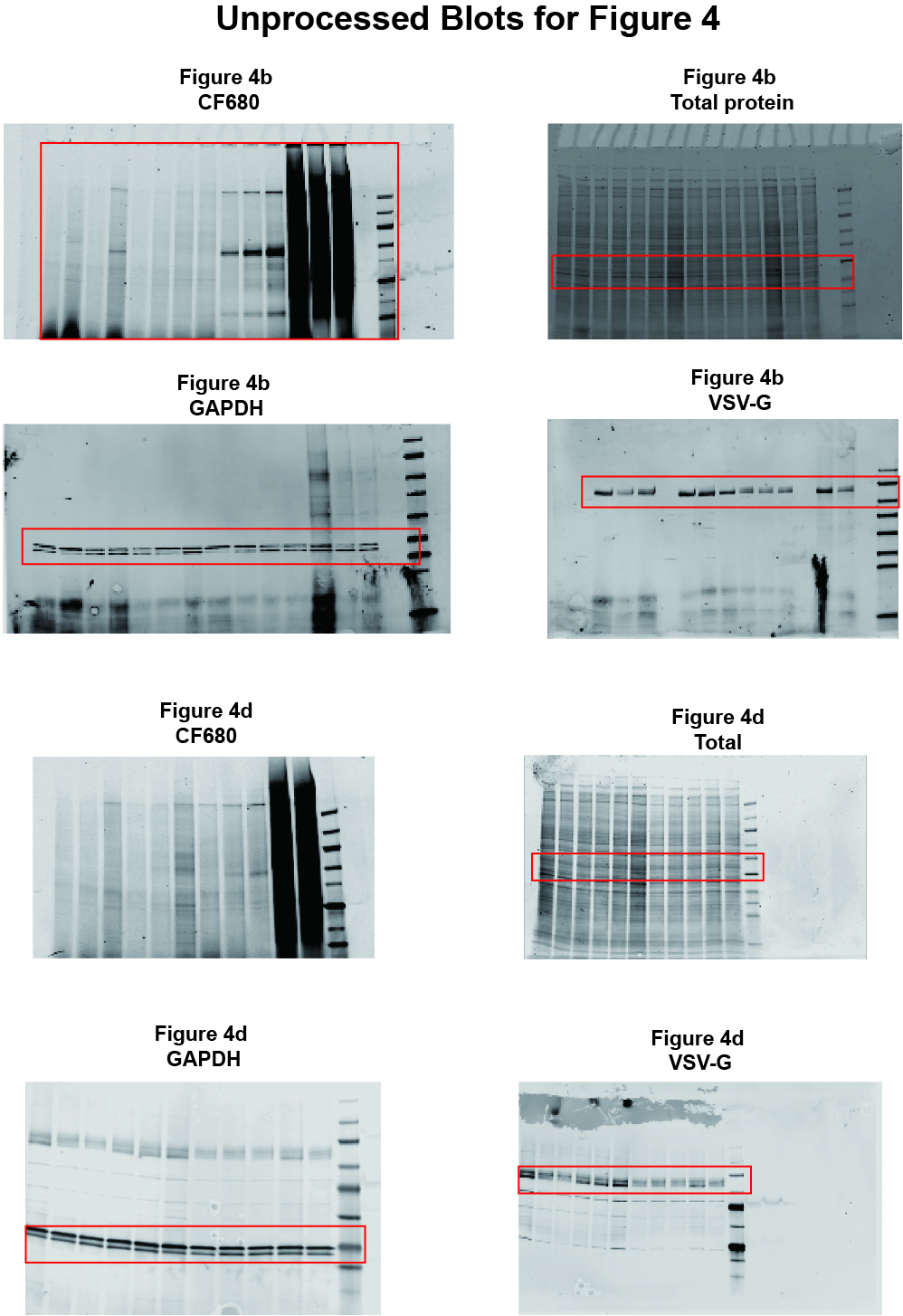

Supplement: Supplementary file 17 — Unprocessed blots for Fig. 4b,d. [file 41589_2025_2113_MOESM17_ESM.jpg]
